# Estimating the impact of discharge to nursing home on readmission and mortality: a propensity score matched analysis

**DOI:** 10.1186/s12877-026-07761-8

**Published:** 2026-07-04

**Authors:** Robert S Kristiansson, Wilhelm Linder, Ulrika Winblad, Douglas Spangler

**Affiliations:** Department of Public Health and Caring Sciences, Uppsala University, Uppsala, Sweden

**Keywords:** Nursing homes, Home care, End-of-life, Propensity score matching, Readmission, Long-term care

## Abstract

**Background:**

The hospital discharge process entails substantial challenges, particularly among older multimorbid patients with a high risk of subsequent readmission and/or death. Discharging hospitals often face a decision regarding whether such patients should be discharged to a short- or long-term stay at a nursing home, or sent home with community-based support services. Therefore, the aim of this study was to investigate the impact of these forms of post-hospital care on readmissions and mortality.

**Methods:**

We used data from national Swedish health-, and social care registries from 2015 to 2019 to perform a retrospective analysis using a target trial emulation design. The study included patients above 65 years of age with multimorbidity who were community dwelling prior to hospitalization and subsequently discharged to either a nursing home or back to their own residence with formal home care interventions. To estimate the average treatment effect on the treated of nursing home vs. in-home care, each hospital discharge to a nursing home was matched to a discharge to home care using a propensity score generated by a gradient boosting model based on the patient’s previous medical and social care history. Outcomes were assessed using a double robust approach consisting of survival analysis with statistical adjustment for residual confounding.

**Results:**

Hospital discharges to nursing homes had a lower risk of hospital readmission across 7-, 30- and 90-day endpoints, with hazard ratios of 0.64, 0.77, and 0.85 respectively. Short term (7 day) mortality was similar between hospital discharges to home care and nursing homes (HR 0.92), but higher at 30 and 90 days among nursing home discharges (HR 1.32 and 1.54, respectively).

**Discussion:**

Our findings suggest benefits of nursing home care in terms of reduced readmission, especially in the short-term. While short term mortality was similar between groups, longer term mortality was higher for nursing home discharges, which may be the result of differences in care practices or unmeasured confounding. Taken together, the study suggests that the strongest impact of NH care is in the short term, while further research is needed to validate the results, particularly regarding longer-term mortality.

**Supplementary Information:**

The online version contains supplementary material available at 10.1186/s12877-026-07761-8.

## Background

It is well known that the number of patients with chronic diseases associated with aging is growing [1]. As a result, rates of hospital admissions and unplanned readmissions are increasing, presenting a challenge to the provision of high-quality and cost-effective health care [2–4]. Simultaneously, many countries are decreasing the number of hospital beds, with several national initiatives aimed at shifting a greater share of healthcare provision away from hospitals [5]. Care providers must therefore collaborate more efficiently to maintain quality, highlighting the importance of well-planned transitions from the hospital to lower levels of care. In this paper, we compare two common forms of care for older patients after hospital discharge: Nursing Home (NH) care consisting of either short- or long-term care at a residential facility and Home Care (HC) consisting of health- and social care services in the patients’ home.

Previous research has shown that the main predictors of admission to a NH over HC are cognitive status, absence of family caregiver support, and disease severity [6, 7]. There are also often substantial differences in the type and intensity of care that is provided in NHs compared to what is provided in patients’ homes. In NHs, basic nursing care is usually available around the clock, with an employed or contracted physician on call. Care is integrated and typically includes both planned nursing care and regular medical rounds by a physician. Care for patients living in their own homes is, from an organizational perspective, often more complex. Different models of HC exist across countries, but day-to-day care is typically provided by non-nurse care providers assisting with activities of daily living. Contact with nurses for medical treatment is generally less frequent. Medical support is often provided through general practitioners (GPs) or equivalent services such as medical home care teams, with previous studies indicating that there is a risk of delayed medical care during the post discharge period for this patient group [8]. Thus, both acute and longer-term health issues may be easier to detect in NHs and may receive earlier treatment than health issues emerging in patients living at home. Similarly, the efforts undertaken at NHs might prevent falls or medication errors which could result in decreased adverse outcomes [9, 10].

For some patients it is difficult to determine whether their needs have reached the threshold for NH care or if HC is sufficient. This is further complicated as the decision to admit a patient to a NH must also take into account the individual’s preference for autonomy (i.e., living in his or her private residence), existential concerns and the potential of cognitive and/or health decline sometimes seen in patients relocating to NHs [11–13]. As such, there may be a subset of patients where there is substantial ambiguity regarding the relative benefit of NH compared to HC.

One goal of well-planned, coordinated care for older adults is to keep the patient from the hospital if there is not an absolute need for specialized health care. While there is substantial variation among NHs, previous studies have reported significant reductions in hospital admissions from NHs that implement formal care plans [14]. Variation also comes from frailty among NH-residents as it is strongly associated with higher health care utilization, and from interventions outside of NH services [15]. There are furthermore substantial differences in number of transitions between health care providers due to patient characteristics such as cognitive impairment, age, multimorbidity, and walking speed [16].

Regardless of whether a patient is discharged home or to a nursing home it is crucial that health care is provided in a timely manner during the first weeks of the discharge process [17]. Patients are nonetheless often re-hospitalized in the immediate post-discharge period, even when it could potentially have been avoided [16, 18]. It is therefore common to track readmission rates, particularly within 30 days, as an indicator of health care quality [19]. For instance, a study from Sweden indicated that patients discharged to HC had an increased risk of readmissions within 30 days compared to patients discharged to NHs (adjusted OR = 1.62) [20]. Similar findings were described by Werner et al., where patients discharged to home health care had 5.6% more readmissions within 30 days compared to patients discharged to NHs [21]. Furthermore, previous studies indicate that all-cause hospitalizations typically increase in for patients during the year prior to nursing home admission and subsequently decrease, indicating that prior health care utilization is an important pre-treatment variable in studies comparing post hospital discharge rates between private residency with HC and NH-settings [22].

In addition to readmissions, it also common to track mortality as it is often considered the “gold standard” outcome given its objective nature. However, in contrast to the previous findings on readmissions, the effects on mortality are less clear. For instance, the study by Werner et al. found no differences in mortality at 30 days post discharge between HC and NH [21], whereas another study reported decreased mortality for patients discharged to intensive HC compared to NHs *(OR 0.49)* [20].

### Aim

To estimate the impact of discharge from hospital to nursing home vs. in-home care on subsequent unplanned readmission and all-cause mortality rates. Hypotheses relating to differences over short (7-day), medium (30-day) and longer term (90-day) outcomes were evaluated for each outcome.

## Methods

### Study setting

The Swedish health care system is divided into 21 regions and 290 municipalities. The regions organize, finance, and provide most primary, secondary, and tertiary care, while the municipalities deliver primary health- and social care to older adults and those with functional impairment. Health care is tax-funded with universal health coverage, and individuals have the same entitlements to services in hospitals, NHs, and HC [23]. When a patient with multimorbidity and/or complex needs is admitted to a hospital, regions and municipalities collaborate to assess the future health care needs of the patient [24]. This process aims to ensure appropriate care for the patient and to facilitate discharge as soon as hospitalization is no longer considered necessary. For older patients with multimorbidity, the main alternatives for patients that require support post-discharge are NHs or discharge home with social HC and/or home health care services.

In NHs, daytime nurses typically work on-site, but outside office hours they often provide care on-call across multiple NHs, with non-nurse staff providing care on-site at the NH [25]. Medical services (e.g., clinical rounds by physicians) are provided by the regions, with the extent of services regulated by formal agreements between regions and municipalities. HC is generally provided by a municipal team led by a nurse, who assists the patient with health care needs and coordinates physician visits carried out by the patient’s general practitioner (GP). Patients with HC in Sweden are at substantial risk of hospitalization as their mean age is 85, and 57% have > 5 chronic diseases, and are admitted to hospital 1.6 times as often than a matched group without HC [26].

The formal decision regarding whether a patient will be discharged to HC or NH is taken by needs assessors in the patient’s municipality of residence [27]. Importantly, previous publications by the Swedish National Board of Health and Welfare show structural variations between municipalities regarding NH-capacity, with up to a third of municipalities having a lack of NH-beds whereas only a small minority of municipalities have excess capacity [27]. In practice, needs assessor balance patient needs with available resources, implying that the decision to discharge a patient to NH or HC is to some extent subject to variability that is related to structural factors such as current availability of NH-beds rather than the patient’s care needs [27].

### Study design

A hypothetical randomized controlled trial was designed, whereby community-dwelling multimorbid patients admitted to hospital and eligible for elder care at a NH (65 years old) would be randomized at discharge to post-discharge care at a NH (treated), or to continue living at home with in-home care (untreated). Inclusion and exclusion criteria were specified to correspond to the target trial, and propensity scores were used to match each treated hospital discharge with an untreated hospital discharge with a similar propensity score [28].

### Data

The study data is drawn from a cohort consisting of individuals living in Sweden who were admitted to a hospital during 2015–2019, were above 65 years old at time of hospital admission, and had at least 2 registered ICD-codes in at least one admission. In total, the cohort used to construct the study sample consisted of 1,032,506 individuals with 2,852,592 hospital admissions, with hospital admissions serving as the unit of analysis. Data were collected from Swedish registries managed by the National Board of Health and Welfare, including the outpatient- and inpatient care registries, social care registry, and cause of death registry [29, 30].

### Study sample

The study sample was derived from the cohort using a target trial emulation approach which mimics a hypothetical randomized controlled trial. We defined the target trial as considering all hospital admissions of multimorbid patients (2 or more documented ICD-10 codes) over 65 years of age discharged between January 1, 2016 and October 31, 2019 as potentially eligible for randomization at discharge to either NH care including both short- and long-term stays (treated) or to the patient’s private residence supported by home care (untreated). To match this target trial, any hospital discharges meeting the following criteria were excluded: A planned admission (unlikely to be subject to a decision regarding follow-up social care), admissions missing discharge or hospital ward data (unable to reliably assess target trial arm), admissions where the patient was not discharged home or to a NH (in-hospital death or discharge to another in-patient facility), admissions with a less than 3-day (unlikely to have time to perform a discharge planning process resulting in NH placement) or longer than a 90-day stay (rare, and likely involving palliative/intermediate care stays), hospital admissions where the patient was admitted from a nursing home (unlikely to be assigned to home care), hospital admissions where the patient was readmitted within one day (potential data quality issues, and unlikely to be exposed to treatment), hospital discharges without any form of social care (not relevant given the desired treatment contrast), or disagreement regarding the social care status of the patient at dispatch between the relevant government registries used to conduct the study (indicative of data quality issues, and the hospital admission thereby being at risk for misclassification), and admissions from home of patients previously discharged to NH (likely the result of documentation errors). The sample generated through the application of these criteria was considered to consist of those most likely to be exposed to random variability in treatment assignment at hospital discharge. An additional criterion was applied at the matching stage to include only a single hospital stay from a given patient in order to avoid correlations between observations.

### Propensity score matching

Propensity score matching was used to match each treated case to an untreated case based on their likelihood of being discharged to NH vs. HC. Propensity score matching rather than weighting was used to reduce the influence of individuals with extreme propensity scores and to obtain a well-balanced, clinically comparable study population. Given the observational design and overlap limitations, matching was considered more robust and interpretable, and the large sample size meant that the additional statistical power afforded by weighting was not necessary.

Scores were calculated using gradient boosting (R package *XGBoost*) applied to an extensive set of pre-treatment variables [31]. The first category of variables included demographic/socioeconomic factors of age, sex, civil status and country of birth. The second category of variables consisted of clinical factors including primary and secondary diagnosis at discharge coded at full precision per the ICD-10 standard, primary diagnosis at previous hospital discharge, medical/surgical interventions coded per the Swedish KVÅ standard, number of diagnoses, and number of medical/surgical interventions. The third category of variables consisted of the administrative health- and social care variables including length of stay in hospital, days since last hospital visit, length of stay in hospital during previous hospital visit, number of planned/unplanned hospital admissions during the previous 12 months, number of months with home health care / municipal home service before index admission, and number of planned / unplanned ambulatory care visits during the previous year. The final category of variables consisted of geographic and temporal variables such as region, municipality, hospital, weekday and week of year.

A total of 1686 parameters (with each level of the categorical predictors constituting one parameter) were included in the final propensity score model. Hospital discharges where the patient was discharged to NH care were matched to discharges to home care services using the nearest neighbor method with a caliper of 0.05 on a probability scale, with exact matching performed on patient sex and Major Diagnosis Code group (R package *MatchIt*) [32]. The Average Treatment effect on the Treated (ATT) estimand was targeted, employing a 1:1 intervention-control ratio, including each distinct patient at most one time. To avoid overfitting, propensity score predictions made in cross-validation folds were used. A full list of the employed predictors with measures of variable importance and their marginal contribution to the propensity score as measured by SHAP values, as well as partial dependence plots for the five most influential variables is available in Supplementary materials 1 – Propensity score variable description [33].

### Statistical analysis

Descriptive statistics were generated using appropriate measures of central tendency and 95% confidence intervals based on the percentiles of 1000 bootstrap replicates. A survival analysis approach was used to analyze the data, with cumulative incidence rate curves used to visualize the results and to detect violations of the proportional hazards assumption. Hazard ratios were reported at 7-, 30-, and 90-day intervals. Readmission rates were analyzed using competing risk regression accounting for mortality as a competing risk using the Fine and Grey method (R package *cmprsk*) [34]. Mortality was analyzed using Cox Proportional Hazards and Cumulative incidence functions were estimated using the R *survival* package [35]. To account for residual covariate imbalance, final reported hazard ratios were estimated using a double-robust approach whereby readmission and mortality were predicted using the same methodology used to generate propensity scores for discharge to NHs described previously. Predictions for each investigated outcome and follow-up period were then included as a covariate in the respective hazard ratio estimation models. Sensitivity analyses were performed to evaluate the impact of various modifications to the methodology used in the study. R version 4.5.2 was used to perform all data analysis [36]. All R source code used to analyze the data and generate the results reported here are available in Supplementary materials 1.

## Results

### Descriptive statistics

After applying inclusion and exclusion criteria, the dataset consisted of 413 073 hospital admissions. For a detailed description of the numbers excluded by each inclusion and exclusion criteria, see Table 1.

**Table 1:** Exclusion criteria applied to the study population

Number included	Number excluded	Description
2 852 592	NA	Patients over 65 with 2+ ICD codes
2 141 053	711 539	Admission within study timeframe (2015–2019)
1 738 476	402 577	Unplanned admission
1 738 063	413	Missing discharge/admission/hospital data
1 597 629	140 434	Discharge to home or NH
1 290 148	307 481	Care duration < 3 days
1 288 843	1 305	Care duration > 90 days
1 287 612	1 231	Missing ward information
1 287 108	504	Same day readmission
1 188 724	98 384	Patient admitted from home
1 001 640	187 084	Agreeement re: admission and discharge between data sources*
434 405	567 235	Discharged home without care services
413 657	20 748	Single diagnosis only
413 073	584	Admission from home following previous discharge to NH*
413 073	0	Final

*Omission of these data quality criteria were assessed in a sensitivity analysis

In total, 69 890 episodes discharged to NH (treated), and 343 183 episodes discharged to HC (untreated) were identified. Of the 69 890 hospital discharges to NH and 343 183 discharges to HC, 36 111 discharges from each group were matched to a corresponding discharge in the other arm, with the remainder excluded due to repeat patient admissions, or owing to the algorithm being unable to identify a sufficiently close match.

**Fig. 1:**
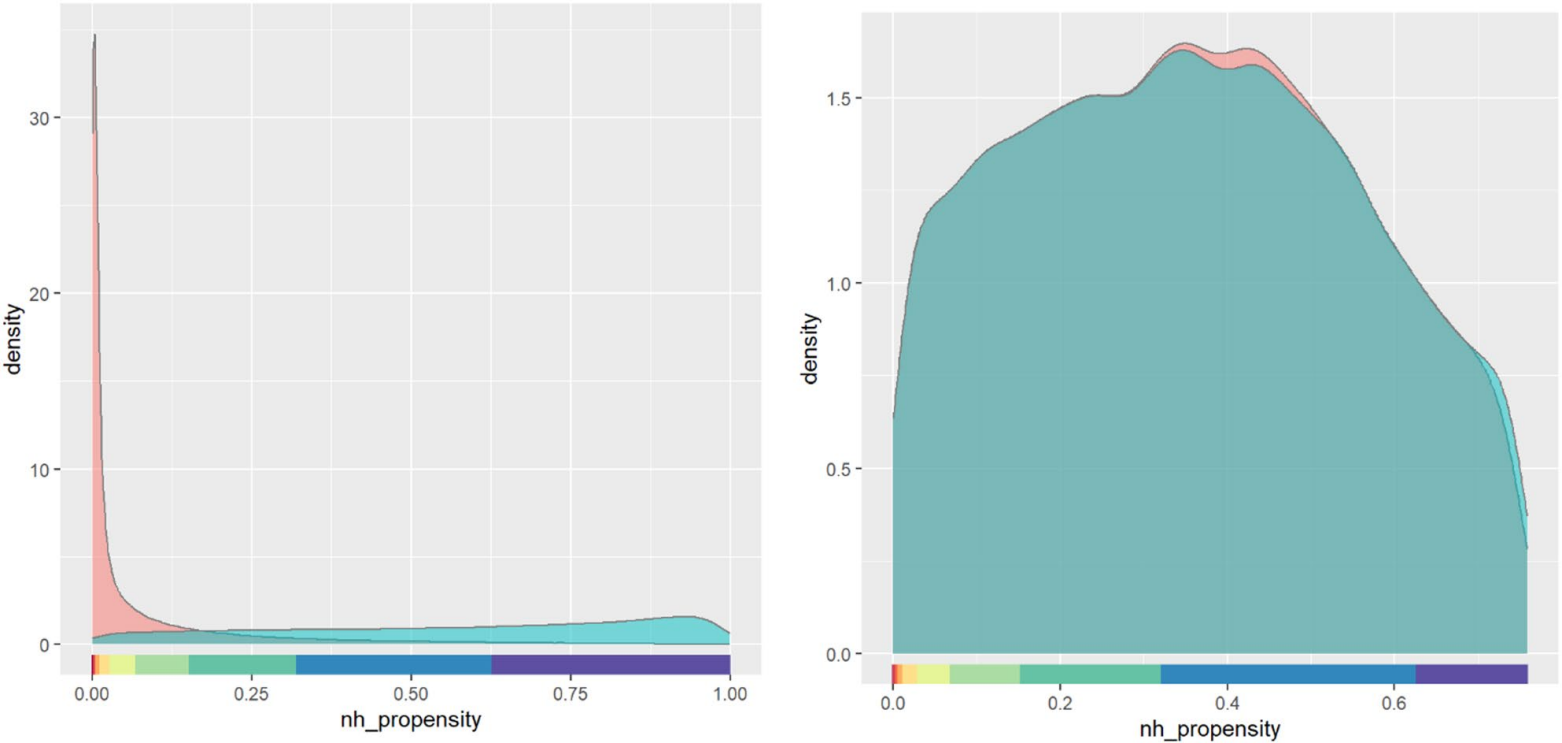
Propensity score distribution in raw and matched sample. Red indicates observations discharged to HC, blue to NH care. The colored rug indicated deciles of the propensity score in the study population

**Fig. 2:**
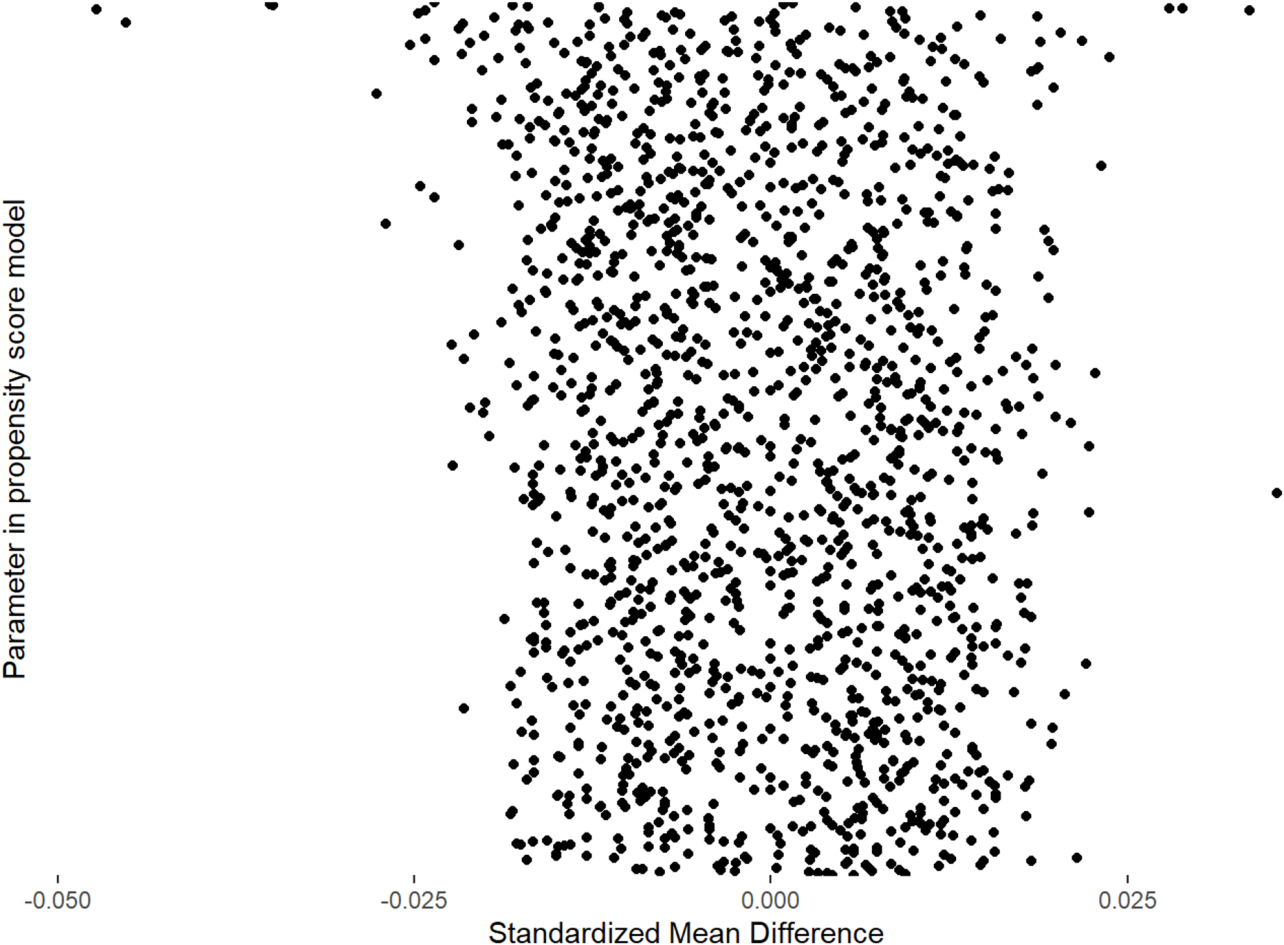
Standardized mean differences of all parameters (predictors) included in the propensity score model. Each dot represents a predictor used in the machine learning propensity score model. Numerical values and 95% confidence intervals for all predictors may be found in supplementary materials 1 – Propensity score variable description

The propensity score explained 56% of the variation (conditional Nagelkerke pseudo-R2 value) in being admitted to NH vs. HC. The distribution of propensity scores in the raw (pre-matching) and matched sample can be seen in Fig. 1. The raw sample indicates that a large share of discharges had relatively low or high propensity scores with the overlap occurring mainly between propensity scores of 0.0 to 0.5. To assess covariate balance, Standardized Mean Differences (SMDs) were calculated for all parameters used in the prediction model comparing the NH and HC group. After matching based on propensity scores, evaluation of SMDs suggested that the matching process had resulted in good covariate balance as seen in Table 2. As seen in Fig. 2, all covariate SMDs were well below the often-used threshold of 0.1, indicating that the matching process resulted in good balance across all measured predictor variables.

**Table 2:** Characteristics of study sample with 95% confidence intervals

	Raw HC	Raw NH	Matched HC	Matched NH	Standardized Mean Difference of matched sample (Cohens D)
n	343,183	69,890	36,111	36,111	NA
Age (mean)	82.02 (82-82.05)	83.97 (83.91–84.03)	82.54 (82.46–82.63)	82.82 (82.72–82.9)	-0.035 (-0.049- -0.02)
Female (%)	0.59 (0.59–0.59)	0.59 (0.59–0.59)	0.58 (0.57–0.58)	0.58 (0.57–0.58)	0*
Length of stay (days, mean)	10.07 (10.04–10.09)	15.97 (15.85–16.06)	14.36 (14.24–14.49)	14.35 (14.24–14.47)	0.002 (-0.013-0.016)
Number of medical / surgical procedures (mean)	2.56 (2.55–2.57)	3.09 (3.06–3.11)	3.09 (3.06–3.12)	3.06 (3.02–3.1)	0.009 (-0.005-0.024)
Number of diagnoses (mean)	5.74 (5.73–5.75)	6.03 (6-6.05)	5.67 (5.64–5.7)	5.74 (5.71–5.77)	-0.025 (-0.039-0.01)
Days since last hospital admission (mean)	90.85 (90.44–91.31)	83.86 (82.9-85.25)	79.11 (77.02–80.73)	78.69 (76.76–80.37)	0.004 (-0.02-0.028)
Number of hospital inpatient admissions last 12 months (mean)	1.65 (1.64–1.66)	1.05 (1.04–1.06)	0.76 (0.74–0.77)	0.82 (0.81–0.84)	-0.045 (-0.06-0.031)
Number of unplanned hospital inpatient admissions last 12 months (mean)	1.48 (1.47–1.48)	0.95 (0.94–0.96)	0.66 (0.65–0.67)	0.72 (0.71–0.74)	-0.047 (-0.062-0.033)
Months with home health care services before hospitalization (mean)	3.96 (3.93–3.99)	0.28 (0.26–0.3)	0.48 (0.45–0.52)	0.48 (0.45–0.51)	0.001 (-0.014-0.015)
Months with home care services before hospitalization (mean)	8.58 (8.54–8.62)	0.68 (0.65–0.71)	1.06 (1-1.11)	1.18 (1.12–1.25)	-0.024 (-0.038-0.009)
Number of unplanned visits in specialized outpatient care last 12 months (mean)	2.53 (2.52–2.54)	1.63 (1.61–1.65)	1.16 (1.14–1.18)	1.24 (1.22–1.27)	-0.035 (-0.05-0.021)
Number of planned visits in specialized outpatient care last 12 months (mean)	2.9 (2.87–2.92)	1.53 (1.48–1.59)	1.39 (1.33–1.44)	1.44 (1.39–1.51)	-0.01 (-0.025-0.005)

Descriptive statistics for the study sample presented as discharges to private residence with HC and discharges to NH before (raw) and after propensity score matching (matched)

*No difference as exact matching was based on sex

### Outcomes

**Fig. 3:**
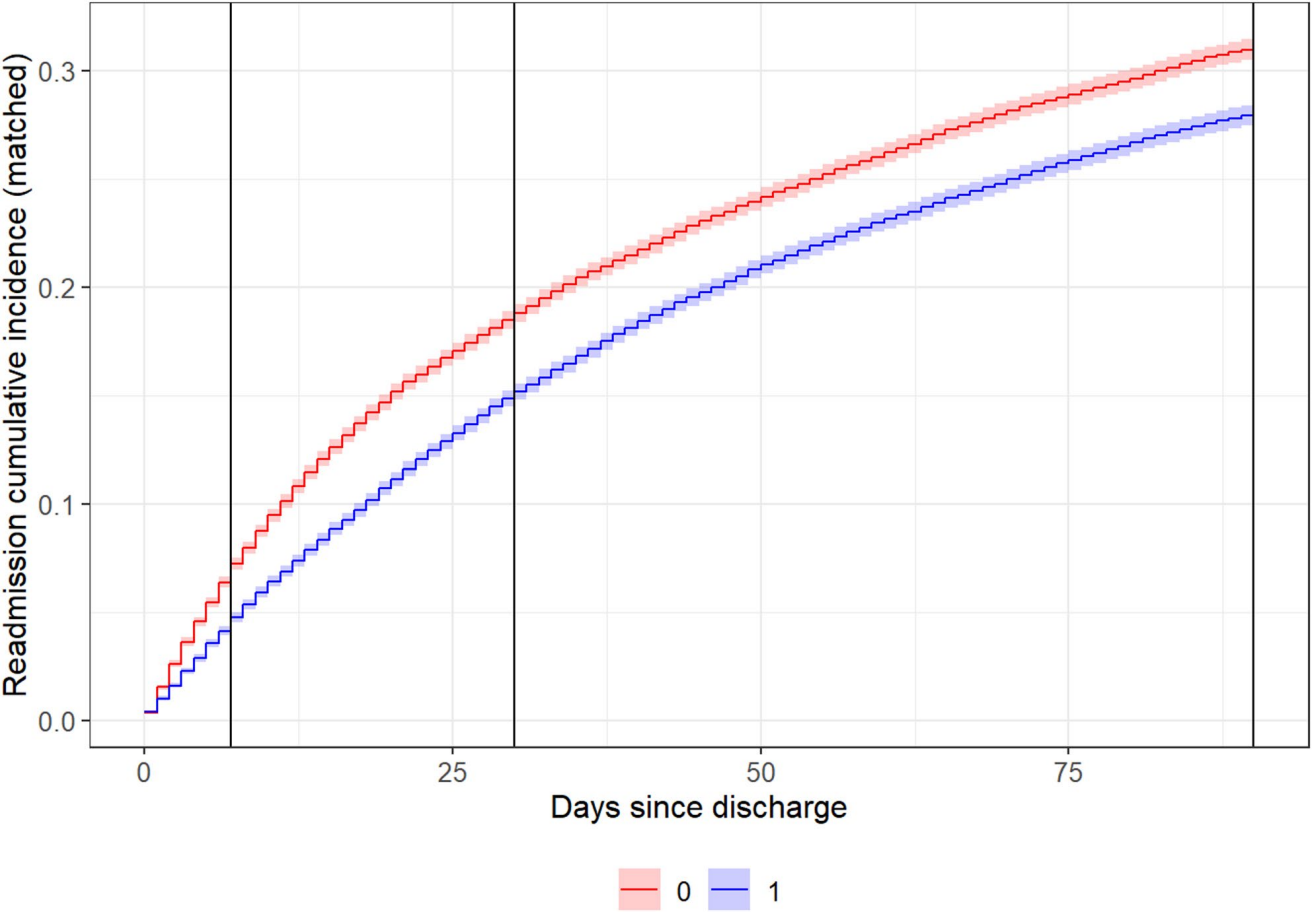
Cumulative incidence rate curve for readmissions in the matched study sample. Blue line (1) represents discharges where patients were admitted to nursing home after hospital discharge. Red line (0) represents discharges where patients received home care after hospital discharge

**Fig. 4:**
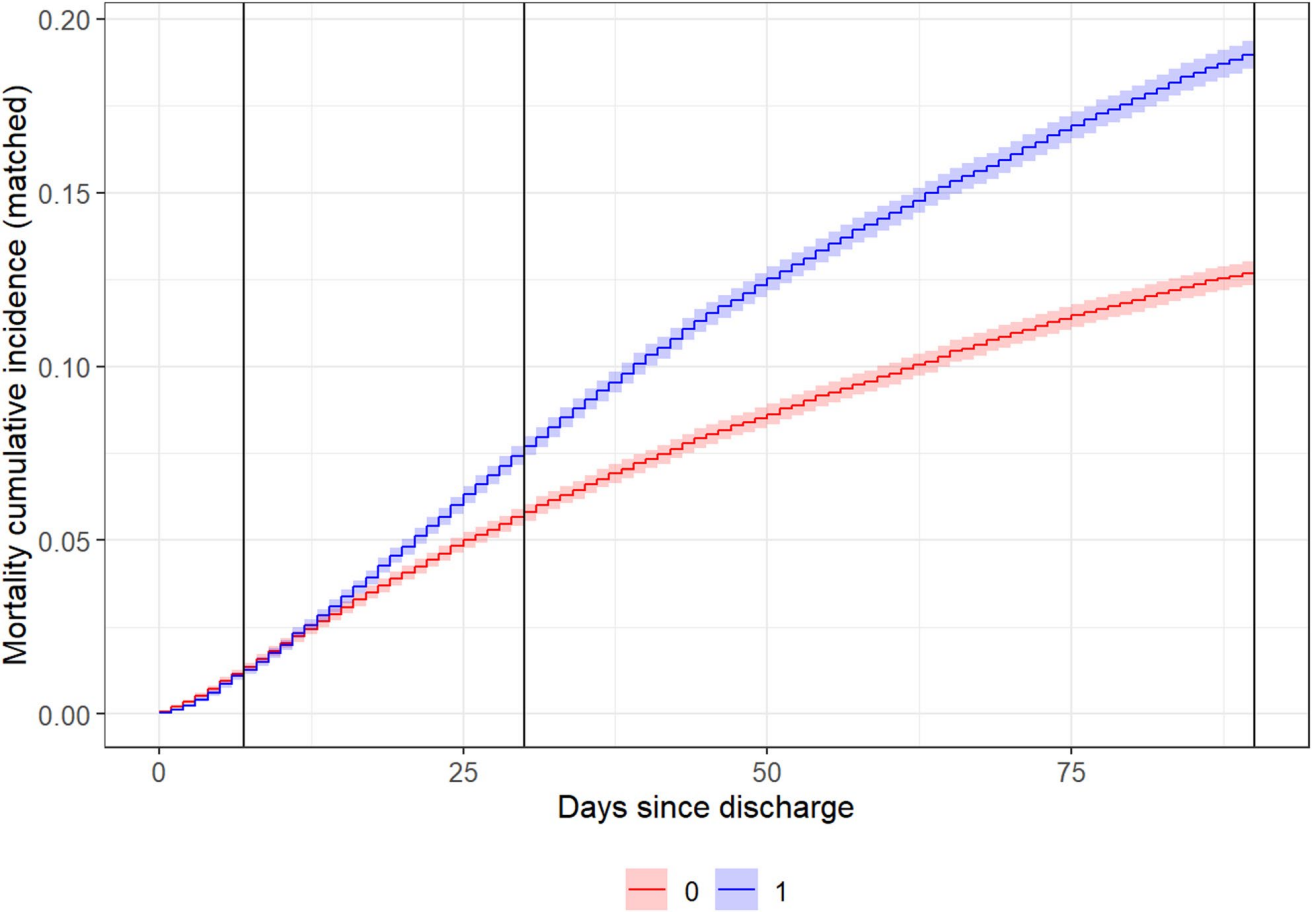
Cumulative incidence rate curve for mortality in the matched study sample. Blue line (1) represents discharges where patients were admitted to nursing home after hospital discharge. Red line (0) represents discharges where patients received home care after hospital discharge

Readmission and mortality rates were visualized using cumulative incident rate curves, see Fig. 3 and Fig. 4. Upon examination, differential effects were identified across various follow-up periods indicating that the proportional hazards assumption over the full follow-up period was violated. As such, the overall estimate of proportional hazards across the full 90-day follow-up period is inappropriate as a sole measure of outcomes. 

Over the first 7 days, discharges to NH had a sub distribution hazard rate (sHR) of 0.643 (95% CI 0.605–0.682) for hospital readmission and a Hazard Rate (HR) of 0.917 (0.806–1.044) for mortality, see Table 3. This suggests that NHs had a protective short-term effect regarding hospital readmission, though no significant difference was identified regarding mortality. Over 30 days, discharges to NH continued to have a lower hazard of being readmitted (sHR 0.765 (0.736–0.796)) but mortality was higher among discharges to nursing homes HR 1.319 (1.244–1.398). At 90 days post-discharge, the hazard ratio for readmissions increased to 0.846 (0.823–0.868), and mortality increased to 1.538 (1.481–1.598). Qualitatively, it may be seen in Fig. 3 that the difference in readmission rates between the groups stem primarily from short term rate differences, with readmission rates being largely equal after circa two weeks. As seen in Table 3, hazards were similar between unadjusted and adjusted (double robust) outcome models, suggestive of low confounding due to residual covariate imbalance. 

**Table 3:** Effects of being admitted to a NH post-discharge on readmissions and mortality in the matched study sample

Follow-up period	Readmission	Readmission (double robust)	Mortality	Mortality (double robust)
7-day	0.649 (0.612–0.689)	0.643 (0.605–0.682)	0.928 (0.817–1.053)	0.917 (0.806–1.044)
30-day	0.784 (0.754–0.816)	0.765 (0.736–0.796)	1.336 (1.263–1.413)	1.319 (1.244–1.398)
90-day	0.871 (0.847–0.896)	0.846 (0.823–0.868)	1.542 (1.487–1.599)	1.538 (1.481–1.598)

Results from the survival analysis for readmissions (competing risk regressions) and mortality (cox proportional hazards) reported at 7-, 30- and 90-days post-discharge. Readmission rates are reported as sub-distribution hazard ratios and mortality rates are reported as hazard ratios. 95% confidence intervals in parenthesis

### Sensitivity analyses

Four sensitivity analyses were performed as part of the analysis. The first evaluated the use of a more traditional parametric model suitable for the large number of predictors employed (a regularized generalized additive model implemented using the R package *glmnet*), see supplementary materials 2. This resulted in reduced performance of the matching process (Pseudo r2 0.42) and considerably degraded covariate balance but substantively similar results (30-day mortality HR 1.264 (1.204–1.328) and 30-day readmission HR 0.819 (0.793–0.846)). An analysis was also performed assessing results without the data quality related exclusion criteria, see supplementary materials 3. This primarily impacted the NH group, which was found to have generally higher mortality and readmission rates, and subsequently also higher relative adjusted rates (30-day mortality HR 1.987 (1.917–2.06) and 30-day readmissions 0.89 (0.868–0.912)). These results suggest that there may be selection effects at play, with sicker patients not being appropriately registered as receiving NH care in at least one of the national registries used in this study. Third, an analysis was performed explicitly including only the first of each patient’s hospital admissions during the study period, rather than excluding repeat admissions in the matching process, see supplementary materials 4. This analysis identified stable results regarding readmission (30-day HR 0.732 (0.707–0.757)), but mortality rates differed from the main analysis (30-day HR 1.979 (1.842–2.126)), suggesting that these findings may be sensitive to choices made in the analytical process. Finally, a composite outcome of readmission or mortality was analyzed similarly to the main results, see supplementary materials 1. The effects regarding this composite outcome were substantially smaller compared to when readmissions and mortality were analyzed separately for the longer-term outcomes (30-day HR 0.94 (0.911–0.97) and 90-day HR 1.05 (1.025–1.075)). However, the protective effect of NHs was still identified at 7 days post-discharge (HR 0.69 (0.657–0.735)).

## Discussion

This study utilized health- and social care data from the entire Swedish population in 2015–2019 in order to emulate a randomization between being discharged to NH or HC. As seen in Table 2, the matching process on observed covariates was successful, with good covariate balance between hospital episodes discharged to NH and HC. The results show that being discharged to a NH appears to have protective effects in the short-term regarding readmissions, with no excess mortality during the first week after hospital discharge. Over longer time periods readmissions among NH discharges remained lower, while mortality was elevated at both 30- and 90-days post discharge.

It is important to note that our study employs an ATT estimand on a restricted cohort of hospital discharges where patients are particularly susceptible to random variation in treatment assignment. As such, the results are not directly comparable to previous studies and should not be generalized to the entire population of patients discharged to NH or HC. Nevertheless, this study identified similar results as previous studies using other methodologies. For instance, the findings of decreased readmissions at 30 days post discharge for patients discharged to NHs is in line with Werner et al., looking at 17 million patients in the Medicare system [21]. This result may be due to more intense supervision and timely medical attention in NHs, and thus a lower risk of delayed medical care during the post-discharge period. From the patient’s point of view, being transferred from hospital directly to the home setting may be a jarring experience, and the use of NH facilities as an intermediate step prior to discharge home may allow better acclimatization to self-care and closer observation during the high-risk post-discharge phase. Our results also align with the increased mortality of NH patients reported by Pfaff et al. [20]. It is worth noting however that the study by Pfaff et al. was was limited to three inpatient geriatric wards which may limit its generalizability.

The short-term protective effects of NH care identified in this study suggest that the expanded use of NH care particularly during the first week following discharge may be an effective approach to reducing post-discharge readmissions. Regarding longer-term outcomes, our results indicate that readmissions remained lower during the full 90-day follow-up period for discharges to NHs. This is in line with the study by Seaman et al., which showed that hospital admissions decreased in the year after admission to NHs in Australia [22].

The relatively higher longer-term mortality and inverse relationships between readmission and mortality rates observed among discharges to NHs in this study are noteworthy and may have several possible explanations that warrant further investigation. We suggest two potential interpretations regarding the findings of higher mortality at nursing homes. First, the results may be due to differences in how end-of-life care is handled at NHs vs. in HC. It is possible that NHs make more extensive use of advanced care plans, thus reducing hospital transfers in the final stages of life. Second, the results may reflect the decline in health that has been associated with NH admissions [12, 13]. Although speculative, we cannot exclude that the results regarding mortality reflect unmet medical needs in patients living in NHs and that the relatively higher readmissions observed in the HC-group are life-prolonging.

In summary, the results indicate that there exists a cohort of older, multimorbid patients for whom there is ambiguity in deciding whether discharge to NH or HC is the most appropriate form of care. These decisions can furthermore have significant impacts on outcomes among these patients. This warrants further research to identify the most appropriate discharge processes and follow-up care, and to determine the extent to which expanding the number of NH-beds is required to meet the needs of aging populations.

### Limitations

The primary limitation of this study is that the registry data used does not include direct measurements of frailty, cognitive status, functional capacity, or informal care. While some proxy measures of these factors were included (e.g. the number of months with HC and home health care prior to hospital admission and marital status), the results may be influenced by unmeasured confounding given the lack of variables directly measuring these dimensions. Frailty for instance is likely to be greater in the NH-group and adjusting/matching for a direct measurement of frailty would likely decrease the HR for mortality in comparison to HC. Regarding readmissions, the effect of potential residual confounding is less clear. Intuitively, adjusting for unmeasured confounders would be likely to decrease the HR for readmission in comparison to HC in a similar manner as with mortality. However, very frail patients near the end of life may also be less likely to be readmitted in NHs given the strong focus in this setting on providing end-of-life/palliative care [37]. In sensitivity analyses, effect estimates tended to vary more widely regarding mortality, while the protective effect of NH care on readmissions was robust across all analyses and timepoints.

It should furthermore be noted that the patient cohort investigated here consists of a somewhat narrow selection of discharges where the assumption of positivity is plausibly fulfilled, i.e., where there is some chance of assignment to either NH or HC upon discharge. Similarly, discharges to NH per the in-patient care registry where no corresponding NH care was registered in the municipal health care registry were excluded in the main analysis. These patients, however, had notably higher rates of negative outcomes, indicating possible selection effects which should be considered when interpreting the findings. As such, the effects identified here should not be generalized to the full population of NH and HC patients, but considered in the context of clinical decisions regarding patients potentially relevant for placement in either form of care.

## Conclusion

Patients discharged to NH had lower rates of readmission and similar rates of mortality within 7 days as patients discharged to their private residence supported by HC. Over longer time frames, readmission rates were more similar, while mortality rates were higher at nursing homes. The findings suggest that post-discharge NH care may be effective in reducing short-term readmissions, while longer-term impacts were smaller.

## Supplementary Information

Supplementary Material 1.

Supplementary Material 2.

Supplementary Material 3.

Supplementary Material 4.

## Data Availability

Individual level patient data cannot be made publicaly available as the Swedish Ethical Review Act state that these types of sensitive data can only be made available to researchers who meet the criteria for access after legal review by the Ethical Review Authority of Sweden. All data are available from the National Board of Health and Welfare registry unit.

## Abbreviations

HC: Home Care
NH: Nursing Home
