# Supplementary material for: Estimating the impact of discharge to nursing home on readmission and mortality: a propensity score matched analysis
Source: BMC Geriatr. 2026 Jul 4;26:909. doi: 10.1186/s12877-026-07761-8 (PMC13339853; doi:10.1186/s12877-026-07761-8)
Supplement: Supplementary file 2 — Supplementary Material 2. [file 12877_2026_7761_MOESM2_ESM.html]

The causal impact of discharge to nursing home on readmission and mortality: A propensity score matched survival analysis


# The causal impact of discharge to nursing home on readmission and mortality: A propensity score matched survival analysis

- Setup
- Load data
- Train propensity models
- Perform
  matching
- Propensity score validation
  - mean values by percentile
- Pseudo
  r-squared
- Generate outcome
  predictions
- Matching
  table
- Categorical predictor
  distributions
  - Major Diagnostic Group
  - Hospital
    Ward
  - Region
- Propensity
  score distribution in raw and matched samples
- Propensity score variable
  description
  - Variable
    summary
  - Detailed variable table
  - Partial dependence plot
- SMD for all predictors
- Main analysis
  - Estimate
    models
  - Plot cumulative incidence
    curves
  - Hazard
    ratio table

# Setup

```
knitr::opts_chunk$set(cache = FALSE, 
                      warning = FALSE, 
                      message = FALSE, 
                      cache.lazy = FALSE)

options(scipen = 999,cache.lazy = FALSE)

set.seed(42)

# Set flag to regenerate stored artifacts
reload = F

# set to "glm_propensity" for sensitivity analysis concerning effect of alternate propensity score model
# set to "noqualityexcl" for sensitivity analysis concerning effect without excluding discharges at risk for documentation errors
# set to "first_patient" for sensitivity analysis concering effect of including only the first contact for each patient (instead of excluding repeat patients at the matching stage)
sens_analysis <- "glm_propensity"

path = paste0(getwd(),"/",sens_analysis)
dir.create(path)
```

```
## Warning in dir.create(path): 'C:\Users\dousp651\Projects\SIP -
## Documents\nh_impact\glm_propensity' already exists
```

```
knitr::opts_knit$set(root.dir = path)

suppressPackageStartupMessages({

  library(tidycmprsk)
  library(ggsurvfit)
  library(readxl)
  library(lubridate)
  library(boot)
  library(xgboost)
  library(data.table)
  library(knitr)
  library(tidyverse)
  library(MatchIt)
  library(marginaleffects)
  library(survival)
  library(survminer)
  library(Matrix)
  library(cmprsk)
  library(glmnet)
  library(effectsize)
  library(DT)
})
```

```
# Define MDC codes based on NBHW definitions https://www.socialstyrelsen.se/statistik-och-data/klassifikationer-och-koder/drg/drg-koder-och-definitioner/


mdc = c("A" = "Nervous System", 
        "B" = "Eye", 
        "C" = "Ear, Nose, Mouth, And Throat", 
        "D" = "Respiratory System", 
        "E" = "Circulatory System", 
        "F" = "Digestive System", 
        "G" = "Hepatobiliary System and Pancreas", 
        "H" = "Musculoskeletal System \n and Connective Tissue", 
        "J" = "Skin, Subcutaneous \nTissue, and Breast", 
        "K" = "Mammary gland diseases", 
        "L" = "Endocrine, Nutritional,\n and Metabolic System", 
        "M" = "Kidney and Urinary Tract", 
        "N" = "Male Reproductive System", 
        "O" = "Female Reproductive System", 
        "P" = "Pregnancy, Childbirth, and Puerperium", 
        "Q" = "Newborn and Other Neonates (Perinatal Period)",
        "R" = "Blood/-Forming and Immun./\nMyeloprolif. Dis. / NS tumors", 
        "S" = "Infectious and Parasitic\n Diseases and Disorders", 
        "T" = "Mental Diseases and Disorders", 
        "U" = "Injuries, Poison, \nand Toxic Effect of Drugs", 
        "V" = "Burns", 
        "W" = "Factors Influencing Health Status", 
        "Z" = "Ungroupable")

mdc_df <- data.frame(mdc = names(mdc),
                     mdc_name = mdc)

## Set parameters

  # Maximum length of stay to include
  max_caredays = 90
  
  # Minimum number of observations from a hospital ward to include
  min_hosp_mvo_obs = 100 
  
  # Time span to search forwards from a contact to identify readmissions
  fu_days = 90
  
  # Time span to search backwards from a contact to identify previous admissions
  prev_days = 365

# Function for getting bootstrap CIs for mean
mean_fun <- function(data,inds){
  return(mean(data[inds]))
}

# Function for calculating number of contacts within a given timeframe
window_dates <- function(d,s,i){
  d = unlist(d)
  l = length(d[d >= s & d <= i])
  return(l)
}


elapsed_months <- function(end_date, start_date) {

  
  sd <- as.POSIXlt(start_date)
  ed <- as.POSIXlt(end_date)
  
  return(12 * (ed$year - sd$year) + (ed$mon - sd$mon))
}

# Function for calculating the quantile of a value

get_q <- function(x,q){sum(x>unlist(q))/(length(unlist(q)))}

# Function for generating sparse matrix for propensity xgboost model

generate_sparsm <- function(data){
  
  cats <- data %>%
    ungroup() %>%
    dplyr::select(id,
           mvo,
           weekday,
           op = interventions,
           prevdiag = prev_last_diag,
           diagprim = diag_prim_last,
           diagsec = diag_sec,
           region,
           muni,
           hosp,
           born,
           civil,
           admit) %>% 
    mutate(mvo = gsub(" ","|",mvo),
           diagprim = gsub(" ","|",diagprim),
           diagsec = gsub(" ","|",diagsec),
           op = gsub(" ","|",op),
           prevdiag = gsub(" ","|",prevdiag),
           muni = gsub(" ","|",muni)
           ) %>%
    mutate(across(everything(),function(x) strsplit(as.character(x),"\\|"))) %>%
    pivot_longer(-id) %>%
    unnest(cols = value,keep_empty = T) %>%
    mutate(id = as.numeric(id),
           name = make.names(paste(name,value,sep = "_")),
           value = 1) %>%
    distinct() %>%
    ungroup()
  
  ids <- unique(cats$id)
  names <- unique(cats$name)
  
  # Map to sparse row/column indices
  id_map <- data.frame(sm_id = seq(1,length(ids)),
                       id = ids)
  
  name_map <- data.frame(sm_d = seq(1,length(names)),
                         name = names)
  
  # Join to data
  cats <- cats %>%
    left_join(id_map) %>%
    left_join(name_map)
  
  
  sprsM_cat <- as(sparseMatrix(i = cats$sm_id,
                               j = cats$sm_d,
                               x = cats$value,
                               dimnames = list(unique(cats$id)[order(unique(cats$sm_id))],
                                               unique(cats$name)[order(unique(cats$sm_d))])), "dgCMatrix")
  
  
  num <- data %>%
    transmute(date = as.numeric(out_date),
              week,
              caredays = caredays,
              prevSince = ifelse(is.na(prev_caredays),fu_days+1,prev_caredays),
              prevCaredays = ifelse(is.na(days_since_prev),366,days_since_prev),
              planned = planned_contact,
              age,
              countHomecareMonths = n_hc,
              countHomeserviceMonths = n_htj,
              countAmbPlanned = count_yr_ov_planned,
              countAmbUnplanned = count_yr_ov_unplanned,
              countDiags = n_diag,
              countInterventions = n_op,
              female = gender,
              countYr = count_yr,
              countYrUnplanned = count_yr_unplanned) 
  
  
  sprsM <- num %>%
    as.matrix() %>%
    Matrix(sparse = T) %>%
    cbind(sprsM_cat)
  
  return(sprsM)
  
}
```

# Load data

Data = hospital data Sol = SOL data

```
# Load data 

if(file.exists("./data_final.rda") & 
   file.exists("./excl_n.rda") & !reload){
load("data_final.rda")
load("excl_n.rda")
}else{
  
  parent_dir <- dirname(getwd())
  load(paste0(parent_dir,"/data.rda"))
  load(paste0(parent_dir,"/sol.rda"))
  
  d$disch_sabo <- as.numeric(d$discharge == "Särskilt boende")
  
#df indicating which months patient has NH or short term stay 
sabo <- sol %>%
  filter(BOFORM == 2 | KORTTID == 1) %>%
  dplyr::select(lopnr, date_month,shortterm = KORTTID) %>%
  distinct()

#calculate dates for NH at discharge
sabo_dates <- d %>%
  dplyr::select(lopnr,grp, in_date,out_date,admit,discharge) %>%
  left_join(sabo) %>%
  filter(date_month >= out_date - months(3)) %>%
  group_by(lopnr,grp) %>%
  filter(date_month == min(date_month)) %>%
  ungroup() %>%
  distinct() %>%
  mutate(sabo_diff = date_month - out_date)

#calculate dates for NH at admission
sabo_dates_in <- d %>%
  dplyr::select(lopnr,grp, in_date,out_date,admit,discharge) %>%
  left_join(sabo) %>%
  filter(date_month >= in_date - months(3)) %>%
  group_by(lopnr,grp) %>%
  filter(date_month == min(date_month)) %>%
  ungroup() %>%
  distinct() %>%
  mutate(sabo_diff_in = date_month - in_date)

# Get home service / home care dates
htj <- sol %>%
  filter(BOFORM <= 1 & KORTTID != 1,
         HTJ == 1) %>%
  dplyr::select(lopnr, date_month) %>%
  distinct()

hsl <- sol %>%
  filter(BOFORM <= 1 & KORTTID != 1,
         HSL == 1) %>%
  dplyr::select(lopnr, date_month) %>%
  distinct()
  
htj_dates <- d %>%
  dplyr::select(lopnr,grp, in_date,out_date,admit,discharge) %>%
  left_join(htj) %>%
  filter(date_month >= out_date - months(3)) %>%
  group_by(lopnr,grp) %>%
  filter(date_month == min(date_month)) %>%
  ungroup() %>%
  distinct() %>%
  mutate(htj_diff = date_month - out_date)

hsl_dates <- d %>%
  dplyr::select(lopnr,grp, in_date,out_date,admit,discharge) %>%
  left_join(hsl) %>%
  filter(date_month >= out_date - months(3)) %>%
  group_by(lopnr,grp) %>%
  filter(date_month == min(date_month)) %>%
  ungroup() %>%
  distinct() %>%
  mutate(hsl_diff = date_month - out_date)

d <- d %>%
  left_join(dplyr::select(sabo_dates,lopnr,grp,
                          sabo_date = date_month,
                          sabo_diff,shortterm),
            by=c("lopnr","grp")) %>%
  left_join(dplyr::select(sabo_dates_in,lopnr,grp,
                          sabo_date_in = date_month,
                          sabo_diff_in),
            by=c("lopnr","grp")) %>%
  left_join(dplyr::select(htj_dates,lopnr,grp,
                          htj_date = date_month,
                          htj_diff),
            by=c("lopnr","grp")) %>%
  left_join(dplyr::select(hsl_dates,lopnr,grp,
                          hsl_date = date_month,
                          hsl_diff),
            by=c("lopnr","grp"))


d <- d %>%
  #Exclude if admitted from home but has had NH care registered for at least 2 months before discharge (these patients are likely living in NH already)
  mutate(excl_admit = !(admit == "Ordinärt boende" & sabo_diff_in < -30 ) | is.na(sabo_diff),
  #Exclude if discharged to NH but no record of NH care for at least 2 months after discharge
  excl_discharge_sabo = !(discharge == "Särskilt boende" & (sabo_diff > 30 | is.na(sabo_diff))),
  #Exclude if discharged to home but record of NH care within 2 months
  excl_discharge_home = !(discharge == "Ordinärt boende" & (sabo_diff < 30 & !is.na(sabo_diff))),
  shortterm = ifelse(is.na(shortterm),0,shortterm)) %>%
  ungroup() %>%
  select(-dates,-unplanned_dates)

  


# Apply exclusion criteria, and save the number of included records at each step.
excl_n <- list()

excl_n$orig <- nrow(d)

#Exclude cases prior to 2016 (2015 used to calculate propensity score data)
d <- d %>%
 filter(year(out_date) > 2015)

excl_n$year_2015 <- nrow(d)

d <- d %>%
  filter(planned_contact == 0)

excl_n$unplanned <- nrow(d)

d <- d %>%
  arrange(lopnr,grp) %>%
  mutate(days_to_next_unplanned = ifelse(lead(lopnr) == lopnr,lead(in_date) - out_date,NA))

d <- d %>%
 filter(!is.na(source_discharge),
        !is.na(source_admit),
        !is.na(last_hosp))

excl_n$missingdata <- nrow(d)

d <- d %>%
  filter(!source_discharge %in% c(4,1))

excl_n$discharge <- nrow(d)

d <- d %>%
  filter(caredays > 2)

excl_n$shortstay <- nrow(d)

d <- d %>%
  filter(caredays <= max_caredays)

excl_n$longstay <- nrow(d)

d <- d %>%
  filter(out_date < ymd("20200101") - days(fu_days))

excl_n$endofstudy <- nrow(d)

d <- d %>%
  filter(!is.na(last_hosp_mvo))

excl_n$missingmvo <- nrow(d)

d <- d %>%
  filter((days_since_prev >= 0 | is.na(days_since_prev)) & 
           (out_days_to_next >= 0 | is.na(out_days_to_next)))

excl_n$shortgap <- nrow(d)

d <- d %>%
  filter(source_admit == 3)

excl_n$admitfromhome <- nrow(d)

if(sens_analysis != "noqualityexcl"){
  d <- d %>%
    filter(excl_admit & excl_discharge_sabo & excl_discharge_home)
}


excl_n$uncertain_admit_discharge <- nrow(d)

d <- d %>%
  filter((!is.na(htj_diff) & htj_diff < 30) | disch_sabo == 1)

excl_n$admit_home_no_care <- nrow(d)

d <- d %>%
  filter(n_diag > 1)

excl_n$multi_diag <- nrow(d)

if(sens_analysis != "noqualityexcl"){
  d <- d %>%
    group_by(lopnr) %>%
    mutate(first_disch_nh = min(out_date[disch_sabo == 1 & shortterm == 0],na.rm = T)) %>%
    ungroup() %>%
    filter(in_date < first_disch_nh)
}

excl_n$admitfromhome_after_nhdisch  <- nrow(d)

if(sens_analysis == "first_patient"){
  d <- d %>%
    group_by(lopnr) %>%
    filter(grp == min(grp))

}


excl_n$final <- nrow(d)

# Append home care volume data

htj_sum <- htj %>%
     arrange(lopnr, date_month) %>%
     group_by(lopnr) %>%
     mutate(n_htj = row_number()-1) %>%
  ungroup( )%>%
  select(lopnr, discharge_date_month = date_month, n_htj) %>%
  filter(lopnr %in% d$lopnr)

hsl_sum <- hsl %>%
     arrange(lopnr, date_month) %>%
     group_by(lopnr) %>%
     mutate(n_hc = row_number()-1) %>%
  ungroup() %>%
  select(lopnr, discharge_date_month =  date_month, n_hc) %>%
  filter(lopnr %in% d$lopnr)

muni_sums <- full_join(htj_sum,hsl_sum)

d <- d %>%
     left_join(muni_sums,
               by = c("lopnr","discharge_date_month")) %>%
  ungroup()

d$n_htj[is.na(d$n_htj)] <- 0
d$n_hc[is.na(d$n_hc)] <- 0

  save(d, file = "data_final.rda")
  save(excl_n, file = "excl_n.rda")
}
```

```
length(unique(d$lopnr))
```

```
## [1] 230815
```

```
excl_n_per_stage <- as.data.frame(t(as.data.frame(excl_n))) %>%
  transmute(sum = V1,
            excl = lag(sum)-sum,
            description = c("Patients over 65 with 2+ ICD codes", 
                            "Year > 2015", 
                            "Unplanned admission", 
                            "Missing discharge/admission/hospital data", 
                            "Discharge to home or NH", 
                            "Care duration < 3 days", 
                            "Care duration > 90 days", 
                            "Year < 2020", 
                            "Missing ward information", 
                            "Same day readmission", 
                            "Patient admitted from home", 
                            "Agreeement re: admission and discharge between data sources", 
                            "Discharged home without care services",
                            "Single diagnosis only",
                            "Admission from home discharged to NH following discharge to NH",
                            "Final"))


kable(excl_n_per_stage,row.names = F)
```

| sum | excl | description |
| --- | --- | --- |
| 2852592 | NA | Patients over 65 with 2+ ICD codes |
| 2289148 | 563444 | Year > 2015 |
| 1856728 | 432420 | Unplanned admission |
| 1856278 | 450 | Missing discharge/admission/hospital data |
| 1704954 | 151324 | Discharge to home or NH |
| 1376882 | 328072 | Care duration < 3 days |
| 1375519 | 1363 | Care duration > 90 days |
| 1288843 | 86676 | Year < 2020 |
| 1287612 | 1231 | Missing ward information |
| 1287108 | 504 | Same day readmission |
| 1188724 | 98384 | Patient admitted from home |
| 1001640 | 187084 | Agreeement re: admission and discharge between data sources |
| 434405 | 567235 | Discharged home without care services |
| 413657 | 20748 | Single diagnosis only |
| 413073 | 584 | Admission from home discharged to NH following discharge to NH |
| 413073 | 0 | Final |

```
# Final n:
nrow(d)
```

```
## [1] 413073
```

```
# Generate sparse matrix for xgb


if(file.exists("./sm.rda") & !reload){
  load("./sm.rda")
}else{

  sm <- generate_sparsm(d)
  
  save(sm,file = "./sm.rda")
  
}
```

# Train propensity models

```
if(file.exists("./propensity_xgb.rda") & !reload){
  load("./propensity_xgb.rda")
  load("./propensity_xgb_cv.rda")
  load("./propensity_glm.rda")
  load("./propensity_glm_cv.rda")
}else{

  params <- list(objective = "binary:logistic")


  propensity_xgb_cv <- xgb.cv(data = xgb.DMatrix(data  = sm,
                                                 label = as.logical(d$disch_sabo)),
                             nfold = 5,
                             nrounds = 500,
                             prediction = T, 
                             params = params,
                             early_stopping_rounds = 10)

  propensity_xgb <- xgb.train(data = xgb.DMatrix(data  = sm,
                                                 label = as.logical(d$disch_sabo)),
                              params = params,
                            nrounds = propensity_xgb_cv$early_stop$best_iteration)
  

  
  propensity_glm_cv <- cv.glmnet(x=sm,
                                 y = as.logical(d$disch_sabo))
  
  propensity_glm <- glmnet(x=sm,
                           y = as.logical(d$disch_sabo),
                           lambda = propensity_glm_cv$lambda.min)
  
  
  
  save(propensity_xgb_cv,file = "./propensity_xgb_cv.rda")
  save(propensity_xgb,file = "./propensity_xgb.rda")
  save(propensity_glm_cv,file = "./propensity_glm_cv.rda")
  save(propensity_glm,file = "./propensity_glm.rda")
  
}
```

```
d_eval <- d %>% 
  ungroup() %>%
  dplyr::select(id,
                lopnr,
                out_date,
                age,
                gender,
                region,
                muni,
                caredays,
                planned_contact,
                mdc,
                mvo_last,
                n_op,
                n_diag,
                disch_sabo,
                days_since_prev,
                count_yr,
                count_yr_unplanned,
                count_yr_ov_planned,
                count_yr_ov_unplanned,
                unplanreadmit30,
                mort30,
                days_to_next_unplanned,
                mort_days,
                sabo_diff,
                n_hc,
                n_htj) %>% #include months of HC before hospitalization 
  mutate(nh_propensity_xgb = propensity_xgb_cv$cv_predict$pred,
         nh_propensity_glm = predict(propensity_glm,newx = sm),
         nh_propensity = nh_propensity_xgb,
         mortorreadmit30 = pmax(unplanreadmit30,mort30),
         days_since_prev_fill = ifelse(is.na(days_since_prev),366,days_since_prev),
         prop_weight = disch_sabo/nh_propensity + (1-disch_sabo)/(1-nh_propensity),
         prop_strat = cut(nh_propensity,quantile(nh_propensity,probs = seq(0, 1, 0.1)),include.lowest = T),
         ts_days_to_next_unplanned = ifelse(days_to_next_unplanned>=0,days_to_next_unplanned,NA),
         ts_days_to_sabo = ifelse(sabo_diff>=0 & disch_sabo == 0,sabo_diff,NA),
         ts_days_to_mort = ifelse(mort_days>=0,mort_days,NA),
         ts_days_to_any = pmin(ts_days_to_next_unplanned,ts_days_to_mort,na.rm = T),
         
         ts_mort_days_pp = pmin(ts_days_to_mort,
                             ts_days_to_sabo,
                             fu_days,na.rm = T),
         ts_mort_event_pp = ts_days_to_mort == ts_mort_days_pp & !is.na(ts_days_to_mort),
         
         
         ts_mort_days_itt = pmin(ts_days_to_mort,
                             fu_days,na.rm = T),
         ts_mort_event_itt = ts_days_to_mort == ts_mort_days_itt & !is.na(ts_days_to_mort),
         
         ts_any_days_itt = pmin(ts_days_to_any,
                             fu_days,na.rm = T),
         
         ts_any_event_itt = ts_days_to_any == ts_any_days_itt & !is.na(ts_days_to_any),
         
         ts_readmit_days_pp = pmin(ts_days_to_next_unplanned,
                             ts_days_to_mort,
                             ts_days_to_sabo,
                             fu_days,na.rm = T),
         ts_readmit_event_pp = as.factor(ifelse(ts_readmit_days_pp == ts_days_to_next_unplanned & 
                                 !is.na(ts_days_to_next_unplanned), "Readmission",
                               ifelse(ts_readmit_days_pp == ts_days_to_mort & 
                                 !is.na(ts_days_to_mort), "Death","Censored"))),
         
         ts_readmit_days_itt = pmin(ts_days_to_next_unplanned,
                             ts_days_to_mort,
                             fu_days,na.rm = T),
         
         ts_readmit_event_itt = factor(ifelse(ts_readmit_days_itt == ts_days_to_next_unplanned & 
                                         !is.na(ts_days_to_next_unplanned), "Readmission",
                                       ifelse(ts_readmit_days_itt == ts_days_to_mort & 
                                         !is.na(ts_days_to_mort), "Death","Censored")),
                                       levels = c("Censored","Readmission","Death")),
         
         ts_readmit_days_itt_se = pmin(ts_days_to_next_unplanned,
                             ts_days_to_mort,
                             fu_days,na.rm = T),
         ts_readmit_event_itt_se = ts_readmit_days_itt == ts_days_to_next_unplanned &
           !is.na(ts_days_to_next_unplanned),
         ts_mort_days_itt_7 = pmin(ts_mort_days_itt, 7),
         ts_mort_event_itt_7 = ifelse(ts_mort_days_itt <= 7 & ts_mort_event_itt, T, F),
         
         ts_mort_days_itt_30 = pmin(ts_mort_days_itt, 30),
         ts_mort_event_itt_30 = ifelse(ts_mort_days_itt <= 30 & ts_mort_event_itt, T, F),
         
         ts_readmit_days_itt_7 = pmin(ts_readmit_days_itt, 7),
         ts_readmit_event_itt_7 = factor(ifelse(ts_readmit_days_itt <= 7, 
                                                 as.character(ts_readmit_event_itt),
                                                 "Censored"),levels = c("Censored",
                                                                        "Readmission",
                                                                        "Death")),
         
         ts_readmit_days_itt_30 = pmin(ts_readmit_days_itt, 30),
         ts_readmit_event_itt_30 = factor(ifelse(ts_readmit_days_itt <= 30, 
                                                 as.character(ts_readmit_event_itt),
                                                 "Censored"),levels = c("Censored",
                                                                        "Readmission",
                                                                        "Death")),
         ts_any_days_itt_7 = pmin(ts_any_days_itt, 7),
         ts_any_event_itt_7 = ifelse(ts_any_days_itt <= 7 & ts_any_event_itt, T, F),
         
         ts_any_days_itt_30 = pmin(ts_any_days_itt, 30),
         ts_any_event_itt_30 = ifelse(ts_any_days_itt <= 30 & ts_any_event_itt, T, F),
         )

if(sens_analysis == "glm_propensity") {
  d_eval$nh_propensity <- d_eval$nh_propensity_glm
}
```

# Perform matching

```
if(file.exists("./m_exact.rda") & !reload){
  #load("./m_full.rda")
  #load("./m_caliper.rda")
  load("./m_exact.rda")
}else{
  
  pct_99 = quantile(d_eval$nh_propensity[d_eval$disch_sabo==0],probs = 0.99)
  
  # Force matchit to use all NH discharges by preventing it from selecting NH patients as controls
  m_d_eval <- d_eval %>% 
    group_by(lopnr) %>%
    filter(disch_sabo == 1 | !(any(disch_sabo == 1) & disch_sabo == 0),
           !(nh_propensity > pct_99 & disch_sabo == 1)) # remove NH discharges above 99th percentile of non-NH discharged propensities to ensure even post matching propensity score distribution
    
  
  # m_ind_full <- matchit(disch_sabo ~ nh_propensity, 
  #              data = m_d_eval, 
  #              method="nearest",
  #              ratio=1,
  #              unit.id = "lopnr" #this argument ensures that only one episode is used 
  #              )
  # 
  # m_ind_caliper <- matchit(disch_sabo ~ nh_propensity, 
  #              data = m_d_eval, 
  #              method="nearest",
  #              caliper = 0.05,
  #              ratio=1,
  #              unit.id = "lopnr"
  #              )
  
  m_ind_exact <- matchit(disch_sabo ~ nh_propensity, 
               data = m_d_eval, 
               method="nearest",
               caliper = 0.05,
               exact = ~ gender + mdc,
               ratio=1,
               unit.id = "lopnr" #this argument ensures that only one episode is used 
               )
  
  # save(m_ind_full,file = "./m_full.rda")
  # save(m_ind_caliper,file = "./m_caliper.rda")
  save(m_ind_exact,file = "./m_exact.rda")
  
}

#m_data_full <- match.data(m_ind_full)
#m_data_caliper <- match.data(m_ind_caliper)
load("./m_exact.rda")
m_data <- match.data(m_ind_exact)
```

# Propensity score validation

## mean values by percentile

```
m_data <- m_data %>%
  ungroup() %>%
  mutate(prop_strat_match = cut(nh_propensity,
                                quantile(nh_propensity,
                                         probs = seq(0, 1, 0.1)),
                                include.lowest = T))

d_strat <- m_data %>%
  group_by(prop_strat_match,disch_sabo) %>%
  summarise(mean_age = mean(age),
            pct_female = mean(gender),
            mean_caredays = mean(caredays),
            mean_n_op = mean(n_op),
            mean_n_diag = mean(n_diag),
            mean_days_since_prev = mean(days_since_prev,na.rm=T),
            mean_count_ov_unplan = mean(count_yr_ov_unplanned),
            mean_count_yr = mean(count_yr),
            mean_count_yr_unplan = mean(count_yr_unplanned),
            pct_unplanreadmit30 = mean(unplanreadmit30),
            pct_mort30 = mean(mort30),
            mean_hc = mean(n_hc),
            mean_htj = mean(n_htj),
            n = n()) %>%
  pivot_longer(cols = -c(prop_strat_match,disch_sabo))

d_strat_grp <- m_data %>%
  group_by(prop_strat_match) %>%
  summarise(mean_age = mean(age),
            pct_female = mean(gender),
            mean_caredays = mean(caredays),
            mean_n_op = mean(n_op),
            mean_n_diag = mean(n_diag),
            mean_days_since_prev = mean(days_since_prev,na.rm=T),
            mean_count_ov_unplan = mean(count_yr_ov_unplanned),
            mean_count_yr = mean(count_yr),
            mean_count_yr_unplan = mean(count_yr_unplanned),
            mean_dischsabo = mean(disch_sabo),
            n_dischsabo = sum(disch_sabo),
            pct_unplanreadmit30 = mean(unplanreadmit30),
            pct_mort30 = mean(mort30),
            mean_hc = mean(n_hc),
            mean_htj = mean(n_htj),
            n = n()) %>%
  pivot_longer(cols = -c(prop_strat_match))

d_props <- m_data %>%
  group_by(disch_sabo) %>%
  summarise(mean_age = mean(age),
            pct_female = mean(gender),
            mean_caredays = mean(caredays),
            mean_n_op = mean(n_op),
            mean_n_diag = mean(n_diag),
            mean_days_since_prev = mean(days_since_prev,na.rm=T),
            mean_count_ov_unplan = mean(count_yr_ov_unplanned),
            mean_count_yr = mean(count_yr),
            mean_count_yr_unplan = mean(count_yr_unplanned),
            pct_unplanreadmit30 = mean(unplanreadmit30),
            pct_mort30 = mean(mort30),
            mean_hc = mean(n_hc),
            mean_htj = mean(n_htj),
            n = n()) %>% 
  pivot_longer(cols = -disch_sabo) %>%
  mutate(prop_strat = "Overall") %>%
  bind_rows(d_strat) %>%
  mutate(prop_strat = factor(prop_strat_match,levels = c("Overall",levels(d_strat$prop_strat_match))),
         prop_strat_n = as.numeric(prop_strat_match)-1,
         discharge = ifelse(disch_sabo == 1,"NH","Home"))


d_props %>%
  #filter(!name %in% c("pct_mort30","pct_unplanreadmit30")) %>% 
ggplot(aes(x = prop_strat_n,y=value,color=discharge))+
  geom_point() +
  scale_x_continuous(breaks = seq(0,10,1)) +
  facet_wrap(~name,scales = "free",ncol = 2) +
  theme(axis.text.x = element_text(angle = 90))
```

# Pseudo r-squared

```
Pseudo.R2=function(object){
  stopifnot(object$family$family == "binomial")
  object0 = update(object, ~ 1)
  wt <- object$prior.weights # length(wt)
      y = object$y # weighted
  ones = round(y*wt)
  zeros = wt-ones
  fv <- object$fitted.values   # length(fv)
      if (is.null(object$na.action)) fv0 <- object0$fitted.values else
        fv0 <- object0$fitted.values[-object$na.action] # object may have missing values
  resp <- cbind(ones, zeros)
  Y <- apply(resp, 1, function(x) {c(rep(1, x[1]), rep(0, x[2]))} )
  if (is.list(Y)) Y <- unlist(Y) else Y <- c(Y)
  # length(Y); sum(Y)
  fv.exp <- c(apply(cbind(fv, wt), 1, function(x) rep(x[1], x[2])))
  if (is.list(fv.exp)) fv.exp <- unlist(fv.exp) else fv.exp <- c(fv.exp)
  # length(fv.exp)
  fv0.exp <- c(apply(cbind(fv0, wt), 1, function(x) rep(x[1], x[2])))
  if (is.list(fv0.exp)) fv0.exp <- unlist(fv0.exp) else fv0.exp <- c(fv0.exp)
  (ll = sum(log(dbinom(x=Y,size=1,prob=fv.exp))))
  (ll0 = sum(log(dbinom(x=Y,size=1,prob=fv0.exp))))

  n <- length(Y)
  G2 <- -2 * (ll0 - ll)
  McFadden.R2 <- 1 - ll/ll0
  CoxSnell.R2 <- 1 - exp((2 * (ll0 - ll))/n) # Cox & Snell / Maximum likelihood pseudo r-squared
  r2ML.max <- 1 - exp(ll0 * 2/n)
  Nagelkerke.R2 <- CoxSnell.R2/r2ML.max  # Nagelkerke / Cragg & Uhler's pseudo r-squared

  out <- c(llh = ll, llhNull = ll0, G2 = G2, McFadden = McFadden.R2,
           r2ML = CoxSnell.R2, r2CU = Nagelkerke.R2)
  out
}

  disch_test <- glm(disch_sabo ~ nh_propensity, family = "binomial",
                               data = d_eval)
  
  Pseudo.R2(disch_test)
```

```
##             llh         llhNull              G2        McFadden            r2ML 
## -127907.0021289 -187786.2582188  119758.5121798       0.3188692       0.2516773 
##            r2CU 
##       0.4214570
```

# Generate outcome predictions

```
m_d_risk <- m_data %>%
  left_join(select(d,-names(d)[names(d) %in% names(m_data)],id))


if(file.exists("./m_sm.rda") & !reload){
  load("./m_sm.rda")
}else{

  m_sm <- generate_sparsm(m_d_risk)
  
  save(m_sm,file = "./m_sm.rda")
  
}

if(file.exists("./outcome_mod.rda") & !reload){
  load("./outcome_mod.rda")
}else{
  
  outcome_mod <- list()
  
  params = list(objective = "binary:logistic")
  outcome_mod$mort7_xgb_cv <- xgb.cv(data = xgb.DMatrix(data  = m_sm,
                                            label = m_d_risk$ts_mort_event_itt_7),
                         nrounds = 500,
                         nfold = 5,
                         prediction = T, 
                         early_stopping_rounds = 10,
                         params = params)
  
  outcome_mod$mort30_xgb_cv <- xgb.cv(data = xgb.DMatrix(data  = m_sm,
                                             label = m_d_risk$ts_mort_event_itt_30),
                         nrounds = 500,
                         nfold = 5,
                         prediction = T, 
                         early_stopping_rounds = 10,
                         params = params)
  
  outcome_mod$mort90_xgb_cv <- xgb.cv(data = xgb.DMatrix(data  = m_sm,
                                             label = m_d_risk$ts_mort_event_itt),
                         nrounds = 500,
                         nfold = 5,
                         prediction = T, 
                         early_stopping_rounds = 10,
                         params = params)
  
  outcome_mod$readmit7_xgb_cv <- xgb.cv(data = xgb.DMatrix(data  = m_sm,
                                            label = m_d_risk$ts_readmit_event_itt_7 == "Readmission"),
                         nrounds = 500,
                         nfold = 5,
                         prediction = T, 
                         early_stopping_rounds = 10,
                         params = params)
  outcome_mod$readmit30_xgb_cv <- xgb.cv(data = xgb.DMatrix(data  = m_sm,
                                             label = m_d_risk$ts_readmit_event_itt_30 == "Readmission"),
                         nrounds = 500,
                         nfold = 5,
                         prediction = T, 
                         early_stopping_rounds = 10,
                         params = params)
  outcome_mod$readmit90_xgb_cv <- xgb.cv(data = xgb.DMatrix(data  = m_sm,
                                             label = m_d_risk$ts_readmit_event_itt == "Readmission"),
                         nrounds = 500,
                         nfold = 5,
                         prediction = T, 
                         early_stopping_rounds = 10,
                         params = params)
  
    outcome_mod$any7_xgb_cv <- xgb.cv(data = xgb.DMatrix(data  = m_sm,
                                            label = m_d_risk$ts_any_event_itt_7),
                         nrounds = 500,
                         nfold = 5,
                         prediction = T, 
                         early_stopping_rounds = 10,
                         params = params)
  
  outcome_mod$any30_xgb_cv <- xgb.cv(data = xgb.DMatrix(data  = m_sm,
                                             label = m_d_risk$ts_any_event_itt_30),
                         nrounds = 500,
                         nfold = 5,
                         prediction = T, 
                         early_stopping_rounds = 10,
                         params = params)
  
  outcome_mod$any90_xgb_cv <- xgb.cv(data = xgb.DMatrix(data  = m_sm,
                                             label = m_d_risk$ts_any_event_itt),
                         nrounds = 500,
                         nfold = 5,
                         prediction = T, 
                         early_stopping_rounds = 10,
                         params = params)
  
  
  save(outcome_mod,file = "./outcome_mod.rda")
}

  preds <- data.frame(mort7_pred = unlist(outcome_mod$mort7_xgb_cv$cv_predict),
                      mort30_pred = unlist(outcome_mod$mort30_xgb_cv$cv_predict),
                      mort90_pred = unlist(outcome_mod$mort90_xgb_cv$cv_predict),
                      readmit7_pred = unlist(outcome_mod$readmit7_xgb_cv$cv_predict),
                      readmit30_pred = unlist(outcome_mod$readmit30_xgb_cv$cv_predict),
                      readmit90_pred = unlist(outcome_mod$readmit90_xgb_cv$cv_predict),
                      any7_pred = unlist(outcome_mod$any7_xgb_cv$cv_predict),
                      any30_pred = unlist(outcome_mod$any30_xgb_cv$cv_predict),
                      any90_pred = unlist(outcome_mod$any90_xgb_cv$cv_predict))
  
  
  m_data <- m_data %>%
    as_tibble() %>%
    ungroup() %>%
    bind_cols(preds)
```

# Matching table

```
meanfun <- function(data, i){
  d <- data[i]
  return(mean(d,na.rm=T))   
}

boot_cis <- function(d, s = meanfun, r=100,rd = 2,paste = T){
  bo <- boot(d, statistic=s, R=r)
  bci <- boot.ci(bo, conf=0.95,type = "perc")
  
  if(paste){
    o <- paste0(round(bci$t0,rd)," (",
              round(bci$percent[4],rd),"-",
              round(bci$percent[5],rd),")")
  } else{
    o <- list(bci$t0,
              bci$percent[4],
              bci$percent[5])
  }
  
  return(o)
}

m_strat <- m_data %>%
  group_by(disch_sabo) %>%
  summarise(n = as.character(n()),
            mean_age = boot_cis(age),
            pct_female = boot_cis(gender),
            mean_caredays = boot_cis(caredays),
            mean_n_op = boot_cis(n_op),
            mean_n_diag = boot_cis(n_diag),
            mean_days_since_prev = boot_cis(days_since_prev),
            mean_count_yr = boot_cis(count_yr),
            mean_count_yr_unplan = boot_cis(count_yr_unplanned),
            mean_hc_prev = boot_cis(n_hc), 
            mean_htj_prev = boot_cis(n_htj), 
            mean_amb_unplanned = boot_cis(count_yr_ov_unplanned),
            mean_amb_planned = boot_cis(count_yr_ov_planned),
            pct_unplanreadmit30 = boot_cis(unplanreadmit30),
            pct_mort30 = boot_cis(mort30)) %>%
  pivot_longer(cols = -c(disch_sabo)) %>%
  mutate(discharge = ifelse(disch_sabo == 1,"NH","Home"))

d_strat <- d %>%
  group_by(disch_sabo) %>%
  summarise(n = as.character(n()),
            mean_age = boot_cis(age),
            pct_female = boot_cis(gender),
            mean_caredays = boot_cis(caredays),
            mean_n_op = boot_cis(n_op),
            mean_n_diag = boot_cis(n_diag),
            mean_days_since_prev = boot_cis(days_since_prev),
            mean_count_yr = boot_cis(count_yr),
            mean_count_yr_unplan = boot_cis(count_yr_unplanned),
            mean_hc_prev = boot_cis(n_hc),
            mean_htj_prev = boot_cis(n_htj),
            mean_amb_unplanned = boot_cis(count_yr_ov_unplanned),
            mean_amb_planned = boot_cis(count_yr_ov_planned),
            pct_unplanreadmit30 = boot_cis(unplanreadmit30),
            pct_mort30 = boot_cis(mort30)) %>%
  pivot_longer(cols = -c(disch_sabo)) %>%
  mutate(discharge = ifelse(disch_sabo == 1,"NH","Home"))

smd <- m_data %>%
  as_tibble() %>%
  select(disch_sabo,
         mean_age = age,
         mean_caredays = caredays,
         mean_n_op = n_op,
         mean_n_diag = n_diag,
         mean_days_since_prev = days_since_prev,
         mean_count_yr = count_yr,
         mean_count_yr_unplan = count_yr_unplanned,
         mean_hc_prev = n_hc,
         mean_htj_prev = n_htj,
         mean_amb_unplanned = count_yr_ov_unplanned,
         mean_amb_planned = count_yr_ov_planned) %>%
  pivot_longer(-disch_sabo, names_to = "variable", values_to = "value") %>%
  group_by(variable) %>%
  summarise(effectsize::cohens_d(value ~ disch_sabo, ci = 0.95)) %>%
  mutate(pasted = paste0(round(Cohens_d,3)," (",round(CI_low,3),"-",round(CI_high,3),")"))

#Balance in original data
d_strat_wide <- d_strat %>%
  select(-disch_sabo) %>%
  pivot_wider(names_from = discharge,
              values_from = value,
              names_prefix = "raw ")

#Balance in matched data
m_strat_wide <- m_strat %>%
  select(-disch_sabo) %>%
  pivot_wider(names_from = discharge,
              values_from = value,
              names_prefix = "matched ")

match_table <- bind_cols(d_strat_wide,select(m_strat_wide,-name)) %>%
  left_join(select(smd,name = variable,smd_ci = pasted))

kable(match_table)
```

| name | raw Home | raw NH | matched Home | matched NH | smd\_ci |
| --- | --- | --- | --- | --- | --- |
| n | 343183 | 69890 | 47615 | 47615 | NA |
| mean\_age | 82.02 (82-82.05) | 83.97 (83.91-84.03) | 83.29 (83.22-83.38) | 83.68 (83.6-83.75) | -0.05 (-0.063–0.037) |
| pct\_female | 0.59 (0.59-0.59) | 0.59 (0.59-0.59) | 0.59 (0.58-0.59) | 0.59 (0.58-0.59) | NA |
| mean\_caredays | 10.07 (10.04-10.09) | 15.97 (15.85-16.06) | 15.17 (15.06-15.26) | 13.99 (13.91-14.06) | 0.12 (0.107-0.132) |
| mean\_n\_op | 2.56 (2.55-2.57) | 3.09 (3.06-3.11) | 3.14 (3.11-3.17) | 2.85 (2.82-2.87) | 0.095 (0.082-0.108) |
| mean\_n\_diag | 5.74 (5.73-5.75) | 6.03 (6-6.05) | 5.93 (5.89-5.96) | 5.81 (5.79-5.84) | 0.038 (0.026-0.051) |
| mean\_days\_since\_prev | 90.85 (90.44-91.31) | 83.86 (82.9-85.26) | 87.58 (86.48-88.94) | 82.49 (81.19-83.97) | 0.052 (0.034-0.071) |
| mean\_count\_yr | 1.65 (1.64-1.66) | 1.05 (1.04-1.06) | 0.97 (0.96-0.98) | 1.1 (1.09-1.12) | -0.08 (-0.093–0.067) |
| mean\_count\_yr\_unplan | 1.48 (1.47-1.48) | 0.95 (0.94-0.96) | 0.86 (0.84-0.87) | 1 (0.98-1.01) | -0.092 (-0.105–0.08) |
| mean\_hc\_prev | 3.96 (3.93-3.99) | 0.28 (0.26-0.3) | 1.49 (1.44-1.52) | 0.3 (0.28-0.32) | 0.34 (0.328-0.353) |
| mean\_htj\_prev | 8.58 (8.54-8.62) | 0.68 (0.65-0.71) | 2.85 (2.79-2.92) | 0.83 (0.79-0.88) | 0.362 (0.35-0.375) |
| mean\_amb\_unplanned | 2.53 (2.52-2.54) | 1.63 (1.61-1.65) | 1.51 (1.48-1.53) | 1.69 (1.67-1.71) | -0.071 (-0.084–0.059) |
| mean\_amb\_planned | 2.9 (2.87-2.92) | 1.53 (1.48-1.59) | 1.7 (1.65-1.76) | 1.62 (1.56-1.7) | 0.012 (-0.001-0.025) |
| pct\_unplanreadmit30 | 0.25 (0.24-0.25) | 0.15 (0.15-0.15) | 0.18 (0.18-0.19) | 0.15 (0.14-0.15) | NA |
| pct\_mort30 | 0.05 (0.04-0.05) | 0.08 (0.08-0.08) | 0.06 (0.06-0.07) | 0.08 (0.08-0.08) | NA |

# Categorical predictor distributions

## Major Diagnostic Group

```
d_eval %>%
  left_join(mdc_df) %>%
  group_by(disch_sabo,mdc_name) %>%
  summarise(n = n()) %>%
  group_by(discharge = ifelse(disch_sabo == 1,"NH","Home")) %>%
  mutate(pct = n/sum(n)) %>%
  ggplot(aes(y=pct,x= mdc_name,fill = discharge)) +
  geom_bar(stat="identity", position = position_dodge()) +
  labs(y= "% of patients with MDC  (raw)") +
  theme(axis.text.x = element_text(angle = 90,hjust = 1,vjust = 0.3))
```

```
m_data %>%
  left_join(mdc_df) %>%
  group_by(disch_sabo,mdc_name) %>%
  summarise(n = n()) %>%
  group_by(discharge = ifelse(disch_sabo == 1,"NH","Home")) %>%
  mutate(pct = n/sum(n)) %>%
  ggplot(aes(y=pct,x= mdc_name,fill = discharge)) +
  geom_bar(stat="identity", position = position_dodge()) +
  labs(y= "% of patients with MDC  (raw)") +
  theme(axis.text.x = element_text(angle = 90,hjust = 1,vjust = 0.3))
```

## Hospital Ward

```
d_eval %>%
  group_by(disch_sabo,mvo_last) %>%
  summarise(n = n()) %>%
  group_by(discharge = ifelse(disch_sabo == 1,"NH","Home")) %>%
  mutate(pct = n/sum(n)) %>%
  ggplot(aes(y=pct,x= as.factor(mvo_last),fill = discharge)) +
  geom_bar(stat="identity", position = position_dodge()) +
  labs(y= "Percent of patients with ward code (raw)") +
  theme(axis.text.x = element_text(angle = 90,hjust = 1,vjust = 0.3))
```

```
  labs(x = "Hospital ward code")
```

```
## <ggplot2::labels> List of 1
##  $ x: chr "Hospital ward code"
```

```
m_data %>%
  group_by(disch_sabo,mvo_last) %>%
  summarise(n = n()) %>%
  group_by(discharge = ifelse(disch_sabo == 1,"NH","Home")) %>%
  mutate(pct = n/sum(n)) %>%
  ggplot(aes(y=pct,x= as.factor(mvo_last),fill = discharge)) +
  geom_bar(stat="identity", position = position_dodge()) +
  labs(y= "Percent of patients with ward code (raw)") +
  theme(axis.text.x = element_text(angle = 90,hjust = 1,vjust = 0.3))
```

```
  labs(x = "Hospital ward code")
```

```
## <ggplot2::labels> List of 1
##  $ x: chr "Hospital ward code"
```

These codes are a bit difficult to translate and likely have limited
interpretability outside of Sweden anyway… Ward code lists may be found
with the National Board of Health and Welfare: https://www.socialstyrelsen.se/globalassets/sharepoint-dokument/dokument-webb/klassifikationer-och-koder/sjukhuskoder-kodlista-verksamhetsomraden-2006.pdf

## Region

```
d_eval %>%
  group_by(disch_sabo,region) %>%
  summarise(n = n()) %>%
  group_by(discharge = ifelse(disch_sabo == 1,"NH","Home")) %>%
  mutate(pct = n/sum(n)) %>%
  ggplot(aes(y=pct,x= as.factor(region),fill = discharge)) +
  geom_bar(stat="identity", position = position_dodge()) +
  labs(y= "Percent of patients in region (raw)") +
  theme(axis.text.x = element_text(angle = 90,hjust = 1,vjust = 0.3)) +
  labs(x = "Region")
```

```
m_data %>%
  group_by(disch_sabo,region) %>%
  summarise(n = n()) %>%
  group_by(discharge = ifelse(disch_sabo == 1,"NH","Home")) %>%
  mutate(pct = n/sum(n)) %>%
  ggplot(aes(y=pct,x= as.factor(region),fill = discharge)) +
  geom_bar(stat="identity", position = position_dodge()) +
  labs(y= "Percent of patients in region (raw)") +
  theme(axis.text.x = element_text(angle = 90,hjust = 1,vjust = 0.3)) +
  labs(x = "Region")
```

# Propensity score distribution in raw and matched samples

```
ggplot(d_eval, aes(x = nh_propensity, 
              fill = as.factor(disch_sabo))) +
  geom_density(alpha = 0.5, colour = "grey50") +
  #geom_point(aes(y= 0,x=nh_propensity,)) +
  geom_rug(aes(color = prop_strat)) +
  scale_color_brewer(palette = "Spectral")
```

```
d_eval %>%
  group_by(prop_strat,disch_sabo) %>%
  summarise(n = n()) %>%
  pivot_wider(names_from = disch_sabo,
              values_from = n)
```

```
ggplot(m_data, aes(x = nh_propensity, 
              fill = as.factor(disch_sabo))) +
  geom_density(alpha = 0.5, colour = "grey50") +
  #geom_point(aes(y= 0,x=nh_propensity,)) +
  geom_rug(aes(color = prop_strat)) +
  scale_color_brewer(palette = "Spectral")
```

```
m_data %>%
  group_by(prop_strat,disch_sabo) %>%
  summarise(n = n()) %>%
  pivot_wider(names_from = disch_sabo,
              values_from = n)
```

# Propensity score variable description

```
description <- c("caredays" = "Duration of hospital stay",  
                 "diagsec" = "Secondary diagnosis", 
                 "age" = "Patient age", 
                 "region" = "Region", 
                 "muni" = "Municipality", 
                 "mvo" = "Hospital ward", 
                 "date" = "Date of discharge", 
                 "prevSince" = "Days since previous hospital stay", 
                 "countYrUnplanned" = "Number of unplanned hospital stays in the last year", 
                 "countHomecareMonths" = "Number of months of home health care prior to discharge",
                 "countHomeserviceMonths" = "Number of months of home services prior to discharge",
                 "countAmbPlanned" = "Number of planned contacts with ambulatory care in the previous year",
                 "countAmbUnplanned" = "Number of unplanned contacts with ambulatory care in the previous year",
                 "countDiags" = "Number of diagnosis codes recorded during hospital stay",
                 "countInterventions" = "Number of intervention codes recorded during hospital stay",
                 "prevCaredays" = "Duration of previous hostial stay", 
                 "week" = "Week of discharge", 
                 "hosp" = "Discharging hospital", 
                 "civil" = "Patient civil status", 
                 "diagprim" = "Patient primary diagnosis", 
                 "op" = "Operation (KVÅ) code", 
                 "countYr" = "Number of hospital stays in the last year", 
                 "female" = "Sex of patient", 
                 "weekday" = "Weekday of discharge", 
                 "born" = "Patient region of birth", 
                 "prevdiag" = "Primary diagnosis during previous hospital stay")

importance_matrix <- xgb.importance(propensity_xgb) %>%
  mutate(type = str_split_i(Feature,"_",1)) %>%
  left_join(as_tibble(list(type = names(description),
                      description = description)))

t <- xgb.plot.shap.summary(data = sm, model = propensity_xgb,top_n = 100)

mean_shap <- t$data %>%
  mutate(valshap = feature_value*shap_value) %>%
  group_by(Feature = feature) %>%
  summarise(mean_shap = mean(valshap))

importance_matrix <- importance_matrix %>%
  left_join(mean_shap) %>%
  select(description,Feature,Gain,Cover,mean_shap)

vars <- colnames(m_sm)[colnames(m_sm) %in% importance_matrix$Feature]

smd_matched <- m_sm[,vars] %>%
  as.matrix() %>%
  as_tibble() %>%
  bind_cols(select(m_data,disch_sabo)) %>%
  pivot_longer(-disch_sabo, names_to = "variable", values_to = "value") %>%
  group_by(variable) %>%
  summarise(effectsize::cohens_d(value ~ disch_sabo)) %>%
  left_join(importance_matrix,by = c("variable"="Feature")) %>%
  mutate(smd_95ci = paste0(round(Cohens_d,3)," (",round(CI_low,3),":",round(CI_high,3),")")) %>%
  transmute(variable,
            description,
            smd_95ci,
            Gain = round(Gain,5),
            Cover = round(Cover,5),
            mean_shap = round(mean_shap,5),
            Cohens_d) %>%
  arrange(desc(Gain))
```

## Variable summary

```
importance_matrix %>%
  group_by(description) %>%
  summarise(n_values = n(),
            sum_gain = sum(Gain)) %>%
  arrange(desc(sum_gain)) %>%
  kable()
```

| description | n\_values | sum\_gain |
| --- | --- | --- |
| Number of months of home services prior to discharge | 1 | 0.3641464 |
| Secondary diagnosis | 603 | 0.1256795 |
| Duration of hospital stay | 1 | 0.1109751 |
| Number of months of home health care prior to discharge | 1 | 0.0596538 |
| Municipality | 203 | 0.0474117 |
| Number of unplanned contacts with ambulatory care in the previous year | 1 | 0.0449320 |
| Patient age | 1 | 0.0387139 |
| Region | 20 | 0.0373902 |
| Operation (KVÅ) code | 286 | 0.0298589 |
| Hospital ward | 36 | 0.0293212 |
| Discharging hospital | 73 | 0.0156222 |
| Date of discharge | 1 | 0.0148339 |
| Days since previous hospital stay | 1 | 0.0111590 |
| Number of unplanned hospital stays in the last year | 1 | 0.0096172 |
| Patient primary diagnosis | 187 | 0.0088950 |
| Duration of previous hostial stay | 1 | 0.0082234 |
| Week of discharge | 1 | 0.0073195 |
| Number of intervention codes recorded during hospital stay | 1 | 0.0068706 |
| Number of planned contacts with ambulatory care in the previous year | 1 | 0.0058187 |
| Patient civil status | 4 | 0.0055297 |
| Number of diagnosis codes recorded during hospital stay | 1 | 0.0048772 |
| Primary diagnosis during previous hospital stay | 128 | 0.0041403 |
| Weekday of discharge | 7 | 0.0039231 |
| Sex of patient | 1 | 0.0019593 |
| Number of hospital stays in the last year | 1 | 0.0018019 |
| Patient region of birth | 4 | 0.0013262 |

## Detailed variable table

Note: Higher SHAP values indicate a marginal, linear association with
a higher likelihood of discharge to NH. Note that many ICD codes were
masked by the providers of the registry data to maintain patient
anonymity.

```
datatable(select(smd_matched,-Cohens_d))
```

## Partial dependence plot

```
xgb.plot.shap(sm, model = propensity_xgb,top_n = 5)
```

# SMD for all predictors

```
smd_matched %>%
  ggplot(aes(x=Cohens_d,y=reorder(variable,Gain))) +
  geom_point() +
  theme(
    axis.text.y = element_blank(),
    axis.ticks.y = element_blank() )+
  
  labs(y = "Parameter in propensity score model",
       x = "Standardized Mean Difference")
```

# Main analysis

## Estimate models

```
if(file.exists("./cif.rda") & !reload){
  load("./cif.rda")
  load("./hr.rda")
}else{
  
  cif <- list()
  hr <- list()
  
  cif$mort <- tidycmprsk::cuminc(Surv(ts_mort_days_itt, 
                                      as.factor(ts_mort_event_itt)) ~ disch_sabo, 
                     cluster = lopnr, 
                     d_eval)
  
  cif$mort_prop <- tidycmprsk::cuminc(Surv(ts_mort_days_itt, 
                                           as.factor(ts_mort_event_itt)) ~ disch_sabo,
                     cluster = subclass, 
                     m_data)
  
  hr$mort7 <- coxph(Surv(ts_mort_days_itt_7,
                         ts_mort_event_itt_7) ~ disch_sabo,
                    cluster = lopnr,
                    data = d_eval)
  
  hr$mort30 <- coxph(Surv(ts_mort_days_itt_30,
                         ts_mort_event_itt_30) ~ disch_sabo,
                    cluster = lopnr,
                    data = d_eval)
  
  hr$mort90 <- coxph(Surv(ts_mort_days_itt,
                         ts_mort_event_itt) ~ disch_sabo,
                    cluster = lopnr,
                    data = d_eval)
  
  hr$mort7_prop <- coxph(Surv(ts_mort_days_itt_7,
                         ts_mort_event_itt_7) ~ disch_sabo,
                    cluster = subclass,
                    data = m_data)
  
  hr$mort30_prop <- coxph(Surv(ts_mort_days_itt_30,
                         ts_mort_event_itt_30) ~ disch_sabo,
                    cluster = subclass,
                    data = m_data)
  hr$mort90_prop <- coxph(Surv(ts_mort_days_itt,
                         ts_mort_event_itt) ~ disch_sabo,
                    cluster = subclass,
                    data = m_data)
  
  hr$mort7_dr <- coxph(Surv(ts_mort_days_itt_7,
                         ts_mort_event_itt_7) ~ disch_sabo + 
                         mort7_pred,
                    cluster = subclass,
                    data = m_data)
  
  hr$mort30_dr <- coxph(Surv(ts_mort_days_itt_30,
                         ts_mort_event_itt_30) ~ disch_sabo + mort30_pred,
                    cluster = subclass,
                    data = m_data)
  hr$mort90_dr <- coxph(Surv(ts_mort_days_itt,
                         ts_mort_event_itt) ~ disch_sabo + mort90_pred,
                    cluster = subclass,
                    data = m_data)
  
  ## Readmission
  
  
  cif$readmit <- tidycmprsk::cuminc(Surv(ts_readmit_days_itt, 
                                                     ts_readmit_event_itt) ~ disch_sabo,
                     cluster = lopnr, 
                     d_eval)
  
  cif$readmit_prop <- tidycmprsk::cuminc(Surv(ts_readmit_days_itt, 
                                              ts_readmit_event_itt) ~ disch_sabo,
                     cluster = subclass, 
                     m_data)
  
  # cif$readmit_prop_dr <- tidycmprsk::cuminc(Surv(ts_readmit_days_itt, 
  #                                                         as.numeric(ts_readmit_event_itt)) ~ disch_sabo + readmit30_pred,
  #                    cluster = subclass, 
  #                    m_data)
  
  library(fastcmprsk)
  
  hr$readmit7 <- fastCrr(Crisk(ts_readmit_days_itt_7,ts_readmit_event_itt_7,
                         failcode = "Readmission",
                         cencode = "Censored") ~ disch_sabo,
                    data = d_eval)
  hr$readmit30 <- fastCrr(Crisk(ts_readmit_days_itt_30,ts_readmit_event_itt_30,
                         failcode = "Readmission",
                         cencode = "Censored") ~ disch_sabo,
                    data = d_eval)
  hr$readmit90 <- fastCrr(Crisk(ts_readmit_days_itt,ts_readmit_event_itt,
                         failcode = "Readmission",
                         cencode = "Censored") ~ disch_sabo,
                    data = d_eval)
  
  hr$readmit7_prop <- fastCrr(Crisk(ts_readmit_days_itt_7,ts_readmit_event_itt_7,
                         failcode = "Readmission",
                         cencode = "Censored") ~ disch_sabo,
                    data = m_data)
  hr$readmit30_prop <- fastCrr(Crisk(ts_readmit_days_itt_30,ts_readmit_event_itt_30,
                         failcode = "Readmission",
                         cencode = "Censored") ~ disch_sabo,
                    data = m_data)
  hr$readmit90_prop <- fastCrr(Crisk(ts_readmit_days_itt,ts_readmit_event_itt,
                         failcode = "Readmission",
                         cencode = "Censored") ~ disch_sabo,
                    data = m_data)
  
  hr$readmit7_dr <- fastCrr(Crisk(ts_readmit_days_itt_7,ts_readmit_event_itt_7,
                         failcode = "Readmission",
                         cencode = "Censored") ~ disch_sabo + readmit7_pred,
                    data = m_data)
  hr$readmit30_dr <- fastCrr(Crisk(ts_readmit_days_itt_30,ts_readmit_event_itt_30,
                         failcode = "Readmission",
                         cencode = "Censored") ~ disch_sabo + readmit30_pred,
                    data = m_data)
  hr$readmit90_dr <- fastCrr(Crisk(ts_readmit_days_itt,ts_readmit_event_itt,
                         failcode = "Readmission",
                         cencode = "Censored") ~ disch_sabo + readmit90_pred,
                    data = m_data)
  
  
  # Composite outcome
  
    cif$any <- tidycmprsk::cuminc(Surv(ts_any_days_itt, 
                                      as.factor(ts_any_event_itt)) ~ disch_sabo, 
                     cluster = lopnr, 
                     d_eval)
  
  cif$any_prop <- tidycmprsk::cuminc(Surv(ts_any_days_itt, 
                                           as.factor(ts_any_event_itt)) ~ disch_sabo,
                     cluster = subclass, 
                     m_data)
  
  hr$any7 <- coxph(Surv(ts_any_days_itt_7,
                         ts_any_event_itt_7) ~ disch_sabo,
                    cluster = lopnr,
                    data = d_eval)
  
  hr$any30 <- coxph(Surv(ts_any_days_itt_30,
                         ts_any_event_itt_30) ~ disch_sabo,
                    cluster = lopnr,
                    data = d_eval)
  
  hr$any90 <- coxph(Surv(ts_any_days_itt,
                         ts_any_event_itt) ~ disch_sabo,
                    cluster = lopnr,
                    data = d_eval)
  
  hr$any7_prop <- coxph(Surv(ts_any_days_itt_7,
                         ts_any_event_itt_7) ~ disch_sabo,
                    cluster = subclass,
                    data = m_data)
  
  hr$any30_prop <- coxph(Surv(ts_any_days_itt_30,
                         ts_any_event_itt_30) ~ disch_sabo,
                    cluster = subclass,
                    data = m_data)
  hr$any90_prop <- coxph(Surv(ts_any_days_itt,
                         ts_any_event_itt) ~ disch_sabo,
                    cluster = subclass,
                    data = m_data)
  
  hr$any7_dr <- coxph(Surv(ts_any_days_itt_7,
                         ts_any_event_itt_7) ~ disch_sabo + 
                         any7_pred,
                    cluster = subclass,
                    data = m_data)
  
  hr$any30_dr <- coxph(Surv(ts_any_days_itt_30,
                         ts_any_event_itt_30) ~ disch_sabo + any30_pred,
                    cluster = subclass,
                    data = m_data)
  hr$any90_dr <- coxph(Surv(ts_any_days_itt,
                         ts_any_event_itt) ~ disch_sabo + any90_pred,
                    cluster = subclass,
                    data = m_data)
  
  save(cif,file = "./cif.rda")
  save(hr,file = "./hr.rda")
}
```

## Plot cumulative incidence curves

```
cif$mort %>%
  ggcuminc(outcome = "TRUE") +
  add_confidence_interval() +
  labs(y = "Mortality cumulative incidence (raw)",
       x = "Days since discharge") + 
  scale_color_manual(values = c("red", "blue")) +
  scale_fill_manual(values = c("red", "blue")) +
  geom_vline(xintercept = c(7,30,90))
```

```
cif$mort_prop %>%
  ggcuminc(outcome = "TRUE") +
  add_confidence_interval() +
  labs(y = "Mortality cumulative incidence (matched)",
       x = "Days since discharge") + 
  scale_color_manual(values = c("red", "blue")) +
  scale_fill_manual(values = c("red", "blue")) +
  geom_vline(xintercept = c(7,30,90))
```

```
cif$readmit %>%
  ggcuminc(outcome = "Readmission") +
  add_confidence_interval() +
  labs(y = "Readmission cumulative incidence (raw)",
       x = "Days since discharge") + 
  scale_color_manual(values = c("red", "blue")) +
  scale_fill_manual(values = c("red", "blue")) +
  geom_vline(xintercept = c(7,30,90))
```

```
cif$readmit_prop %>%
  ggcuminc(outcome = "Readmission") +
  add_confidence_interval() +
  labs(y = "Readmission cumulative incidence (matched)",
       x = "Days since discharge") + 
  scale_color_manual(values = c("red", "blue")) +
  scale_fill_manual(values = c("red", "blue")) +
  geom_vline(xintercept = c(7,30,90))
```

```
cif$any %>%
  ggcuminc(outcome = "TRUE") +
  add_confidence_interval() +
  labs(y = "Composite cumulative incidence (raw)",
       x = "Days since discharge") + 
  scale_color_manual(values = c("red", "blue")) +
  scale_fill_manual(values = c("red", "blue")) +
  geom_vline(xintercept = c(7,30,90))
```

```
cif$any_prop %>%
  ggcuminc(outcome = "TRUE") +
  add_confidence_interval() +
  labs(y = "Composite cumulative incidence (matched)",
       x = "Days since discharge") + 
  scale_color_manual(values = c("red", "blue")) +
  scale_fill_manual(values = c("red", "blue")) +
  geom_vline(xintercept = c(7,30,90))
```

## Hazard ratio table

```
paste_cox_ci <- function(mod,r=3){
  paste0(round(exp(coef(mod)["disch_sabo"]),r), " (",
        round(exp(confint(mod)["disch_sabo",1]),r),"-",
        round(exp(confint(mod)["disch_sabo",2]),r),")")
}

paste_crr_ci <- function(mod,r=3){
  
  ci <- confint(mod)
  if(first(class(ci)) == "matrix"){
    ci <- ci[1,]
  }

  paste0(round(exp(coef(mod)[1]),r), " (",
        round(exp(ci[1]),r),"-",
        round(exp(ci[2]),r),")")
}

if(file.exists("./outcome_table.rda") & !reload){
  load("./outcome_table.rda")
}else{

raw <- data.frame("raw" = c(paste_cox_ci(hr$mort7),
                            paste_cox_ci(hr$mort30),
                            paste_cox_ci(hr$mort90),
                            paste_crr_ci(hr$readmit7),
                            paste_crr_ci(hr$readmit30),
                            paste_crr_ci(hr$readmit90),
                            paste_crr_ci(hr$any7),
                            paste_crr_ci(hr$any30),
                            paste_crr_ci(hr$any90)))

matched <- data.frame("matched" = c(paste_cox_ci(hr$mort7_prop),
                            paste_cox_ci(hr$mort30_prop),
                            paste_cox_ci(hr$mort90_prop),
                            paste_crr_ci(hr$readmit7_prop),
                            paste_crr_ci(hr$readmit30_prop),
                            paste_crr_ci(hr$readmit90_prop),
                            paste_crr_ci(hr$any7_prop),
                            paste_crr_ci(hr$any30_prop),
                            paste_crr_ci(hr$any90_prop)))

doublerobust <- data.frame("doublerobust" = c(paste_cox_ci(hr$mort7_dr),
                            paste_cox_ci(hr$mort30_dr),
                            paste_cox_ci(hr$mort90_dr),
                            paste_crr_ci(hr$readmit7_dr),
                            paste_crr_ci(hr$readmit30_dr),
                            paste_crr_ci(hr$readmit90_dr),
                            paste_crr_ci(hr$any7_dr),
                            paste_crr_ci(hr$any30_dr),
                            paste_crr_ci(hr$any90_dr)))

outcome_table <- data.frame("outcome" = c(rep("Mortality",3),
                                          rep("Readmission",3),
                                          rep("Composite",3)),
                            "Time" = c(rep(c("7","30","90"),3))) %>%
  bind_cols(raw) %>%
  bind_cols(matched) %>%
  bind_cols(doublerobust)

  save(outcome_table,file = "./outcome_table.rda")

}

kable(outcome_table)
```

| outcome | Time | raw | matched | doublerobust |
| --- | --- | --- | --- | --- |
| Mortality | 7 | 1.423 (1.321-1.533) | 0.869 (0.779-0.969) | 0.927 (0.827-1.041) |
| Mortality | 30 | 1.806 (1.75-1.864) | 1.314 (1.253-1.377) | 1.264 (1.204-1.328) |
| Mortality | 90 | 1.794 (1.756-1.832) | 1.591 (1.542-1.641) | 1.43 (1.384-1.477) |
| Readmission | 7 | 0.509 (0.493-0.526) | 0.62 (0.587-0.655) | 0.655 (0.619-0.693) |
| Readmission | 30 | 0.585 (0.574-0.596) | 0.788 (0.763-0.813) | 0.819 (0.793-0.846) |
| Readmission | 90 | 0.628 (0.618-0.638) | 0.908 (0.888-0.928) | 0.909 (0.889-0.929) |
| Composite | 7 | 0.587 (0.569-0.607) | 0.668 (0.636-0.701) | 0.716 (0.681-0.752) |
| Composite | 30 | 0.758 (0.744-0.771) | 0.944 (0.918-0.97) | 0.948 (0.923-0.975) |
| Composite | 90 | 0.853 (0.841-0.864) | 1.143 (1.12-1.166) | 1.06 (1.038-1.082) |
