# Supplementary material for: Estimating the impact of discharge to nursing home on readmission and mortality: a propensity score matched analysis
Source: BMC Geriatr. 2026 Jul 4;26:909. doi: 10.1186/s12877-026-07761-8 (PMC13339853; doi:10.1186/s12877-026-07761-8)
Supplement: Supplementary file 4 — Supplementary Material 4. [file 12877_2026_7761_MOESM4_ESM.html]

The causal impact of discharge to nursing home on readmission and mortality: A propensity score matched survival analysis


# The causal impact of discharge to nursing home on readmission and mortality: A propensity score matched survival analysis

- Setup
- Load data
- Train propensity models
- Perform
  matching
- Propensity score validation
  - mean values by percentile
- Pseudo
  r-squared
- Generate outcome
  predictions
- Matching
  table
- Categorical predictor
  distributions
  - Major Diagnostic Group
  - Hospital
    Ward
  - Region
- Propensity
  score distribution in raw and matched samples
- Propensity score variable
  description
  - Variable
    summary
  - Detailed variable table
  - Partial dependence plot
- SMD for all predictors
- Main analysis
  - Estimate
    models
  - Plot cumulative incidence
    curves
  - Hazard
    ratio table

# Setup

```
knitr::opts_chunk$set(cache = FALSE, 
                      warning = FALSE, 
                      message = FALSE, 
                      cache.lazy = FALSE)

options(scipen = 999,cache.lazy = FALSE)

set.seed(42)

# Set flag to regenerate stored artifacts
reload = F

# set to "glm_propensity" for sensitivity analysis concerning effect of alternate propensity score model
# set to "noqualityexcl" for sensitivity analysis concerning effect without excluding discharges at risk for documentation errors
# set to "first_patient" for sensitivity analysis concerning effect of including only the first contact for each patient (instead of excluding repeat patients at the matching stage)
sens_analysis <- "first_patient"

path = paste0(getwd(),"/",sens_analysis)
dir.create(path)
knitr::opts_knit$set(root.dir = path)

suppressPackageStartupMessages({

  library(tidycmprsk)
  library(ggsurvfit)
  library(readxl)
  library(lubridate)
  library(boot)
  library(xgboost)
  library(data.table)
  library(knitr)
  library(tidyverse)
  library(MatchIt)
  library(marginaleffects)
  library(survival)
  library(survminer)
  library(Matrix)
  library(cmprsk)
  library(glmnet)
  library(effectsize)
  library(DT)
})
```

```
# Define MDC codes based on NBHW definitions https://www.socialstyrelsen.se/statistik-och-data/klassifikationer-och-koder/drg/drg-koder-och-definitioner/


mdc = c("A" = "Nervous System", 
        "B" = "Eye", 
        "C" = "Ear, Nose, Mouth, And Throat", 
        "D" = "Respiratory System", 
        "E" = "Circulatory System", 
        "F" = "Digestive System", 
        "G" = "Hepatobiliary System and Pancreas", 
        "H" = "Musculoskeletal System \n and Connective Tissue", 
        "J" = "Skin, Subcutaneous \nTissue, and Breast", 
        "K" = "Mammary gland diseases", 
        "L" = "Endocrine, Nutritional,\n and Metabolic System", 
        "M" = "Kidney and Urinary Tract", 
        "N" = "Male Reproductive System", 
        "O" = "Female Reproductive System", 
        "P" = "Pregnancy, Childbirth, and Puerperium", 
        "Q" = "Newborn and Other Neonates (Perinatal Period)",
        "R" = "Blood/-Forming and Immun./\nMyeloprolif. Dis. / NS tumors", 
        "S" = "Infectious and Parasitic\n Diseases and Disorders", 
        "T" = "Mental Diseases and Disorders", 
        "U" = "Injuries, Poison, \nand Toxic Effect of Drugs", 
        "V" = "Burns", 
        "W" = "Factors Influencing Health Status", 
        "Z" = "Ungroupable")

mdc_df <- data.frame(mdc = names(mdc),
                     mdc_name = mdc)

## Set parameters

  # Maximum length of stay to include
  max_caredays = 90
  
  # Minimum number of observations from a hospital ward to include
  min_hosp_mvo_obs = 100 
  
  # Time span to search forwards from a contact to identify readmissions
  fu_days = 90
  
  # Time span to search backwards from a contact to identify previous admissions
  prev_days = 365

# Function for getting bootstrap CIs for mean
mean_fun <- function(data,inds){
  return(mean(data[inds]))
}

# Function for calculating number of contacts within a given timeframe
window_dates <- function(d,s,i){
  d = unlist(d)
  l = length(d[d >= s & d <= i])
  return(l)
}


elapsed_months <- function(end_date, start_date) {

  
  sd <- as.POSIXlt(start_date)
  ed <- as.POSIXlt(end_date)
  
  return(12 * (ed$year - sd$year) + (ed$mon - sd$mon))
}

# Function for calculating the quantile of a value

get_q <- function(x,q){sum(x>unlist(q))/(length(unlist(q)))}

# Function for generating sparse matrix for propensity xgboost model

generate_sparsm <- function(data){
  
  cats <- data %>%
    ungroup() %>%
    dplyr::select(id,
           mvo,
           weekday,
           op = interventions,
           prevdiag = prev_last_diag,
           diagprim = diag_prim_last,
           diagsec = diag_sec,
           region,
           muni,
           hosp,
           born,
           civil,
           admit) %>% 
    mutate(mvo = gsub(" ","|",mvo),
           diagprim = gsub(" ","|",diagprim),
           diagsec = gsub(" ","|",diagsec),
           op = gsub(" ","|",op),
           prevdiag = gsub(" ","|",prevdiag),
           muni = gsub(" ","|",muni)
           ) %>%
    mutate(across(everything(),function(x) strsplit(as.character(x),"\\|"))) %>%
    pivot_longer(-id) %>%
    unnest(cols = value,keep_empty = T) %>%
    mutate(id = as.numeric(id),
           name = make.names(paste(name,value,sep = "_")),
           value = 1) %>%
    distinct() %>%
    ungroup()
  
  ids <- unique(cats$id)
  names <- unique(cats$name)
  
  # Map to sparse row/column indices
  id_map <- data.frame(sm_id = seq(1,length(ids)),
                       id = ids)
  
  name_map <- data.frame(sm_d = seq(1,length(names)),
                         name = names)
  
  # Join to data
  cats <- cats %>%
    left_join(id_map) %>%
    left_join(name_map)
  
  
  sprsM_cat <- as(sparseMatrix(i = cats$sm_id,
                               j = cats$sm_d,
                               x = cats$value,
                               dimnames = list(unique(cats$id)[order(unique(cats$sm_id))],
                                               unique(cats$name)[order(unique(cats$sm_d))])), "dgCMatrix")
  
  
  num <- data %>%
    transmute(date = as.numeric(out_date),
              week,
              caredays = caredays,
              prevSince = ifelse(is.na(prev_caredays),fu_days+1,prev_caredays),
              prevCaredays = ifelse(is.na(days_since_prev),366,days_since_prev),
              planned = planned_contact,
              age,
              countHomecareMonths = n_hc,
              countHomeserviceMonths = n_htj,
              countAmbPlanned = count_yr_ov_planned,
              countAmbUnplanned = count_yr_ov_unplanned,
              countDiags = n_diag,
              countInterventions = n_op,
              female = gender,
              countYr = count_yr,
              countYrUnplanned = count_yr_unplanned) 
  
  
  sprsM <- num %>%
    as.matrix() %>%
    Matrix(sparse = T) %>%
    cbind(sprsM_cat)
  
  return(sprsM)
  
}
```

# Load data

Data = hospital data Sol = SOL data

```
# Load data 

if(file.exists("./data_final.rda") & 
   file.exists("./excl_n.rda") & !reload){
load("data_final.rda")
load("excl_n.rda")
}else{
  
  parent_dir <- dirname(getwd())
  load(paste0(parent_dir,"/data.rda"))
  load(paste0(parent_dir,"/sol.rda"))
  
  d$disch_sabo <- as.numeric(d$discharge == "Särskilt boende")
  
#df indicating which months patient has NH or short term stay 
sabo <- sol %>%
  filter(BOFORM == 2 | KORTTID == 1) %>%
  dplyr::select(lopnr, date_month,shortterm = KORTTID) %>%
  distinct()

#calculate dates for NH at discharge
sabo_dates <- d %>%
  dplyr::select(lopnr,grp, in_date,out_date,admit,discharge) %>%
  left_join(sabo) %>%
  filter(date_month >= out_date - months(3)) %>%
  group_by(lopnr,grp) %>%
  filter(date_month == min(date_month)) %>%
  ungroup() %>%
  distinct() %>%
  mutate(sabo_diff = date_month - out_date)

#calculate dates for NH at admission
sabo_dates_in <- d %>%
  dplyr::select(lopnr,grp, in_date,out_date,admit,discharge) %>%
  left_join(sabo) %>%
  filter(date_month >= in_date - months(3)) %>%
  group_by(lopnr,grp) %>%
  filter(date_month == min(date_month)) %>%
  ungroup() %>%
  distinct() %>%
  mutate(sabo_diff_in = date_month - in_date)

# Get home service / home care dates
htj <- sol %>%
  filter(BOFORM <= 1 & KORTTID != 1,
         HTJ == 1) %>%
  dplyr::select(lopnr, date_month) %>%
  distinct()

hsl <- sol %>%
  filter(BOFORM <= 1 & KORTTID != 1,
         HSL == 1) %>%
  dplyr::select(lopnr, date_month) %>%
  distinct()
  
htj_dates <- d %>%
  dplyr::select(lopnr,grp, in_date,out_date,admit,discharge) %>%
  left_join(htj) %>%
  filter(date_month >= out_date - months(3)) %>%
  group_by(lopnr,grp) %>%
  filter(date_month == min(date_month)) %>%
  ungroup() %>%
  distinct() %>%
  mutate(htj_diff = date_month - out_date)

hsl_dates <- d %>%
  dplyr::select(lopnr,grp, in_date,out_date,admit,discharge) %>%
  left_join(hsl) %>%
  filter(date_month >= out_date - months(3)) %>%
  group_by(lopnr,grp) %>%
  filter(date_month == min(date_month)) %>%
  ungroup() %>%
  distinct() %>%
  mutate(hsl_diff = date_month - out_date)

d <- d %>%
  left_join(dplyr::select(sabo_dates,lopnr,grp,
                          sabo_date = date_month,
                          sabo_diff,shortterm),
            by=c("lopnr","grp")) %>%
  left_join(dplyr::select(sabo_dates_in,lopnr,grp,
                          sabo_date_in = date_month,
                          sabo_diff_in),
            by=c("lopnr","grp")) %>%
  left_join(dplyr::select(htj_dates,lopnr,grp,
                          htj_date = date_month,
                          htj_diff),
            by=c("lopnr","grp")) %>%
  left_join(dplyr::select(hsl_dates,lopnr,grp,
                          hsl_date = date_month,
                          hsl_diff),
            by=c("lopnr","grp"))


d <- d %>%
  #Exclude if admitted from home but has had NH care registered for at least 2 months before discharge (these patients are likely living in NH already)
  mutate(excl_admit = !(admit == "Ordinärt boende" & sabo_diff_in < -30 ) | is.na(sabo_diff),
  #Exclude if discharged to NH but no record of NH care for at least 2 months after discharge
  excl_discharge_sabo = !(discharge == "Särskilt boende" & (sabo_diff > 30 | is.na(sabo_diff))),
  #Exclude if discharged to home but record of NH care within 2 months
  excl_discharge_home = !(discharge == "Ordinärt boende" & (sabo_diff < 30 & !is.na(sabo_diff))),
  shortterm = ifelse(is.na(shortterm),0,shortterm)) %>%
  ungroup() %>%
  select(-dates,-unplanned_dates)

  


# Apply exclusion criteria, and save the number of included records at each step.
excl_n <- list()

excl_n$orig <- nrow(d)

#Exclude cases prior to 2016 (2015 used to calculate propensity score data)
d <- d %>%
 filter(year(out_date) > 2015)

excl_n$year_2015 <- nrow(d)

d <- d %>%
  filter(planned_contact == 0)

excl_n$unplanned <- nrow(d)

d <- d %>%
  arrange(lopnr,grp) %>%
  mutate(days_to_next_unplanned = ifelse(lead(lopnr) == lopnr,lead(in_date) - out_date,NA))

d <- d %>%
 filter(!is.na(source_discharge),
        !is.na(source_admit),
        !is.na(last_hosp))

excl_n$missingdata <- nrow(d)

d <- d %>%
  filter(!source_discharge %in% c(4,1))

excl_n$discharge <- nrow(d)

d <- d %>%
  filter(caredays > 2)

excl_n$shortstay <- nrow(d)

d <- d %>%
  filter(caredays <= max_caredays)

excl_n$longstay <- nrow(d)

d <- d %>%
  filter(out_date < ymd("20200101") - days(fu_days))

excl_n$endofstudy <- nrow(d)

d <- d %>%
  filter(!is.na(last_hosp_mvo))

excl_n$missingmvo <- nrow(d)

d <- d %>%
  filter((days_since_prev >= 0 | is.na(days_since_prev)) & 
           (out_days_to_next >= 0 | is.na(out_days_to_next)))

excl_n$shortgap <- nrow(d)

d <- d %>%
  filter(source_admit == 3)

excl_n$admitfromhome <- nrow(d)

if(sens_analysis != "noqualityexcl"){
  d <- d %>%
    filter(excl_admit & excl_discharge_sabo & excl_discharge_home)
}


excl_n$uncertain_admit_discharge <- nrow(d)

d <- d %>%
  filter((!is.na(htj_diff) & htj_diff < 30) | disch_sabo == 1)

excl_n$admit_home_no_care <- nrow(d)

d <- d %>%
  filter(n_diag > 1)

excl_n$multi_diag <- nrow(d)

if(sens_analysis != "noqualityexcl"){
  d <- d %>%
    group_by(lopnr) %>%
    mutate(first_disch_nh = min(out_date[disch_sabo == 1 & shortterm == 0],na.rm = T)) %>%
    ungroup() %>%
    filter(in_date < first_disch_nh)
}

excl_n$admitfromhome_after_nhdisch  <- nrow(d)

if(sens_analysis == "first_patient"){
  d <- d %>%
    group_by(lopnr) %>%
    filter(grp == min(grp))

}


excl_n$final <- nrow(d)

# Append home care volume data

htj_sum <- htj %>%
     arrange(lopnr, date_month) %>%
     group_by(lopnr) %>%
     mutate(n_htj = row_number()-1) %>%
  ungroup( )%>%
  select(lopnr, discharge_date_month = date_month, n_htj) %>%
  filter(lopnr %in% d$lopnr)

hsl_sum <- hsl %>%
     arrange(lopnr, date_month) %>%
     group_by(lopnr) %>%
     mutate(n_hc = row_number()-1) %>%
  ungroup() %>%
  select(lopnr, discharge_date_month =  date_month, n_hc) %>%
  filter(lopnr %in% d$lopnr)

muni_sums <- full_join(htj_sum,hsl_sum)

d <- d %>%
     left_join(muni_sums,
               by = c("lopnr","discharge_date_month")) %>%
  ungroup()

d$n_htj[is.na(d$n_htj)] <- 0
d$n_hc[is.na(d$n_hc)] <- 0

  save(d, file = "data_final.rda")
  save(excl_n, file = "excl_n.rda")
}
```

```
length(unique(d$lopnr))
```

```
## [1] 230815
```

```
excl_n_per_stage <- as.data.frame(t(as.data.frame(excl_n))) %>%
  transmute(sum = V1,
            excl = lag(sum)-sum,
            description = c("Patients over 65 with 2+ ICD codes", 
                            "Year > 2015", 
                            "Unplanned admission", 
                            "Missing discharge/admission/hospital data", 
                            "Discharge to home or NH", 
                            "Care duration < 3 days", 
                            "Care duration > 90 days", 
                            "Year < 2020", 
                            "Missing ward information", 
                            "Same day readmission", 
                            "Patient admitted from home", 
                            "Agreeement re: admission and discharge between data sources", 
                            "Discharged home without care services",
                            "Single diagnosis only",
                            "Admission from home discharged to NH following discharge to NH",
                            "Final"))


kable(excl_n_per_stage,row.names = F)
```

| sum | excl | description |
| --- | --- | --- |
| 2852592 | NA | Patients over 65 with 2+ ICD codes |
| 2289148 | 563444 | Year > 2015 |
| 1856728 | 432420 | Unplanned admission |
| 1856278 | 450 | Missing discharge/admission/hospital data |
| 1704954 | 151324 | Discharge to home or NH |
| 1376882 | 328072 | Care duration < 3 days |
| 1375519 | 1363 | Care duration > 90 days |
| 1288843 | 86676 | Year < 2020 |
| 1287612 | 1231 | Missing ward information |
| 1287108 | 504 | Same day readmission |
| 1188724 | 98384 | Patient admitted from home |
| 1001640 | 187084 | Agreeement re: admission and discharge between data sources |
| 434405 | 567235 | Discharged home without care services |
| 413657 | 20748 | Single diagnosis only |
| 413073 | 584 | Admission from home discharged to NH following discharge to NH |
| 230815 | 182258 | Final |

```
# Final n:
nrow(d)
```

```
## [1] 230815
```

```
# Generate sparse matrix for xgb


if(file.exists("./sm.rda") & !reload){
  load("./sm.rda")
}else{

  sm <- generate_sparsm(d)
  
  save(sm,file = "./sm.rda")
  
}
```

# Train propensity models

```
if(file.exists("./propensity_xgb.rda") & !reload){
  load("./propensity_xgb.rda")
  load("./propensity_xgb_cv.rda")
  load("./propensity_glm.rda")
  load("./propensity_glm_cv.rda")
}else{

  params <- list(objective = "binary:logistic")


  propensity_xgb_cv <- xgb.cv(data = xgb.DMatrix(data  = sm,
                                                 label = as.logical(d$disch_sabo)),
                             nfold = 5,
                             nrounds = 500,
                             prediction = T, 
                             params = params,
                             early_stopping_rounds = 10)

  propensity_xgb <- xgb.train(data = xgb.DMatrix(data  = sm,
                                                 label = as.logical(d$disch_sabo)),
                              params = params,
                            nrounds = propensity_xgb_cv$early_stop$best_iteration)
  

  
  propensity_glm_cv <- cv.glmnet(x=sm,
                                 y = as.logical(d$disch_sabo))
  
  propensity_glm <- glmnet(x=sm,
                           y = as.logical(d$disch_sabo),
                           lambda = propensity_glm_cv$lambda.min)
  
  
  
  save(propensity_xgb_cv,file = "./propensity_xgb_cv.rda")
  save(propensity_xgb,file = "./propensity_xgb.rda")
  save(propensity_glm_cv,file = "./propensity_glm_cv.rda")
  save(propensity_glm,file = "./propensity_glm.rda")
  
}
```

```
## Multiple eval metrics are present. Will use test_logloss for early stopping.
## Will train until test_logloss hasn't improved in 10 rounds.
## 
## [1]  train-logloss:0.465612±0.000518 test-logloss:0.466147±0.001737 
## [2]  train-logloss:0.435975±0.000662 test-logloss:0.436956±0.001432 
## [3]  train-logloss:0.416349±0.000827 test-logloss:0.417722±0.001287 
## [4]  train-logloss:0.402860±0.001096 test-logloss:0.404687±0.001209 
## [5]  train-logloss:0.392846±0.000998 test-logloss:0.395187±0.001348 
## [6]  train-logloss:0.385446±0.001159 test-logloss:0.388342±0.001308 
## [7]  train-logloss:0.379472±0.001211 test-logloss:0.382675±0.001266 
## [8]  train-logloss:0.374565±0.001226 test-logloss:0.378092±0.001412 
## [9]  train-logloss:0.370484±0.000846 test-logloss:0.374479±0.001809 
## [10] train-logloss:0.366886±0.000967 test-logloss:0.371201±0.001792 
## [11] train-logloss:0.363854±0.001140 test-logloss:0.368703±0.001667 
## [12] train-logloss:0.360733±0.000874 test-logloss:0.365991±0.001872 
## [13] train-logloss:0.357992±0.001173 test-logloss:0.363556±0.001725 
## [14] train-logloss:0.355773±0.001176 test-logloss:0.361728±0.001787 
## [15] train-logloss:0.353536±0.001030 test-logloss:0.359959±0.001984 
## [16] train-logloss:0.351447±0.000980 test-logloss:0.358350±0.002160 
## [17] train-logloss:0.349676±0.000907 test-logloss:0.357016±0.002260 
## [18] train-logloss:0.347969±0.000840 test-logloss:0.355597±0.002236 
## [19] train-logloss:0.346369±0.000682 test-logloss:0.354335±0.002364 
## [20] train-logloss:0.344866±0.000756 test-logloss:0.353197±0.002347 
## [21] train-logloss:0.343638±0.000688 test-logloss:0.352212±0.002423 
## [22] train-logloss:0.342460±0.000676 test-logloss:0.351419±0.002515 
## [23] train-logloss:0.340917±0.000765 test-logloss:0.350293±0.002352 
## [24] train-logloss:0.339720±0.000698 test-logloss:0.349445±0.002547 
## [25] train-logloss:0.338612±0.000728 test-logloss:0.348735±0.002525 
## [26] train-logloss:0.337511±0.000911 test-logloss:0.347918±0.002381 
## [27] train-logloss:0.336559±0.000879 test-logloss:0.347275±0.002382 
## [28] train-logloss:0.335378±0.000584 test-logloss:0.346437±0.002637 
## [29] train-logloss:0.334496±0.000700 test-logloss:0.345861±0.002569 
## [30] train-logloss:0.333603±0.000627 test-logloss:0.345340±0.002664 
## [31] train-logloss:0.332781±0.000603 test-logloss:0.344768±0.002678 
## [32] train-logloss:0.332006±0.000556 test-logloss:0.344213±0.002729 
## [33] train-logloss:0.331264±0.000477 test-logloss:0.343771±0.002804 
## [34] train-logloss:0.330328±0.000671 test-logloss:0.343179±0.002590 
## [35] train-logloss:0.329458±0.000900 test-logloss:0.342667±0.002506 
## [36] train-logloss:0.328791±0.000894 test-logloss:0.342289±0.002553 
## [37] train-logloss:0.328092±0.000902 test-logloss:0.341903±0.002469 
## [38] train-logloss:0.327393±0.000855 test-logloss:0.341551±0.002539 
## [39] train-logloss:0.326766±0.000842 test-logloss:0.341184±0.002577 
## [40] train-logloss:0.325928±0.000981 test-logloss:0.340666±0.002582 
## [41] train-logloss:0.325332±0.001000 test-logloss:0.340315±0.002594 
## [42] train-logloss:0.324696±0.000987 test-logloss:0.339933±0.002561 
## [43] train-logloss:0.324144±0.000948 test-logloss:0.339638±0.002526 
## [44] train-logloss:0.323527±0.000944 test-logloss:0.339257±0.002561 
## [45] train-logloss:0.322929±0.000939 test-logloss:0.338958±0.002491 
## [46] train-logloss:0.322271±0.000965 test-logloss:0.338626±0.002409 
## [47] train-logloss:0.321644±0.000941 test-logloss:0.338301±0.002471 
## [48] train-logloss:0.320952±0.000946 test-logloss:0.337977±0.002600 
## [49] train-logloss:0.320503±0.000965 test-logloss:0.337750±0.002608 
## [50] train-logloss:0.319906±0.000918 test-logloss:0.337422±0.002598 
## [51] train-logloss:0.319409±0.000916 test-logloss:0.337167±0.002605 
## [52] train-logloss:0.318881±0.000958 test-logloss:0.336895±0.002622 
## [53] train-logloss:0.318438±0.000998 test-logloss:0.336665±0.002538 
## [54] train-logloss:0.317960±0.000922 test-logloss:0.336434±0.002577 
## [55] train-logloss:0.317465±0.000828 test-logloss:0.336183±0.002553 
## [56] train-logloss:0.316912±0.000921 test-logloss:0.335971±0.002532 
## [57] train-logloss:0.316447±0.000807 test-logloss:0.335725±0.002516 
## [58] train-logloss:0.315997±0.000779 test-logloss:0.335460±0.002570 
## [59] train-logloss:0.315554±0.000701 test-logloss:0.335245±0.002617 
## [60] train-logloss:0.315119±0.000723 test-logloss:0.335050±0.002580 
## [61] train-logloss:0.314683±0.000732 test-logloss:0.334819±0.002571 
## [62] train-logloss:0.314234±0.000774 test-logloss:0.334622±0.002615 
## [63] train-logloss:0.313668±0.000915 test-logloss:0.334280±0.002679 
## [64] train-logloss:0.313258±0.000904 test-logloss:0.334119±0.002704 
## [65] train-logloss:0.312711±0.000795 test-logloss:0.333869±0.002760 
## [66] train-logloss:0.312351±0.000779 test-logloss:0.333705±0.002752 
## [67] train-logloss:0.311846±0.000811 test-logloss:0.333489±0.002631 
## [68] train-logloss:0.311443±0.000734 test-logloss:0.333279±0.002615 
## [69] train-logloss:0.311049±0.000702 test-logloss:0.333116±0.002670 
## [70] train-logloss:0.310648±0.000710 test-logloss:0.332953±0.002604 
## [71] train-logloss:0.310067±0.000665 test-logloss:0.332690±0.002528 
## [72] train-logloss:0.309643±0.000653 test-logloss:0.332502±0.002527 
## [73] train-logloss:0.309316±0.000646 test-logloss:0.332334±0.002540 
## [74] train-logloss:0.308913±0.000606 test-logloss:0.332172±0.002525 
## [75] train-logloss:0.308519±0.000595 test-logloss:0.332004±0.002532 
## [76] train-logloss:0.308018±0.000647 test-logloss:0.331735±0.002582 
## [77] train-logloss:0.307548±0.000702 test-logloss:0.331520±0.002509 
## [78] train-logloss:0.307161±0.000710 test-logloss:0.331370±0.002486 
## [79] train-logloss:0.306818±0.000668 test-logloss:0.331225±0.002452 
## [80] train-logloss:0.306478±0.000668 test-logloss:0.331115±0.002409 
## [81] train-logloss:0.306167±0.000657 test-logloss:0.331029±0.002434 
## [82] train-logloss:0.305782±0.000689 test-logloss:0.330864±0.002475 
## [83] train-logloss:0.305487±0.000613 test-logloss:0.330748±0.002469 
## [84] train-logloss:0.305148±0.000606 test-logloss:0.330630±0.002470 
## [85] train-logloss:0.304868±0.000592 test-logloss:0.330518±0.002447 
## [86] train-logloss:0.304524±0.000636 test-logloss:0.330396±0.002478 
## [87] train-logloss:0.304207±0.000682 test-logloss:0.330271±0.002446 
## [88] train-logloss:0.303878±0.000732 test-logloss:0.330192±0.002457 
## [89] train-logloss:0.303563±0.000741 test-logloss:0.330100±0.002493 
## [90] train-logloss:0.303200±0.000844 test-logloss:0.330000±0.002461 
## [91] train-logloss:0.302865±0.000875 test-logloss:0.329894±0.002460 
## [92] train-logloss:0.302570±0.000813 test-logloss:0.329758±0.002497 
## [93] train-logloss:0.302219±0.000730 test-logloss:0.329612±0.002520 
## [94] train-logloss:0.301852±0.000603 test-logloss:0.329497±0.002550 
## [95] train-logloss:0.301519±0.000611 test-logloss:0.329362±0.002519 
## [96] train-logloss:0.301179±0.000653 test-logloss:0.329280±0.002527 
## [97] train-logloss:0.300911±0.000684 test-logloss:0.329192±0.002528 
## [98] train-logloss:0.300577±0.000674 test-logloss:0.329061±0.002518 
## [99] train-logloss:0.300325±0.000697 test-logloss:0.328970±0.002496 
## [100]    train-logloss:0.300024±0.000736 test-logloss:0.328879±0.002492 
## [101]    train-logloss:0.299746±0.000746 test-logloss:0.328782±0.002506 
## [102]    train-logloss:0.299416±0.000713 test-logloss:0.328686±0.002578 
## [103]    train-logloss:0.299103±0.000648 test-logloss:0.328570±0.002616 
## [104]    train-logloss:0.298813±0.000616 test-logloss:0.328516±0.002575 
## [105]    train-logloss:0.298538±0.000633 test-logloss:0.328427±0.002572 
## [106]    train-logloss:0.298260±0.000641 test-logloss:0.328361±0.002592 
## [107]    train-logloss:0.297853±0.000669 test-logloss:0.328235±0.002483 
## [108]    train-logloss:0.297542±0.000739 test-logloss:0.328116±0.002431 
## [109]    train-logloss:0.297238±0.000707 test-logloss:0.328061±0.002477 
## [110]    train-logloss:0.297003±0.000700 test-logloss:0.328003±0.002512 
## [111]    train-logloss:0.296675±0.000705 test-logloss:0.327924±0.002431 
## [112]    train-logloss:0.296350±0.000707 test-logloss:0.327824±0.002462 
## [113]    train-logloss:0.296034±0.000784 test-logloss:0.327707±0.002452 
## [114]    train-logloss:0.295744±0.000725 test-logloss:0.327616±0.002406 
## [115]    train-logloss:0.295421±0.000665 test-logloss:0.327494±0.002431 
## [116]    train-logloss:0.295162±0.000676 test-logloss:0.327435±0.002488 
## [117]    train-logloss:0.294882±0.000675 test-logloss:0.327353±0.002497 
## [118]    train-logloss:0.294560±0.000761 test-logloss:0.327273±0.002510 
## [119]    train-logloss:0.294266±0.000825 test-logloss:0.327223±0.002513 
## [120]    train-logloss:0.294063±0.000810 test-logloss:0.327165±0.002521 
## [121]    train-logloss:0.293787±0.000775 test-logloss:0.327067±0.002616 
## [122]    train-logloss:0.293567±0.000789 test-logloss:0.327026±0.002590 
## [123]    train-logloss:0.293363±0.000800 test-logloss:0.326978±0.002627 
## [124]    train-logloss:0.293091±0.000796 test-logloss:0.326907±0.002600 
## [125]    train-logloss:0.292804±0.000814 test-logloss:0.326765±0.002444 
## [126]    train-logloss:0.292553±0.000840 test-logloss:0.326705±0.002385 
## [127]    train-logloss:0.292293±0.000819 test-logloss:0.326646±0.002375 
## [128]    train-logloss:0.292078±0.000846 test-logloss:0.326596±0.002364 
## [129]    train-logloss:0.291877±0.000823 test-logloss:0.326558±0.002355 
## [130]    train-logloss:0.291611±0.000854 test-logloss:0.326481±0.002365 
## [131]    train-logloss:0.291360±0.000844 test-logloss:0.326411±0.002378 
## [132]    train-logloss:0.291107±0.000944 test-logloss:0.326362±0.002344 
## [133]    train-logloss:0.290872±0.000911 test-logloss:0.326303±0.002365 
## [134]    train-logloss:0.290637±0.000903 test-logloss:0.326233±0.002350 
## [135]    train-logloss:0.290415±0.000926 test-logloss:0.326157±0.002395 
## [136]    train-logloss:0.290180±0.000929 test-logloss:0.326113±0.002386 
## [137]    train-logloss:0.289931±0.000947 test-logloss:0.326075±0.002402 
## [138]    train-logloss:0.289669±0.000942 test-logloss:0.326012±0.002408 
## [139]    train-logloss:0.289397±0.000933 test-logloss:0.325946±0.002416 
## [140]    train-logloss:0.289193±0.000931 test-logloss:0.325889±0.002409 
## [141]    train-logloss:0.288995±0.000885 test-logloss:0.325805±0.002485 
## [142]    train-logloss:0.288821±0.000859 test-logloss:0.325756±0.002511 
## [143]    train-logloss:0.288589±0.000828 test-logloss:0.325723±0.002473 
## [144]    train-logloss:0.288419±0.000848 test-logloss:0.325669±0.002445 
## [145]    train-logloss:0.288232±0.000829 test-logloss:0.325636±0.002410 
## [146]    train-logloss:0.288057±0.000806 test-logloss:0.325591±0.002374 
## [147]    train-logloss:0.287813±0.000808 test-logloss:0.325571±0.002359 
## [148]    train-logloss:0.287482±0.000717 test-logloss:0.325467±0.002374 
## [149]    train-logloss:0.287177±0.000710 test-logloss:0.325398±0.002301 
## [150]    train-logloss:0.287005±0.000707 test-logloss:0.325360±0.002297 
## [151]    train-logloss:0.286777±0.000687 test-logloss:0.325345±0.002336 
## [152]    train-logloss:0.286528±0.000637 test-logloss:0.325292±0.002314 
## [153]    train-logloss:0.286176±0.000742 test-logloss:0.325204±0.002283 
## [154]    train-logloss:0.285894±0.000845 test-logloss:0.325148±0.002259 
## [155]    train-logloss:0.285670±0.000835 test-logloss:0.325093±0.002249 
## [156]    train-logloss:0.285435±0.000835 test-logloss:0.325044±0.002197 
## [157]    train-logloss:0.285230±0.000854 test-logloss:0.325003±0.002164 
## [158]    train-logloss:0.285086±0.000854 test-logloss:0.324972±0.002160 
## [159]    train-logloss:0.284877±0.000860 test-logloss:0.324914±0.002156 
## [160]    train-logloss:0.284642±0.000852 test-logloss:0.324864±0.002146 
## [161]    train-logloss:0.284403±0.000864 test-logloss:0.324852±0.002148 
## [162]    train-logloss:0.284210±0.000883 test-logloss:0.324804±0.002141 
## [163]    train-logloss:0.284049±0.000887 test-logloss:0.324775±0.002128 
## [164]    train-logloss:0.283843±0.000868 test-logloss:0.324717±0.002130 
## [165]    train-logloss:0.283662±0.000899 test-logloss:0.324688±0.002138 
## [166]    train-logloss:0.283451±0.000917 test-logloss:0.324669±0.002123 
## [167]    train-logloss:0.283239±0.000948 test-logloss:0.324627±0.002100 
## [168]    train-logloss:0.283025±0.000932 test-logloss:0.324588±0.002123 
## [169]    train-logloss:0.282844±0.000942 test-logloss:0.324556±0.002112 
## [170]    train-logloss:0.282684±0.000968 test-logloss:0.324538±0.002117 
## [171]    train-logloss:0.282514±0.000999 test-logloss:0.324531±0.002130 
## [172]    train-logloss:0.282228±0.000989 test-logloss:0.324443±0.002136 
## [173]    train-logloss:0.281973±0.000966 test-logloss:0.324359±0.002160 
## [174]    train-logloss:0.281724±0.001059 test-logloss:0.324353±0.002173 
## [175]    train-logloss:0.281527±0.001111 test-logloss:0.324347±0.002197 
## [176]    train-logloss:0.281343±0.001103 test-logloss:0.324318±0.002204 
## [177]    train-logloss:0.281167±0.001086 test-logloss:0.324267±0.002233 
## [178]    train-logloss:0.281041±0.001064 test-logloss:0.324233±0.002241 
## [179]    train-logloss:0.280933±0.001059 test-logloss:0.324219±0.002246 
## [180]    train-logloss:0.280704±0.000968 test-logloss:0.324173±0.002283 
## [181]    train-logloss:0.280531±0.000923 test-logloss:0.324159±0.002294 
## [182]    train-logloss:0.280372±0.000917 test-logloss:0.324095±0.002298 
## [183]    train-logloss:0.280175±0.000903 test-logloss:0.324055±0.002293 
## [184]    train-logloss:0.279998±0.000927 test-logloss:0.324004±0.002299 
## [185]    train-logloss:0.279814±0.000989 test-logloss:0.323961±0.002278 
## [186]    train-logloss:0.279635±0.001043 test-logloss:0.323908±0.002265 
## [187]    train-logloss:0.279463±0.001097 test-logloss:0.323886±0.002259 
## [188]    train-logloss:0.279231±0.001192 test-logloss:0.323830±0.002231 
## [189]    train-logloss:0.279017±0.001214 test-logloss:0.323806±0.002226 
## [190]    train-logloss:0.278845±0.001284 test-logloss:0.323781±0.002221 
## [191]    train-logloss:0.278722±0.001293 test-logloss:0.323757±0.002229 
## [192]    train-logloss:0.278584±0.001284 test-logloss:0.323742±0.002253 
## [193]    train-logloss:0.278413±0.001275 test-logloss:0.323716±0.002245 
## [194]    train-logloss:0.278257±0.001291 test-logloss:0.323701±0.002239 
## [195]    train-logloss:0.278105±0.001291 test-logloss:0.323670±0.002265 
## [196]    train-logloss:0.277944±0.001290 test-logloss:0.323659±0.002246 
## [197]    train-logloss:0.277815±0.001285 test-logloss:0.323638±0.002256 
## [198]    train-logloss:0.277612±0.001281 test-logloss:0.323606±0.002277 
## [199]    train-logloss:0.277476±0.001317 test-logloss:0.323583±0.002266 
## [200]    train-logloss:0.277277±0.001267 test-logloss:0.323559±0.002287 
## [201]    train-logloss:0.277030±0.001218 test-logloss:0.323490±0.002277 
## [202]    train-logloss:0.276867±0.001195 test-logloss:0.323471±0.002287 
## [203]    train-logloss:0.276737±0.001178 test-logloss:0.323461±0.002309 
## [204]    train-logloss:0.276594±0.001174 test-logloss:0.323439±0.002318 
## [205]    train-logloss:0.276413±0.001201 test-logloss:0.323420±0.002313 
## [206]    train-logloss:0.276271±0.001182 test-logloss:0.323407±0.002305 
## [207]    train-logloss:0.276045±0.001115 test-logloss:0.323349±0.002343 
## [208]    train-logloss:0.275864±0.001062 test-logloss:0.323340±0.002345 
## [209]    train-logloss:0.275728±0.001098 test-logloss:0.323320±0.002335 
## [210]    train-logloss:0.275607±0.001144 test-logloss:0.323316±0.002340 
## [211]    train-logloss:0.275401±0.001204 test-logloss:0.323289±0.002323 
## [212]    train-logloss:0.275214±0.001240 test-logloss:0.323281±0.002322 
## [213]    train-logloss:0.275027±0.001256 test-logloss:0.323236±0.002312 
## [214]    train-logloss:0.274926±0.001278 test-logloss:0.323241±0.002330 
## [215]    train-logloss:0.274783±0.001239 test-logloss:0.323224±0.002342 
## [216]    train-logloss:0.274633±0.001224 test-logloss:0.323209±0.002340 
## [217]    train-logloss:0.274495±0.001214 test-logloss:0.323164±0.002329 
## [218]    train-logloss:0.274298±0.001202 test-logloss:0.323145±0.002321 
## [219]    train-logloss:0.274164±0.001218 test-logloss:0.323136±0.002324 
## [220]    train-logloss:0.274011±0.001222 test-logloss:0.323152±0.002303 
## [221]    train-logloss:0.273841±0.001177 test-logloss:0.323149±0.002340 
## [222]    train-logloss:0.273695±0.001150 test-logloss:0.323137±0.002364 
## [223]    train-logloss:0.273579±0.001162 test-logloss:0.323132±0.002367 
## [224]    train-logloss:0.273405±0.001128 test-logloss:0.323132±0.002340 
## [225]    train-logloss:0.273242±0.001075 test-logloss:0.323109±0.002334 
## [226]    train-logloss:0.272976±0.001105 test-logloss:0.323050±0.002270 
## [227]    train-logloss:0.272842±0.001097 test-logloss:0.323015±0.002277 
## [228]    train-logloss:0.272643±0.001048 test-logloss:0.322997±0.002331 
## [229]    train-logloss:0.272516±0.001025 test-logloss:0.322975±0.002328 
## [230]    train-logloss:0.272387±0.001031 test-logloss:0.322954±0.002299 
## [231]    train-logloss:0.272192±0.001058 test-logloss:0.322967±0.002285 
## [232]    train-logloss:0.272061±0.001069 test-logloss:0.322980±0.002277 
## [233]    train-logloss:0.271876±0.001126 test-logloss:0.322960±0.002306 
## [234]    train-logloss:0.271663±0.001187 test-logloss:0.322897±0.002285 
## [235]    train-logloss:0.271542±0.001183 test-logloss:0.322863±0.002281 
## [236]    train-logloss:0.271413±0.001193 test-logloss:0.322865±0.002283 
## [237]    train-logloss:0.271288±0.001193 test-logloss:0.322831±0.002284 
## [238]    train-logloss:0.271150±0.001175 test-logloss:0.322828±0.002305 
## [239]    train-logloss:0.270930±0.001232 test-logloss:0.322787±0.002283 
## [240]    train-logloss:0.270751±0.001250 test-logloss:0.322751±0.002269 
## [241]    train-logloss:0.270599±0.001224 test-logloss:0.322728±0.002269 
## [242]    train-logloss:0.270469±0.001216 test-logloss:0.322739±0.002273 
## [243]    train-logloss:0.270338±0.001226 test-logloss:0.322739±0.002278 
## [244]    train-logloss:0.270195±0.001269 test-logloss:0.322714±0.002220 
## [245]    train-logloss:0.270046±0.001290 test-logloss:0.322697±0.002209 
## [246]    train-logloss:0.269925±0.001290 test-logloss:0.322689±0.002223 
## [247]    train-logloss:0.269798±0.001296 test-logloss:0.322670±0.002232 
## [248]    train-logloss:0.269666±0.001320 test-logloss:0.322662±0.002214 
## [249]    train-logloss:0.269575±0.001335 test-logloss:0.322649±0.002230 
## [250]    train-logloss:0.269424±0.001344 test-logloss:0.322660±0.002220 
## [251]    train-logloss:0.269244±0.001265 test-logloss:0.322652±0.002206 
## [252]    train-logloss:0.269103±0.001195 test-logloss:0.322627±0.002212 
## [253]    train-logloss:0.268959±0.001166 test-logloss:0.322590±0.002230 
## [254]    train-logloss:0.268826±0.001175 test-logloss:0.322570±0.002210 
## [255]    train-logloss:0.268700±0.001224 test-logloss:0.322565±0.002212 
## [256]    train-logloss:0.268597±0.001252 test-logloss:0.322570±0.002212 
## [257]    train-logloss:0.268479±0.001227 test-logloss:0.322539±0.002211 
## [258]    train-logloss:0.268327±0.001155 test-logloss:0.322536±0.002211 
## [259]    train-logloss:0.268187±0.001149 test-logloss:0.322515±0.002208 
## [260]    train-logloss:0.268029±0.001164 test-logloss:0.322463±0.002213 
## [261]    train-logloss:0.267853±0.001136 test-logloss:0.322445±0.002198 
## [262]    train-logloss:0.267732±0.001177 test-logloss:0.322420±0.002212 
## [263]    train-logloss:0.267552±0.001245 test-logloss:0.322376±0.002192 
## [264]    train-logloss:0.267382±0.001221 test-logloss:0.322347±0.002173 
## [265]    train-logloss:0.267194±0.001120 test-logloss:0.322362±0.002148 
## [266]    train-logloss:0.267032±0.001103 test-logloss:0.322352±0.002123 
## [267]    train-logloss:0.266866±0.001063 test-logloss:0.322285±0.002158 
## [268]    train-logloss:0.266701±0.001045 test-logloss:0.322286±0.002168 
## [269]    train-logloss:0.266595±0.001085 test-logloss:0.322287±0.002165 
## [270]    train-logloss:0.266513±0.001084 test-logloss:0.322266±0.002164 
## [271]    train-logloss:0.266349±0.001089 test-logloss:0.322264±0.002157 
## [272]    train-logloss:0.266232±0.001133 test-logloss:0.322267±0.002177 
## [273]    train-logloss:0.266121±0.001107 test-logloss:0.322256±0.002198 
## [274]    train-logloss:0.265922±0.001066 test-logloss:0.322241±0.002195 
## [275]    train-logloss:0.265785±0.001137 test-logloss:0.322210±0.002179 
## [276]    train-logloss:0.265696±0.001136 test-logloss:0.322198±0.002196 
## [277]    train-logloss:0.265582±0.001129 test-logloss:0.322185±0.002181 
## [278]    train-logloss:0.265394±0.001144 test-logloss:0.322105±0.002187 
## [279]    train-logloss:0.265240±0.001144 test-logloss:0.322068±0.002192 
## [280]    train-logloss:0.265104±0.001146 test-logloss:0.322061±0.002196 
## [281]    train-logloss:0.264909±0.001130 test-logloss:0.322036±0.002212 
## [282]    train-logloss:0.264713±0.001171 test-logloss:0.322010±0.002189 
## [283]    train-logloss:0.264535±0.001162 test-logloss:0.322009±0.002168 
## [284]    train-logloss:0.264409±0.001190 test-logloss:0.321976±0.002122 
## [285]    train-logloss:0.264283±0.001180 test-logloss:0.321948±0.002114 
## [286]    train-logloss:0.264113±0.001199 test-logloss:0.321949±0.002095 
## [287]    train-logloss:0.263990±0.001226 test-logloss:0.321953±0.002111 
## [288]    train-logloss:0.263862±0.001260 test-logloss:0.321954±0.002071 
## [289]    train-logloss:0.263746±0.001292 test-logloss:0.321963±0.002093 
## [290]    train-logloss:0.263650±0.001313 test-logloss:0.321965±0.002100 
## [291]    train-logloss:0.263543±0.001353 test-logloss:0.321950±0.002110 
## [292]    train-logloss:0.263475±0.001340 test-logloss:0.321938±0.002100 
## [293]    train-logloss:0.263385±0.001359 test-logloss:0.321931±0.002102 
## [294]    train-logloss:0.263276±0.001369 test-logloss:0.321939±0.002108 
## [295]    train-logloss:0.263173±0.001352 test-logloss:0.321917±0.002115 
## [296]    train-logloss:0.263093±0.001353 test-logloss:0.321916±0.002107 
## [297]    train-logloss:0.262949±0.001366 test-logloss:0.321913±0.002108 
## [298]    train-logloss:0.262828±0.001323 test-logloss:0.321923±0.002115 
## [299]    train-logloss:0.262641±0.001331 test-logloss:0.321892±0.002162 
## [300]    train-logloss:0.262508±0.001238 test-logloss:0.321888±0.002162 
## [301]    train-logloss:0.262316±0.001153 test-logloss:0.321846±0.002224 
## [302]    train-logloss:0.262156±0.001140 test-logloss:0.321833±0.002235 
## [303]    train-logloss:0.262007±0.001052 test-logloss:0.321801±0.002230 
## [304]    train-logloss:0.261882±0.001066 test-logloss:0.321778±0.002225 
## [305]    train-logloss:0.261753±0.001022 test-logloss:0.321777±0.002237 
## [306]    train-logloss:0.261619±0.000944 test-logloss:0.321781±0.002251 
## [307]    train-logloss:0.261474±0.001012 test-logloss:0.321744±0.002242 
## [308]    train-logloss:0.261333±0.001020 test-logloss:0.321720±0.002241 
## [309]    train-logloss:0.261184±0.001037 test-logloss:0.321733±0.002255 
## [310]    train-logloss:0.261010±0.001131 test-logloss:0.321720±0.002237 
## [311]    train-logloss:0.260885±0.001200 test-logloss:0.321686±0.002251 
## [312]    train-logloss:0.260775±0.001222 test-logloss:0.321669±0.002244 
## [313]    train-logloss:0.260661±0.001279 test-logloss:0.321653±0.002226 
## [314]    train-logloss:0.260545±0.001327 test-logloss:0.321637±0.002242 
## [315]    train-logloss:0.260417±0.001388 test-logloss:0.321640±0.002269 
## [316]    train-logloss:0.260304±0.001418 test-logloss:0.321647±0.002274 
## [317]    train-logloss:0.260200±0.001428 test-logloss:0.321640±0.002269 
## [318]    train-logloss:0.260088±0.001427 test-logloss:0.321627±0.002272 
## [319]    train-logloss:0.259953±0.001439 test-logloss:0.321598±0.002286 
## [320]    train-logloss:0.259851±0.001432 test-logloss:0.321602±0.002310 
## [321]    train-logloss:0.259763±0.001424 test-logloss:0.321599±0.002308 
## [322]    train-logloss:0.259646±0.001394 test-logloss:0.321614±0.002266 
## [323]    train-logloss:0.259528±0.001351 test-logloss:0.321608±0.002281 
## [324]    train-logloss:0.259429±0.001357 test-logloss:0.321603±0.002269 
## [325]    train-logloss:0.259318±0.001349 test-logloss:0.321584±0.002253 
## [326]    train-logloss:0.259242±0.001351 test-logloss:0.321577±0.002256 
## [327]    train-logloss:0.259146±0.001333 test-logloss:0.321588±0.002261 
## [328]    train-logloss:0.259032±0.001324 test-logloss:0.321581±0.002289 
## [329]    train-logloss:0.258935±0.001342 test-logloss:0.321566±0.002274 
## [330]    train-logloss:0.258837±0.001322 test-logloss:0.321530±0.002269 
## [331]    train-logloss:0.258754±0.001304 test-logloss:0.321532±0.002271 
## [332]    train-logloss:0.258655±0.001298 test-logloss:0.321512±0.002282 
## [333]    train-logloss:0.258464±0.001387 test-logloss:0.321506±0.002270 
## [334]    train-logloss:0.258375±0.001375 test-logloss:0.321490±0.002263 
## [335]    train-logloss:0.258253±0.001437 test-logloss:0.321488±0.002266 
## [336]    train-logloss:0.258142±0.001425 test-logloss:0.321478±0.002230 
## [337]    train-logloss:0.258061±0.001419 test-logloss:0.321475±0.002253 
## [338]    train-logloss:0.257942±0.001431 test-logloss:0.321468±0.002268 
## [339]    train-logloss:0.257841±0.001402 test-logloss:0.321472±0.002266 
## [340]    train-logloss:0.257716±0.001358 test-logloss:0.321453±0.002259 
## [341]    train-logloss:0.257599±0.001297 test-logloss:0.321484±0.002241 
## [342]    train-logloss:0.257499±0.001313 test-logloss:0.321473±0.002219 
## [343]    train-logloss:0.257330±0.001322 test-logloss:0.321469±0.002265 
## [344]    train-logloss:0.257222±0.001354 test-logloss:0.321464±0.002256 
## [345]    train-logloss:0.257119±0.001397 test-logloss:0.321463±0.002262 
## [346]    train-logloss:0.256950±0.001522 test-logloss:0.321414±0.002263 
## [347]    train-logloss:0.256827±0.001535 test-logloss:0.321411±0.002256 
## [348]    train-logloss:0.256687±0.001565 test-logloss:0.321397±0.002260 
## [349]    train-logloss:0.256574±0.001558 test-logloss:0.321372±0.002275 
## [350]    train-logloss:0.256436±0.001544 test-logloss:0.321342±0.002257 
## [351]    train-logloss:0.256301±0.001566 test-logloss:0.321317±0.002184 
## [352]    train-logloss:0.256178±0.001550 test-logloss:0.321305±0.002193 
## [353]    train-logloss:0.256085±0.001553 test-logloss:0.321305±0.002191 
## [354]    train-logloss:0.256010±0.001544 test-logloss:0.321307±0.002181 
## [355]    train-logloss:0.255913±0.001537 test-logloss:0.321319±0.002199 
## [356]    train-logloss:0.255849±0.001560 test-logloss:0.321324±0.002194 
## [357]    train-logloss:0.255745±0.001568 test-logloss:0.321312±0.002194 
## [358]    train-logloss:0.255610±0.001594 test-logloss:0.321315±0.002229 
## [359]    train-logloss:0.255471±0.001597 test-logloss:0.321306±0.002264 
## [360]    train-logloss:0.255356±0.001591 test-logloss:0.321280±0.002288 
## [361]    train-logloss:0.255291±0.001577 test-logloss:0.321277±0.002304 
## [362]    train-logloss:0.255185±0.001600 test-logloss:0.321273±0.002317 
## [363]    train-logloss:0.255083±0.001606 test-logloss:0.321265±0.002309 
## [364]    train-logloss:0.255017±0.001613 test-logloss:0.321241±0.002316 
## [365]    train-logloss:0.254855±0.001632 test-logloss:0.321228±0.002291 
## [366]    train-logloss:0.254734±0.001560 test-logloss:0.321206±0.002314 
## [367]    train-logloss:0.254549±0.001461 test-logloss:0.321184±0.002354 
## [368]    train-logloss:0.254492±0.001443 test-logloss:0.321187±0.002338 
## [369]    train-logloss:0.254369±0.001380 test-logloss:0.321188±0.002342 
## [370]    train-logloss:0.254288±0.001359 test-logloss:0.321168±0.002339 
## [371]    train-logloss:0.254129±0.001323 test-logloss:0.321152±0.002362 
## [372]    train-logloss:0.254034±0.001309 test-logloss:0.321130±0.002362 
## [373]    train-logloss:0.253904±0.001261 test-logloss:0.321124±0.002368 
## [374]    train-logloss:0.253794±0.001236 test-logloss:0.321119±0.002377 
## [375]    train-logloss:0.253698±0.001247 test-logloss:0.321106±0.002385 
## [376]    train-logloss:0.253560±0.001227 test-logloss:0.321092±0.002370 
## [377]    train-logloss:0.253416±0.001215 test-logloss:0.321068±0.002373 
## [378]    train-logloss:0.253332±0.001225 test-logloss:0.321050±0.002360 
## [379]    train-logloss:0.253243±0.001220 test-logloss:0.321025±0.002390 
## [380]    train-logloss:0.253103±0.001307 test-logloss:0.321001±0.002376 
## [381]    train-logloss:0.253019±0.001320 test-logloss:0.320998±0.002361 
## [382]    train-logloss:0.252910±0.001310 test-logloss:0.321001±0.002333 
## [383]    train-logloss:0.252801±0.001320 test-logloss:0.320980±0.002324 
## [384]    train-logloss:0.252706±0.001317 test-logloss:0.320971±0.002320 
## [385]    train-logloss:0.252577±0.001338 test-logloss:0.320960±0.002309 
## [386]    train-logloss:0.252403±0.001296 test-logloss:0.320944±0.002302 
## [387]    train-logloss:0.252338±0.001292 test-logloss:0.320963±0.002300 
## [388]    train-logloss:0.252254±0.001344 test-logloss:0.320965±0.002305 
## [389]    train-logloss:0.252183±0.001347 test-logloss:0.320974±0.002306 
## [390]    train-logloss:0.252113±0.001360 test-logloss:0.320958±0.002307 
## [391]    train-logloss:0.251981±0.001322 test-logloss:0.320944±0.002307 
## [392]    train-logloss:0.251860±0.001307 test-logloss:0.320936±0.002300 
## [393]    train-logloss:0.251768±0.001322 test-logloss:0.320948±0.002305 
## [394]    train-logloss:0.251615±0.001242 test-logloss:0.320939±0.002337 
## [395]    train-logloss:0.251487±0.001247 test-logloss:0.320919±0.002331 
## [396]    train-logloss:0.251382±0.001240 test-logloss:0.320930±0.002347 
## [397]    train-logloss:0.251312±0.001237 test-logloss:0.320930±0.002365 
## [398]    train-logloss:0.251239±0.001253 test-logloss:0.320938±0.002353 
## [399]    train-logloss:0.251195±0.001243 test-logloss:0.320937±0.002356 
## [400]    train-logloss:0.251094±0.001219 test-logloss:0.320907±0.002349 
## [401]    train-logloss:0.250984±0.001240 test-logloss:0.320908±0.002360 
## [402]    train-logloss:0.250923±0.001248 test-logloss:0.320903±0.002357 
## [403]    train-logloss:0.250791±0.001223 test-logloss:0.320914±0.002353 
## [404]    train-logloss:0.250696±0.001218 test-logloss:0.320906±0.002353 
## [405]    train-logloss:0.250603±0.001211 test-logloss:0.320895±0.002341 
## [406]    train-logloss:0.250476±0.001182 test-logloss:0.320897±0.002398 
## [407]    train-logloss:0.250376±0.001155 test-logloss:0.320916±0.002396 
## [408]    train-logloss:0.250278±0.001157 test-logloss:0.320920±0.002371 
## [409]    train-logloss:0.250173±0.001139 test-logloss:0.320940±0.002385 
## [410]    train-logloss:0.250072±0.001105 test-logloss:0.320959±0.002401 
## [411]    train-logloss:0.250016±0.001102 test-logloss:0.320950±0.002396 
## [412]    train-logloss:0.249959±0.001093 test-logloss:0.320945±0.002388 
## [413]    train-logloss:0.249862±0.001115 test-logloss:0.320961±0.002374 
## [414]    train-logloss:0.249725±0.001171 test-logloss:0.320970±0.002373 
## Stopping. Best iteration:
## [415]    train-logloss:0.249650±0.001187 test-logloss:0.320976±0.002388
## 
## [415]    train-logloss:0.249650±0.001187 test-logloss:0.320976±0.002388
```

```
d_eval <- d %>% 
  ungroup() %>%
  dplyr::select(id,
                lopnr,
                out_date,
                age,
                gender,
                region,
                muni,
                caredays,
                planned_contact,
                mdc,
                mvo_last,
                n_op,
                n_diag,
                disch_sabo,
                days_since_prev,
                count_yr,
                count_yr_unplanned,
                count_yr_ov_planned,
                count_yr_ov_unplanned,
                unplanreadmit30,
                mort30,
                days_to_next_unplanned,
                mort_days,
                sabo_diff,
                n_hc,
                n_htj) %>% #include months of HC before hospitalization 
  mutate(nh_propensity_xgb = propensity_xgb_cv$cv_predict$pred,
         nh_propensity_glm = predict(propensity_glm,newx = sm),
         nh_propensity = nh_propensity_xgb,
         mortorreadmit30 = pmax(unplanreadmit30,mort30),
         days_since_prev_fill = ifelse(is.na(days_since_prev),366,days_since_prev),
         prop_weight = disch_sabo/nh_propensity + (1-disch_sabo)/(1-nh_propensity),
         prop_strat = cut(nh_propensity,quantile(nh_propensity,probs = seq(0, 1, 0.1)),include.lowest = T),
         ts_days_to_next_unplanned = ifelse(days_to_next_unplanned>=0,days_to_next_unplanned,NA),
         ts_days_to_sabo = ifelse(sabo_diff>=0 & disch_sabo == 0,sabo_diff,NA),
         ts_days_to_mort = ifelse(mort_days>=0,mort_days,NA),
         ts_days_to_any = pmin(ts_days_to_next_unplanned,ts_days_to_mort,na.rm = T),
         
         ts_mort_days_pp = pmin(ts_days_to_mort,
                             ts_days_to_sabo,
                             fu_days,na.rm = T),
         ts_mort_event_pp = ts_days_to_mort == ts_mort_days_pp & !is.na(ts_days_to_mort),
         
         
         ts_mort_days_itt = pmin(ts_days_to_mort,
                             fu_days,na.rm = T),
         ts_mort_event_itt = ts_days_to_mort == ts_mort_days_itt & !is.na(ts_days_to_mort),
         
         ts_any_days_itt = pmin(ts_days_to_any,
                             fu_days,na.rm = T),
         
         ts_any_event_itt = ts_days_to_any == ts_any_days_itt & !is.na(ts_days_to_any),
         
         ts_readmit_days_pp = pmin(ts_days_to_next_unplanned,
                             ts_days_to_mort,
                             ts_days_to_sabo,
                             fu_days,na.rm = T),
         ts_readmit_event_pp = as.factor(ifelse(ts_readmit_days_pp == ts_days_to_next_unplanned & 
                                 !is.na(ts_days_to_next_unplanned), "Readmission",
                               ifelse(ts_readmit_days_pp == ts_days_to_mort & 
                                 !is.na(ts_days_to_mort), "Death","Censored"))),
         
         ts_readmit_days_itt = pmin(ts_days_to_next_unplanned,
                             ts_days_to_mort,
                             fu_days,na.rm = T),
         
         ts_readmit_event_itt = factor(ifelse(ts_readmit_days_itt == ts_days_to_next_unplanned & 
                                         !is.na(ts_days_to_next_unplanned), "Readmission",
                                       ifelse(ts_readmit_days_itt == ts_days_to_mort & 
                                         !is.na(ts_days_to_mort), "Death","Censored")),
                                       levels = c("Censored","Readmission","Death")),
         
         ts_readmit_days_itt_se = pmin(ts_days_to_next_unplanned,
                             ts_days_to_mort,
                             fu_days,na.rm = T),
         ts_readmit_event_itt_se = ts_readmit_days_itt == ts_days_to_next_unplanned &
           !is.na(ts_days_to_next_unplanned),
         ts_mort_days_itt_7 = pmin(ts_mort_days_itt, 7),
         ts_mort_event_itt_7 = ifelse(ts_mort_days_itt <= 7 & ts_mort_event_itt, T, F),
         
         ts_mort_days_itt_30 = pmin(ts_mort_days_itt, 30),
         ts_mort_event_itt_30 = ifelse(ts_mort_days_itt <= 30 & ts_mort_event_itt, T, F),
         
         ts_readmit_days_itt_7 = pmin(ts_readmit_days_itt, 7),
         ts_readmit_event_itt_7 = factor(ifelse(ts_readmit_days_itt <= 7, 
                                                 as.character(ts_readmit_event_itt),
                                                 "Censored"),levels = c("Censored",
                                                                        "Readmission",
                                                                        "Death")),
         
         ts_readmit_days_itt_30 = pmin(ts_readmit_days_itt, 30),
         ts_readmit_event_itt_30 = factor(ifelse(ts_readmit_days_itt <= 30, 
                                                 as.character(ts_readmit_event_itt),
                                                 "Censored"),levels = c("Censored",
                                                                        "Readmission",
                                                                        "Death")),
         ts_any_days_itt_7 = pmin(ts_any_days_itt, 7),
         ts_any_event_itt_7 = ifelse(ts_any_days_itt <= 7 & ts_any_event_itt, T, F),
         
         ts_any_days_itt_30 = pmin(ts_any_days_itt, 30),
         ts_any_event_itt_30 = ifelse(ts_any_days_itt <= 30 & ts_any_event_itt, T, F),
         )

if(sens_analysis == "glm_propensity") {
  d_eval$nh_propensity <- d_eval$nh_propensity_glm
}
```

# Perform matching

```
if(file.exists("./m_exact.rda") & !reload){
  #load("./m_full.rda")
  #load("./m_caliper.rda")
  load("./m_exact.rda")
}else{
  
  pct_99 = quantile(d_eval$nh_propensity[d_eval$disch_sabo==0],probs = 0.99)
  
  # Force matchit to use all NH discharges by preventing it from selecting NH patients as controls
  m_d_eval <- d_eval %>% 
    group_by(lopnr) %>%
    filter(disch_sabo == 1 | !(any(disch_sabo == 1) & disch_sabo == 0),
           !(nh_propensity > pct_99 & disch_sabo == 1)) # remove NH discharges above 99th percentile of non-NH discharged propensities to ensure even post matching propensity score distribution
    
  
  # m_ind_full <- matchit(disch_sabo ~ nh_propensity, 
  #              data = m_d_eval, 
  #              method="nearest",
  #              ratio=1,
  #              unit.id = "lopnr" #this argument ensures that only one episode is used 
  #              )
  # 
  # m_ind_caliper <- matchit(disch_sabo ~ nh_propensity, 
  #              data = m_d_eval, 
  #              method="nearest",
  #              caliper = 0.05,
  #              ratio=1,
  #              unit.id = "lopnr"
  #              )
  
  m_ind_exact <- matchit(disch_sabo ~ nh_propensity, 
               data = m_d_eval, 
               method="nearest",
               caliper = 0.05,
               exact = ~ gender + mdc,
               ratio=1,
               unit.id = "lopnr" #this argument ensures that only one episode is used 
               )
  
  # save(m_ind_full,file = "./m_full.rda")
  # save(m_ind_caliper,file = "./m_caliper.rda")
  save(m_ind_exact,file = "./m_exact.rda")
  
}

#m_data_full <- match.data(m_ind_full)
#m_data_caliper <- match.data(m_ind_caliper)
load("./m_exact.rda")
m_data <- match.data(m_ind_exact)
```

# Propensity score validation

## mean values by percentile

```
m_data <- m_data %>%
  ungroup() %>%
  mutate(prop_strat_match = cut(nh_propensity,
                                quantile(nh_propensity,
                                         probs = seq(0, 1, 0.1)),
                                include.lowest = T))

d_strat <- m_data %>%
  group_by(prop_strat_match,disch_sabo) %>%
  summarise(mean_age = mean(age),
            pct_female = mean(gender),
            mean_caredays = mean(caredays),
            mean_n_op = mean(n_op),
            mean_n_diag = mean(n_diag),
            mean_days_since_prev = mean(days_since_prev,na.rm=T),
            mean_count_ov_unplan = mean(count_yr_ov_unplanned),
            mean_count_yr = mean(count_yr),
            mean_count_yr_unplan = mean(count_yr_unplanned),
            pct_unplanreadmit30 = mean(unplanreadmit30),
            pct_mort30 = mean(mort30),
            mean_hc = mean(n_hc),
            mean_htj = mean(n_htj),
            n = n()) %>%
  pivot_longer(cols = -c(prop_strat_match,disch_sabo))

d_strat_grp <- m_data %>%
  group_by(prop_strat_match) %>%
  summarise(mean_age = mean(age),
            pct_female = mean(gender),
            mean_caredays = mean(caredays),
            mean_n_op = mean(n_op),
            mean_n_diag = mean(n_diag),
            mean_days_since_prev = mean(days_since_prev,na.rm=T),
            mean_count_ov_unplan = mean(count_yr_ov_unplanned),
            mean_count_yr = mean(count_yr),
            mean_count_yr_unplan = mean(count_yr_unplanned),
            mean_dischsabo = mean(disch_sabo),
            n_dischsabo = sum(disch_sabo),
            pct_unplanreadmit30 = mean(unplanreadmit30),
            pct_mort30 = mean(mort30),
            mean_hc = mean(n_hc),
            mean_htj = mean(n_htj),
            n = n()) %>%
  pivot_longer(cols = -c(prop_strat_match))

d_props <- m_data %>%
  group_by(disch_sabo) %>%
  summarise(mean_age = mean(age),
            pct_female = mean(gender),
            mean_caredays = mean(caredays),
            mean_n_op = mean(n_op),
            mean_n_diag = mean(n_diag),
            mean_days_since_prev = mean(days_since_prev,na.rm=T),
            mean_count_ov_unplan = mean(count_yr_ov_unplanned),
            mean_count_yr = mean(count_yr),
            mean_count_yr_unplan = mean(count_yr_unplanned),
            pct_unplanreadmit30 = mean(unplanreadmit30),
            pct_mort30 = mean(mort30),
            mean_hc = mean(n_hc),
            mean_htj = mean(n_htj),
            n = n()) %>% 
  pivot_longer(cols = -disch_sabo) %>%
  mutate(prop_strat = "Overall") %>%
  bind_rows(d_strat) %>%
  mutate(prop_strat = factor(prop_strat_match,levels = c("Overall",levels(d_strat$prop_strat_match))),
         prop_strat_n = as.numeric(prop_strat_match)-1,
         discharge = ifelse(disch_sabo == 1,"NH","Home"))


d_props %>%
  #filter(!name %in% c("pct_mort30","pct_unplanreadmit30")) %>% 
ggplot(aes(x = prop_strat_n,y=value,color=discharge))+
  geom_point() +
  scale_x_continuous(breaks = seq(0,10,1)) +
  facet_wrap(~name,scales = "free",ncol = 2) +
  theme(axis.text.x = element_text(angle = 90))
```

# Pseudo r-squared

```
Pseudo.R2=function(object){
  stopifnot(object$family$family == "binomial")
  object0 = update(object, ~ 1)
  wt <- object$prior.weights # length(wt)
      y = object$y # weighted
  ones = round(y*wt)
  zeros = wt-ones
  fv <- object$fitted.values   # length(fv)
      if (is.null(object$na.action)) fv0 <- object0$fitted.values else
        fv0 <- object0$fitted.values[-object$na.action] # object may have missing values
  resp <- cbind(ones, zeros)
  Y <- apply(resp, 1, function(x) {c(rep(1, x[1]), rep(0, x[2]))} )
  if (is.list(Y)) Y <- unlist(Y) else Y <- c(Y)
  # length(Y); sum(Y)
  fv.exp <- c(apply(cbind(fv, wt), 1, function(x) rep(x[1], x[2])))
  if (is.list(fv.exp)) fv.exp <- unlist(fv.exp) else fv.exp <- c(fv.exp)
  # length(fv.exp)
  fv0.exp <- c(apply(cbind(fv0, wt), 1, function(x) rep(x[1], x[2])))
  if (is.list(fv0.exp)) fv0.exp <- unlist(fv0.exp) else fv0.exp <- c(fv0.exp)
  (ll = sum(log(dbinom(x=Y,size=1,prob=fv.exp))))
  (ll0 = sum(log(dbinom(x=Y,size=1,prob=fv0.exp))))

  n <- length(Y)
  G2 <- -2 * (ll0 - ll)
  McFadden.R2 <- 1 - ll/ll0
  CoxSnell.R2 <- 1 - exp((2 * (ll0 - ll))/n) # Cox & Snell / Maximum likelihood pseudo r-squared
  r2ML.max <- 1 - exp(ll0 * 2/n)
  Nagelkerke.R2 <- CoxSnell.R2/r2ML.max  # Nagelkerke / Cragg & Uhler's pseudo r-squared

  out <- c(llh = ll, llhNull = ll0, G2 = G2, McFadden = McFadden.R2,
           r2ML = CoxSnell.R2, r2CU = Nagelkerke.R2)
  out
}

  disch_test <- glm(disch_sabo ~ nh_propensity, family = "binomial",
                               data = d_eval)
  
  Pseudo.R2(disch_test)
```

```
##             llh         llhNull              G2        McFadden            r2ML 
##  -77400.2830848 -121160.5999627   87520.6337559       0.3611761       0.3155782 
##            r2CU 
##       0.4854975
```

# Generate outcome predictions

```
m_d_risk <- m_data %>%
  left_join(select(d,-names(d)[names(d) %in% names(m_data)],id))


if(file.exists("./m_sm.rda") & !reload){
  load("./m_sm.rda")
}else{

  m_sm <- generate_sparsm(m_d_risk)
  
  save(m_sm,file = "./m_sm.rda")
  
}

if(file.exists("./outcome_mod.rda") & !reload){
  load("./outcome_mod.rda")
}else{
  
  outcome_mod <- list()
  
  params = list(objective = "binary:logistic")
  outcome_mod$mort7_xgb_cv <- xgb.cv(data = xgb.DMatrix(data  = m_sm,
                                            label = m_d_risk$ts_mort_event_itt_7),
                         nrounds = 500,
                         nfold = 5,
                         prediction = T, 
                         early_stopping_rounds = 10,
                         params = params)
  
  outcome_mod$mort30_xgb_cv <- xgb.cv(data = xgb.DMatrix(data  = m_sm,
                                             label = m_d_risk$ts_mort_event_itt_30),
                         nrounds = 500,
                         nfold = 5,
                         prediction = T, 
                         early_stopping_rounds = 10,
                         params = params)
  
  outcome_mod$mort90_xgb_cv <- xgb.cv(data = xgb.DMatrix(data  = m_sm,
                                             label = m_d_risk$ts_mort_event_itt),
                         nrounds = 500,
                         nfold = 5,
                         prediction = T, 
                         early_stopping_rounds = 10,
                         params = params)
  
  outcome_mod$readmit7_xgb_cv <- xgb.cv(data = xgb.DMatrix(data  = m_sm,
                                            label = m_d_risk$ts_readmit_event_itt_7 == "Readmission"),
                         nrounds = 500,
                         nfold = 5,
                         prediction = T, 
                         early_stopping_rounds = 10,
                         params = params)
  outcome_mod$readmit30_xgb_cv <- xgb.cv(data = xgb.DMatrix(data  = m_sm,
                                             label = m_d_risk$ts_readmit_event_itt_30 == "Readmission"),
                         nrounds = 500,
                         nfold = 5,
                         prediction = T, 
                         early_stopping_rounds = 10,
                         params = params)
  outcome_mod$readmit90_xgb_cv <- xgb.cv(data = xgb.DMatrix(data  = m_sm,
                                             label = m_d_risk$ts_readmit_event_itt == "Readmission"),
                         nrounds = 500,
                         nfold = 5,
                         prediction = T, 
                         early_stopping_rounds = 10,
                         params = params)
  
    outcome_mod$any7_xgb_cv <- xgb.cv(data = xgb.DMatrix(data  = m_sm,
                                            label = m_d_risk$ts_any_event_itt_7),
                         nrounds = 500,
                         nfold = 5,
                         prediction = T, 
                         early_stopping_rounds = 10,
                         params = params)
  
  outcome_mod$any30_xgb_cv <- xgb.cv(data = xgb.DMatrix(data  = m_sm,
                                             label = m_d_risk$ts_any_event_itt_30),
                         nrounds = 500,
                         nfold = 5,
                         prediction = T, 
                         early_stopping_rounds = 10,
                         params = params)
  
  outcome_mod$any90_xgb_cv <- xgb.cv(data = xgb.DMatrix(data  = m_sm,
                                             label = m_d_risk$ts_any_event_itt),
                         nrounds = 500,
                         nfold = 5,
                         prediction = T, 
                         early_stopping_rounds = 10,
                         params = params)
  
  
  save(outcome_mod,file = "./outcome_mod.rda")
}
```

```
## Multiple eval metrics are present. Will use test_logloss for early stopping.
## Will train until test_logloss hasn't improved in 10 rounds.
## 
## [1]  train-logloss:0.048491±0.001336 test-logloss:0.049513±0.005160 
## [2]  train-logloss:0.046794±0.001297 test-logloss:0.049224±0.005269 
## [3]  train-logloss:0.045576±0.001385 test-logloss:0.049043±0.005115 
## [4]  train-logloss:0.044687±0.001405 test-logloss:0.048804±0.005116 
## [5]  train-logloss:0.043784±0.001311 test-logloss:0.048629±0.005099 
## [6]  train-logloss:0.042914±0.001384 test-logloss:0.048523±0.005191 
## [7]  train-logloss:0.042421±0.001345 test-logloss:0.048441±0.005184 
## [8]  train-logloss:0.041865±0.001383 test-logloss:0.048408±0.005210 
## [9]  train-logloss:0.041354±0.001494 test-logloss:0.048355±0.005203 
## [10] train-logloss:0.040871±0.001562 test-logloss:0.048349±0.005188 
## [11] train-logloss:0.040272±0.001546 test-logloss:0.048270±0.005199 
## [12] train-logloss:0.039936±0.001435 test-logloss:0.048291±0.005199 
## [13] train-logloss:0.039281±0.001560 test-logloss:0.048414±0.005256 
## [14] train-logloss:0.038902±0.001682 test-logloss:0.048356±0.005230 
## [15] train-logloss:0.038558±0.001649 test-logloss:0.048370±0.005276 
## [16] train-logloss:0.038216±0.001685 test-logloss:0.048403±0.005248 
## [17] train-logloss:0.037851±0.001669 test-logloss:0.048438±0.005229 
## [18] train-logloss:0.037642±0.001674 test-logloss:0.048481±0.005285 
## [19] train-logloss:0.037358±0.001660 test-logloss:0.048442±0.005271 
## [20] train-logloss:0.037089±0.001615 test-logloss:0.048453±0.005219 
## Stopping. Best iteration:
## [21] train-logloss:0.036785±0.001566 test-logloss:0.048451±0.005230
## 
## [21] train-logloss:0.036785±0.001566 test-logloss:0.048451±0.005230 
## Multiple eval metrics are present. Will use test_logloss for early stopping.
## Will train until test_logloss hasn't improved in 10 rounds.
## 
## [1]  train-logloss:0.197837±0.001618 test-logloss:0.199992±0.005848 
## [2]  train-logloss:0.192601±0.001726 test-logloss:0.197199±0.006076 
## [3]  train-logloss:0.189120±0.001739 test-logloss:0.195413±0.006085 
## [4]  train-logloss:0.186429±0.001848 test-logloss:0.194309±0.006125 
## [5]  train-logloss:0.183834±0.001938 test-logloss:0.193796±0.006403 
## [6]  train-logloss:0.181727±0.002248 test-logloss:0.193110±0.006576 
## [7]  train-logloss:0.180009±0.002272 test-logloss:0.192642±0.006620 
## [8]  train-logloss:0.178203±0.001871 test-logloss:0.192247±0.006732 
## [9]  train-logloss:0.176777±0.001650 test-logloss:0.191800±0.006723 
## [10] train-logloss:0.175674±0.001503 test-logloss:0.191384±0.006709 
## [11] train-logloss:0.174421±0.001716 test-logloss:0.191133±0.006608 
## [12] train-logloss:0.173231±0.001686 test-logloss:0.190942±0.006452 
## [13] train-logloss:0.172140±0.001423 test-logloss:0.190743±0.006350 
## [14] train-logloss:0.171180±0.001475 test-logloss:0.190584±0.006210 
## [15] train-logloss:0.170419±0.001488 test-logloss:0.190459±0.006295 
## [16] train-logloss:0.169408±0.001403 test-logloss:0.190378±0.006356 
## [17] train-logloss:0.168530±0.001134 test-logloss:0.190217±0.006389 
## [18] train-logloss:0.167620±0.001207 test-logloss:0.190101±0.006347 
## [19] train-logloss:0.167030±0.001089 test-logloss:0.189964±0.006259 
## [20] train-logloss:0.166289±0.000999 test-logloss:0.189852±0.006227 
## [21] train-logloss:0.165574±0.001263 test-logloss:0.189722±0.006190 
## [22] train-logloss:0.165048±0.001329 test-logloss:0.189678±0.006328 
## [23] train-logloss:0.164447±0.001284 test-logloss:0.189574±0.006350 
## [24] train-logloss:0.163805±0.001552 test-logloss:0.189633±0.006284 
## [25] train-logloss:0.163100±0.001719 test-logloss:0.189647±0.006254 
## [26] train-logloss:0.162527±0.001891 test-logloss:0.189647±0.006253 
## [27] train-logloss:0.161860±0.002098 test-logloss:0.189577±0.006283 
## [28] train-logloss:0.161326±0.002031 test-logloss:0.189456±0.006287 
## [29] train-logloss:0.160747±0.002102 test-logloss:0.189415±0.006392 
## [30] train-logloss:0.160106±0.002366 test-logloss:0.189348±0.006291 
## [31] train-logloss:0.159597±0.002341 test-logloss:0.189351±0.006386 
## [32] train-logloss:0.159122±0.002382 test-logloss:0.189316±0.006455 
## [33] train-logloss:0.158632±0.002317 test-logloss:0.189312±0.006488 
## [34] train-logloss:0.158324±0.002329 test-logloss:0.189213±0.006507 
## [35] train-logloss:0.157799±0.002260 test-logloss:0.189224±0.006444 
## [36] train-logloss:0.157159±0.002380 test-logloss:0.189236±0.006430 
## [37] train-logloss:0.156540±0.002196 test-logloss:0.189258±0.006482 
## [38] train-logloss:0.156230±0.002140 test-logloss:0.189221±0.006463 
## [39] train-logloss:0.155854±0.002048 test-logloss:0.189217±0.006474 
## [40] train-logloss:0.155504±0.001975 test-logloss:0.189188±0.006441 
## [41] train-logloss:0.155127±0.002027 test-logloss:0.189117±0.006434 
## [42] train-logloss:0.154675±0.002088 test-logloss:0.189039±0.006329 
## [43] train-logloss:0.154279±0.002175 test-logloss:0.188971±0.006216 
## [44] train-logloss:0.153919±0.002195 test-logloss:0.188903±0.006208 
## [45] train-logloss:0.153401±0.002025 test-logloss:0.188945±0.006111 
## [46] train-logloss:0.153015±0.002076 test-logloss:0.188889±0.006135 
## [47] train-logloss:0.152618±0.001967 test-logloss:0.188850±0.006094 
## [48] train-logloss:0.152277±0.001899 test-logloss:0.188846±0.006099 
## [49] train-logloss:0.151931±0.001870 test-logloss:0.188914±0.006058 
## [50] train-logloss:0.151667±0.001861 test-logloss:0.188893±0.006079 
## [51] train-logloss:0.151407±0.001873 test-logloss:0.188909±0.006105 
## [52] train-logloss:0.151074±0.001952 test-logloss:0.189025±0.006089 
## [53] train-logloss:0.150640±0.001925 test-logloss:0.188981±0.006110 
## [54] train-logloss:0.150160±0.002025 test-logloss:0.189026±0.006141 
## [55] train-logloss:0.149865±0.001931 test-logloss:0.189093±0.006185 
## [56] train-logloss:0.149598±0.001866 test-logloss:0.189120±0.006246 
## [57] train-logloss:0.149261±0.001917 test-logloss:0.189140±0.006202 
## Stopping. Best iteration:
## [58] train-logloss:0.148926±0.001841 test-logloss:0.189174±0.006224
## 
## [58] train-logloss:0.148926±0.001841 test-logloss:0.189174±0.006224 
## Multiple eval metrics are present. Will use test_logloss for early stopping.
## Will train until test_logloss hasn't improved in 10 rounds.
## 
## [1]  train-logloss:0.377527±0.000879 test-logloss:0.379048±0.003239 
## [2]  train-logloss:0.366862±0.000685 test-logloss:0.370523±0.003057 
## [3]  train-logloss:0.359134±0.000647 test-logloss:0.364995±0.002749 
## [4]  train-logloss:0.354145±0.000547 test-logloss:0.361509±0.002815 
## [5]  train-logloss:0.349714±0.000835 test-logloss:0.358497±0.002544 
## [6]  train-logloss:0.346297±0.000844 test-logloss:0.356277±0.002462 
## [7]  train-logloss:0.343020±0.001189 test-logloss:0.354770±0.002658 
## [8]  train-logloss:0.340356±0.000771 test-logloss:0.353311±0.002646 
## [9]  train-logloss:0.337904±0.001194 test-logloss:0.352084±0.002770 
## [10] train-logloss:0.335869±0.001433 test-logloss:0.351033±0.002518 
## [11] train-logloss:0.333849±0.001322 test-logloss:0.350219±0.002668 
## [12] train-logloss:0.332091±0.001181 test-logloss:0.349372±0.002849 
## [13] train-logloss:0.330452±0.001120 test-logloss:0.348489±0.002656 
## [14] train-logloss:0.328813±0.001108 test-logloss:0.348041±0.002627 
## [15] train-logloss:0.327673±0.001179 test-logloss:0.347453±0.002721 
## [16] train-logloss:0.326418±0.001000 test-logloss:0.346993±0.002707 
## [17] train-logloss:0.325353±0.001008 test-logloss:0.346763±0.002759 
## [18] train-logloss:0.324121±0.001099 test-logloss:0.346314±0.002781 
## [19] train-logloss:0.323160±0.001173 test-logloss:0.345741±0.002755 
## [20] train-logloss:0.322109±0.001143 test-logloss:0.345324±0.002761 
## [21] train-logloss:0.321025±0.001057 test-logloss:0.344915±0.002877 
## [22] train-logloss:0.319889±0.000837 test-logloss:0.344580±0.002855 
## [23] train-logloss:0.318958±0.001027 test-logloss:0.344288±0.002818 
## [24] train-logloss:0.318244±0.000972 test-logloss:0.344071±0.002882 
## [25] train-logloss:0.317402±0.001033 test-logloss:0.343771±0.002816 
## [26] train-logloss:0.316360±0.001141 test-logloss:0.343735±0.002736 
## [27] train-logloss:0.315535±0.001273 test-logloss:0.343430±0.002647 
## [28] train-logloss:0.314744±0.001132 test-logloss:0.343064±0.002710 
## [29] train-logloss:0.314077±0.001140 test-logloss:0.342848±0.002664 
## [30] train-logloss:0.313304±0.001212 test-logloss:0.342657±0.002745 
## [31] train-logloss:0.312329±0.001312 test-logloss:0.342595±0.002803 
## [32] train-logloss:0.311587±0.001624 test-logloss:0.342394±0.002906 
## [33] train-logloss:0.311024±0.001650 test-logloss:0.342353±0.002939 
## [34] train-logloss:0.310377±0.001601 test-logloss:0.342117±0.003077 
## [35] train-logloss:0.309589±0.001529 test-logloss:0.341906±0.003036 
## [36] train-logloss:0.308978±0.001638 test-logloss:0.341798±0.003065 
## [37] train-logloss:0.308371±0.001631 test-logloss:0.341673±0.003105 
## [38] train-logloss:0.307750±0.001708 test-logloss:0.341627±0.003138 
## [39] train-logloss:0.307194±0.001694 test-logloss:0.341462±0.003165 
## [40] train-logloss:0.306614±0.001663 test-logloss:0.341402±0.003122 
## [41] train-logloss:0.305914±0.001522 test-logloss:0.341336±0.003046 
## [42] train-logloss:0.305241±0.001505 test-logloss:0.341318±0.003112 
## [43] train-logloss:0.304446±0.001412 test-logloss:0.341323±0.002986 
## [44] train-logloss:0.303784±0.001490 test-logloss:0.341221±0.003034 
## [45] train-logloss:0.303174±0.001517 test-logloss:0.341289±0.003077 
## [46] train-logloss:0.302706±0.001516 test-logloss:0.341246±0.003118 
## [47] train-logloss:0.302153±0.001664 test-logloss:0.341154±0.003044 
## [48] train-logloss:0.301570±0.001524 test-logloss:0.341109±0.003028 
## [49] train-logloss:0.300892±0.001581 test-logloss:0.341020±0.003031 
## [50] train-logloss:0.300368±0.001526 test-logloss:0.341016±0.003038 
## [51] train-logloss:0.299928±0.001449 test-logloss:0.340941±0.003049 
## [52] train-logloss:0.299385±0.001334 test-logloss:0.340878±0.003060 
## [53] train-logloss:0.298886±0.001443 test-logloss:0.340772±0.003010 
## [54] train-logloss:0.298346±0.001717 test-logloss:0.340832±0.003148 
## [55] train-logloss:0.297816±0.001623 test-logloss:0.340753±0.003162 
## [56] train-logloss:0.297304±0.001665 test-logloss:0.340727±0.003153 
## [57] train-logloss:0.296848±0.001666 test-logloss:0.340739±0.003127 
## [58] train-logloss:0.296330±0.001581 test-logloss:0.340813±0.003080 
## [59] train-logloss:0.295796±0.001488 test-logloss:0.340769±0.003097 
## [60] train-logloss:0.295357±0.001490 test-logloss:0.340720±0.003155 
## [61] train-logloss:0.294896±0.001426 test-logloss:0.340731±0.003225 
## [62] train-logloss:0.294573±0.001444 test-logloss:0.340656±0.003280 
## [63] train-logloss:0.294183±0.001304 test-logloss:0.340607±0.003184 
## [64] train-logloss:0.293694±0.001287 test-logloss:0.340508±0.003245 
## [65] train-logloss:0.293268±0.001273 test-logloss:0.340503±0.003264 
## [66] train-logloss:0.292838±0.001188 test-logloss:0.340565±0.003163 
## [67] train-logloss:0.292469±0.001233 test-logloss:0.340540±0.003235 
## [68] train-logloss:0.292138±0.001229 test-logloss:0.340475±0.003238 
## [69] train-logloss:0.291853±0.001297 test-logloss:0.340499±0.003285 
## [70] train-logloss:0.291462±0.001257 test-logloss:0.340406±0.003274 
## [71] train-logloss:0.290987±0.001443 test-logloss:0.340430±0.003152 
## [72] train-logloss:0.290570±0.001369 test-logloss:0.340407±0.003140 
## [73] train-logloss:0.290095±0.001424 test-logloss:0.340401±0.003095 
## [74] train-logloss:0.289666±0.001541 test-logloss:0.340369±0.003215 
## [75] train-logloss:0.289151±0.001624 test-logloss:0.340391±0.003136 
## [76] train-logloss:0.288813±0.001730 test-logloss:0.340440±0.003137 
## [77] train-logloss:0.288400±0.001804 test-logloss:0.340423±0.003151 
## [78] train-logloss:0.288064±0.001771 test-logloss:0.340387±0.003122 
## [79] train-logloss:0.287590±0.001736 test-logloss:0.340339±0.003222 
## [80] train-logloss:0.287214±0.001586 test-logloss:0.340363±0.003045 
## [81] train-logloss:0.286906±0.001602 test-logloss:0.340272±0.003120 
## [82] train-logloss:0.286590±0.001611 test-logloss:0.340326±0.003056 
## [83] train-logloss:0.286231±0.001549 test-logloss:0.340420±0.003015 
## [84] train-logloss:0.285885±0.001596 test-logloss:0.340380±0.003081 
## [85] train-logloss:0.285482±0.001632 test-logloss:0.340399±0.003016 
## [86] train-logloss:0.285129±0.001639 test-logloss:0.340423±0.002956 
## [87] train-logloss:0.284777±0.001417 test-logloss:0.340405±0.002936 
## [88] train-logloss:0.284521±0.001400 test-logloss:0.340388±0.002974 
## [89] train-logloss:0.284247±0.001421 test-logloss:0.340363±0.002972 
## [90] train-logloss:0.283941±0.001152 test-logloss:0.340384±0.003001 
## Stopping. Best iteration:
## [91] train-logloss:0.283673±0.001199 test-logloss:0.340344±0.003024
## 
## [91] train-logloss:0.283673±0.001199 test-logloss:0.340344±0.003024 
## Multiple eval metrics are present. Will use test_logloss for early stopping.
## Will train until test_logloss hasn't improved in 10 rounds.
## 
## [1]  train-logloss:0.221253±0.002654 test-logloss:0.223925±0.010479 
## [2]  train-logloss:0.218223±0.002402 test-logloss:0.223469±0.010663 
## [3]  train-logloss:0.215949±0.002359 test-logloss:0.223191±0.010331 
## [4]  train-logloss:0.213953±0.002338 test-logloss:0.222895±0.010237 
## [5]  train-logloss:0.212507±0.002261 test-logloss:0.222840±0.010411 
## [6]  train-logloss:0.210967±0.002145 test-logloss:0.222910±0.010364 
## [7]  train-logloss:0.209605±0.002191 test-logloss:0.222890±0.010425 
## [8]  train-logloss:0.208456±0.002041 test-logloss:0.222787±0.010328 
## [9]  train-logloss:0.207570±0.001994 test-logloss:0.222805±0.010245 
## [10] train-logloss:0.206763±0.001941 test-logloss:0.222831±0.010188 
## [11] train-logloss:0.205776±0.002007 test-logloss:0.222847±0.010141 
## [12] train-logloss:0.204926±0.001844 test-logloss:0.222825±0.010162 
## [13] train-logloss:0.204377±0.001845 test-logloss:0.222762±0.010129 
## [14] train-logloss:0.203729±0.001843 test-logloss:0.222706±0.010193 
## [15] train-logloss:0.203011±0.001649 test-logloss:0.222691±0.010181 
## [16] train-logloss:0.202405±0.001738 test-logloss:0.222755±0.010203 
## [17] train-logloss:0.201740±0.001718 test-logloss:0.222799±0.010248 
## [18] train-logloss:0.201291±0.001879 test-logloss:0.222857±0.010249 
## [19] train-logloss:0.200794±0.001772 test-logloss:0.222993±0.010243 
## [20] train-logloss:0.200438±0.001871 test-logloss:0.223050±0.010181 
## [21] train-logloss:0.199967±0.001775 test-logloss:0.223064±0.010248 
## [22] train-logloss:0.199474±0.001785 test-logloss:0.223056±0.010219 
## [23] train-logloss:0.199010±0.001917 test-logloss:0.223149±0.010283 
## [24] train-logloss:0.198638±0.001978 test-logloss:0.223194±0.010255 
## Stopping. Best iteration:
## [25] train-logloss:0.198270±0.001980 test-logloss:0.223259±0.010229
## 
## [25] train-logloss:0.198270±0.001980 test-logloss:0.223259±0.010229 
## Multiple eval metrics are present. Will use test_logloss for early stopping.
## Will train until test_logloss hasn't improved in 10 rounds.
## 
## [1]  train-logloss:0.440578±0.000897 test-logloss:0.443593±0.003555 
## [2]  train-logloss:0.435144±0.001001 test-logloss:0.440802±0.003422 
## [3]  train-logloss:0.431241±0.000919 test-logloss:0.439203±0.003756 
## [4]  train-logloss:0.428442±0.000867 test-logloss:0.438310±0.003558 
## [5]  train-logloss:0.426070±0.000756 test-logloss:0.437851±0.003449 
## [6]  train-logloss:0.423770±0.000731 test-logloss:0.437431±0.003533 
## [7]  train-logloss:0.421810±0.000976 test-logloss:0.437099±0.003618 
## [8]  train-logloss:0.420117±0.001138 test-logloss:0.436997±0.003707 
## [9]  train-logloss:0.418757±0.001397 test-logloss:0.436828±0.003711 
## [10] train-logloss:0.417607±0.001462 test-logloss:0.436611±0.003679 
## [11] train-logloss:0.416606±0.001432 test-logloss:0.436356±0.003584 
## [12] train-logloss:0.415391±0.001341 test-logloss:0.436380±0.003566 
## [13] train-logloss:0.414798±0.001314 test-logloss:0.436341±0.003515 
## [14] train-logloss:0.413878±0.001323 test-logloss:0.436232±0.003528 
## [15] train-logloss:0.412784±0.001457 test-logloss:0.436118±0.003573 
## [16] train-logloss:0.411988±0.001499 test-logloss:0.436054±0.003617 
## [17] train-logloss:0.411255±0.001517 test-logloss:0.435974±0.003610 
## [18] train-logloss:0.410633±0.001534 test-logloss:0.435973±0.003674 
## [19] train-logloss:0.409878±0.001604 test-logloss:0.435972±0.003710 
## [20] train-logloss:0.409101±0.001456 test-logloss:0.435944±0.003677 
## [21] train-logloss:0.408555±0.001596 test-logloss:0.435871±0.003625 
## [22] train-logloss:0.407927±0.001770 test-logloss:0.435918±0.003601 
## [23] train-logloss:0.407200±0.001814 test-logloss:0.435925±0.003553 
## [24] train-logloss:0.406641±0.001680 test-logloss:0.435952±0.003611 
## [25] train-logloss:0.405916±0.001696 test-logloss:0.435911±0.003689 
## [26] train-logloss:0.405381±0.001814 test-logloss:0.435852±0.003700 
## [27] train-logloss:0.404707±0.001858 test-logloss:0.436021±0.003648 
## [28] train-logloss:0.404008±0.001675 test-logloss:0.436105±0.003678 
## [29] train-logloss:0.403506±0.001819 test-logloss:0.436169±0.003648 
## [30] train-logloss:0.402884±0.001649 test-logloss:0.436262±0.003640 
## [31] train-logloss:0.402362±0.001680 test-logloss:0.436231±0.003705 
## [32] train-logloss:0.401867±0.001504 test-logloss:0.436296±0.003712 
## [33] train-logloss:0.401399±0.001570 test-logloss:0.436307±0.003765 
## [34] train-logloss:0.400920±0.001741 test-logloss:0.436276±0.003815 
## [35] train-logloss:0.400403±0.001795 test-logloss:0.436359±0.003811 
## Stopping. Best iteration:
## [36] train-logloss:0.399962±0.001946 test-logloss:0.436380±0.003865
## 
## [36] train-logloss:0.399962±0.001946 test-logloss:0.436380±0.003865 
## Multiple eval metrics are present. Will use test_logloss for early stopping.
## Will train until test_logloss hasn't improved in 10 rounds.
## 
## [1]  train-logloss:0.590367±0.001237 test-logloss:0.592859±0.004344 
## [2]  train-logloss:0.583628±0.001283 test-logloss:0.588553±0.004198 
## [3]  train-logloss:0.578911±0.001288 test-logloss:0.585998±0.004280 
## [4]  train-logloss:0.575479±0.001479 test-logloss:0.584529±0.004112 
## [5]  train-logloss:0.572919±0.001447 test-logloss:0.583656±0.004098 
## [6]  train-logloss:0.570425±0.001415 test-logloss:0.582861±0.003982 
## [7]  train-logloss:0.568372±0.001554 test-logloss:0.582327±0.003921 
## [8]  train-logloss:0.566581±0.001513 test-logloss:0.582019±0.003761 
## [9]  train-logloss:0.564982±0.001427 test-logloss:0.581698±0.003769 
## [10] train-logloss:0.563768±0.001355 test-logloss:0.581463±0.003849 
## [11] train-logloss:0.562690±0.001281 test-logloss:0.581153±0.003743 
## [12] train-logloss:0.561593±0.001130 test-logloss:0.580939±0.003782 
## [13] train-logloss:0.560631±0.001142 test-logloss:0.580865±0.003756 
## [14] train-logloss:0.559618±0.001212 test-logloss:0.580815±0.003683 
## [15] train-logloss:0.558605±0.001209 test-logloss:0.580708±0.003838 
## [16] train-logloss:0.557663±0.001214 test-logloss:0.580629±0.003773 
## [17] train-logloss:0.556723±0.001302 test-logloss:0.580596±0.003721 
## [18] train-logloss:0.556036±0.001284 test-logloss:0.580629±0.003797 
## [19] train-logloss:0.555235±0.001386 test-logloss:0.580659±0.003901 
## [20] train-logloss:0.554475±0.001570 test-logloss:0.580558±0.003942 
## [21] train-logloss:0.553927±0.001605 test-logloss:0.580513±0.003944 
## [22] train-logloss:0.553337±0.001533 test-logloss:0.580477±0.003947 
## [23] train-logloss:0.552574±0.001620 test-logloss:0.580588±0.004084 
## [24] train-logloss:0.551605±0.001489 test-logloss:0.580620±0.004168 
## [25] train-logloss:0.550787±0.001393 test-logloss:0.580548±0.004181 
## [26] train-logloss:0.550119±0.001284 test-logloss:0.580495±0.004177 
## [27] train-logloss:0.549515±0.001579 test-logloss:0.580548±0.004166 
## [28] train-logloss:0.548936±0.001499 test-logloss:0.580603±0.004082 
## [29] train-logloss:0.548394±0.001470 test-logloss:0.580620±0.004030 
## [30] train-logloss:0.547691±0.001744 test-logloss:0.580591±0.004051 
## [31] train-logloss:0.547205±0.001927 test-logloss:0.580569±0.004053 
## Stopping. Best iteration:
## [32] train-logloss:0.546728±0.001945 test-logloss:0.580630±0.004043
## 
## [32] train-logloss:0.546728±0.001945 test-logloss:0.580630±0.004043 
## Multiple eval metrics are present. Will use test_logloss for early stopping.
## Will train until test_logloss hasn't improved in 10 rounds.
## 
## [1]  train-logloss:0.241168±0.002269 test-logloss:0.243651±0.008761 
## [2]  train-logloss:0.237847±0.002355 test-logloss:0.242800±0.009050 
## [3]  train-logloss:0.235088±0.002315 test-logloss:0.242142±0.009215 
## [4]  train-logloss:0.233075±0.002624 test-logloss:0.241883±0.009385 
## [5]  train-logloss:0.231541±0.002711 test-logloss:0.241836±0.009523 
## [6]  train-logloss:0.230127±0.002706 test-logloss:0.241707±0.009632 
## [7]  train-logloss:0.229214±0.002690 test-logloss:0.241538±0.009568 
## [8]  train-logloss:0.228150±0.002651 test-logloss:0.241564±0.009576 
## [9]  train-logloss:0.227208±0.002640 test-logloss:0.241567±0.009467 
## [10] train-logloss:0.226261±0.002554 test-logloss:0.241545±0.009631 
## [11] train-logloss:0.225330±0.002497 test-logloss:0.241522±0.009664 
## [12] train-logloss:0.224393±0.002579 test-logloss:0.241623±0.009622 
## [13] train-logloss:0.223541±0.002484 test-logloss:0.241590±0.009760 
## [14] train-logloss:0.223005±0.002371 test-logloss:0.241611±0.009721 
## [15] train-logloss:0.222265±0.002455 test-logloss:0.241600±0.009779 
## [16] train-logloss:0.221758±0.002352 test-logloss:0.241483±0.009779 
## [17] train-logloss:0.221208±0.002373 test-logloss:0.241414±0.009885 
## [18] train-logloss:0.220500±0.002634 test-logloss:0.241469±0.009994 
## [19] train-logloss:0.219853±0.002487 test-logloss:0.241508±0.009930 
## [20] train-logloss:0.219345±0.002406 test-logloss:0.241501±0.009891 
## [21] train-logloss:0.218884±0.002477 test-logloss:0.241552±0.009906 
## [22] train-logloss:0.218468±0.002483 test-logloss:0.241607±0.009909 
## [23] train-logloss:0.217938±0.002468 test-logloss:0.241567±0.009931 
## [24] train-logloss:0.217476±0.002391 test-logloss:0.241479±0.009947 
## [25] train-logloss:0.216966±0.002596 test-logloss:0.241560±0.009937 
## [26] train-logloss:0.216372±0.002648 test-logloss:0.241650±0.009994 
## Stopping. Best iteration:
## [27] train-logloss:0.216014±0.002708 test-logloss:0.241649±0.010046
## 
## [27] train-logloss:0.216014±0.002708 test-logloss:0.241649±0.010046 
## Multiple eval metrics are present. Will use test_logloss for early stopping.
## Will train until test_logloss hasn't improved in 10 rounds.
## 
## [1]  train-logloss:0.490601±0.001773 test-logloss:0.493428±0.006694 
## [2]  train-logloss:0.483422±0.001850 test-logloss:0.488278±0.006731 
## [3]  train-logloss:0.478417±0.001961 test-logloss:0.485446±0.006618 
## [4]  train-logloss:0.474476±0.002019 test-logloss:0.483687±0.006737 
## [5]  train-logloss:0.471511±0.002260 test-logloss:0.482420±0.006730 
## [6]  train-logloss:0.469067±0.002144 test-logloss:0.481781±0.006793 
## [7]  train-logloss:0.466733±0.002069 test-logloss:0.480980±0.007109 
## [8]  train-logloss:0.464716±0.002339 test-logloss:0.480413±0.007075 
## [9]  train-logloss:0.463008±0.002521 test-logloss:0.479903±0.007156 
## [10] train-logloss:0.461403±0.002154 test-logloss:0.479619±0.007114 
## [11] train-logloss:0.460053±0.002105 test-logloss:0.479329±0.007039 
## [12] train-logloss:0.458918±0.002178 test-logloss:0.479083±0.007031 
## [13] train-logloss:0.457851±0.002145 test-logloss:0.478980±0.007038 
## [14] train-logloss:0.456737±0.002046 test-logloss:0.478826±0.007234 
## [15] train-logloss:0.455844±0.002157 test-logloss:0.478879±0.007221 
## [16] train-logloss:0.454795±0.002246 test-logloss:0.478870±0.007322 
## [17] train-logloss:0.453754±0.002281 test-logloss:0.478863±0.007416 
## [18] train-logloss:0.452970±0.002230 test-logloss:0.478867±0.007335 
## [19] train-logloss:0.452107±0.002299 test-logloss:0.478835±0.007429 
## [20] train-logloss:0.451359±0.002144 test-logloss:0.478767±0.007441 
## [21] train-logloss:0.450464±0.002412 test-logloss:0.478817±0.007599 
## [22] train-logloss:0.449557±0.002414 test-logloss:0.478733±0.007725 
## [23] train-logloss:0.448785±0.002321 test-logloss:0.478781±0.007751 
## [24] train-logloss:0.448070±0.002187 test-logloss:0.478837±0.007938 
## [25] train-logloss:0.447466±0.002254 test-logloss:0.478801±0.007779 
## [26] train-logloss:0.446778±0.002283 test-logloss:0.478680±0.007773 
## [27] train-logloss:0.446148±0.002300 test-logloss:0.478721±0.007883 
## [28] train-logloss:0.445634±0.002305 test-logloss:0.478655±0.007932 
## [29] train-logloss:0.444805±0.002578 test-logloss:0.478593±0.007956 
## [30] train-logloss:0.444103±0.002519 test-logloss:0.478585±0.007934 
## [31] train-logloss:0.443398±0.002561 test-logloss:0.478493±0.007978 
## [32] train-logloss:0.442915±0.002689 test-logloss:0.478543±0.007969 
## [33] train-logloss:0.442202±0.002838 test-logloss:0.478530±0.008114 
## [34] train-logloss:0.441473±0.002864 test-logloss:0.478515±0.008143 
## [35] train-logloss:0.440940±0.002904 test-logloss:0.478592±0.008243 
## [36] train-logloss:0.440402±0.003042 test-logloss:0.478588±0.008232 
## [37] train-logloss:0.439735±0.002997 test-logloss:0.478589±0.008257 
## [38] train-logloss:0.439102±0.003256 test-logloss:0.478657±0.008217 
## [39] train-logloss:0.438637±0.003207 test-logloss:0.478633±0.008192 
## [40] train-logloss:0.438232±0.003159 test-logloss:0.478680±0.008196 
## Stopping. Best iteration:
## [41] train-logloss:0.437712±0.003179 test-logloss:0.478599±0.008170
## 
## [41] train-logloss:0.437712±0.003179 test-logloss:0.478599±0.008170 
## Multiple eval metrics are present. Will use test_logloss for early stopping.
## Will train until test_logloss hasn't improved in 10 rounds.
## 
## [1]  train-logloss:0.635158±0.000446 test-logloss:0.637296±0.001479 
## [2]  train-logloss:0.624816±0.000496 test-logloss:0.629195±0.001668 
## [3]  train-logloss:0.617538±0.000597 test-logloss:0.624125±0.002038 
## [4]  train-logloss:0.612463±0.000674 test-logloss:0.620654±0.001898 
## [5]  train-logloss:0.608682±0.000669 test-logloss:0.618121±0.002076 
## [6]  train-logloss:0.605588±0.000733 test-logloss:0.616264±0.002232 
## [7]  train-logloss:0.602992±0.000864 test-logloss:0.614827±0.002138 
## [8]  train-logloss:0.600629±0.001200 test-logloss:0.613864±0.002244 
## [9]  train-logloss:0.598469±0.001150 test-logloss:0.613088±0.002429 
## [10] train-logloss:0.596699±0.000878 test-logloss:0.612129±0.002417 
## [11] train-logloss:0.595001±0.001027 test-logloss:0.611596±0.002514 
## [12] train-logloss:0.593241±0.001126 test-logloss:0.611064±0.002361 
## [13] train-logloss:0.591842±0.000835 test-logloss:0.610522±0.002331 
## [14] train-logloss:0.590385±0.000849 test-logloss:0.610181±0.002271 
## [15] train-logloss:0.588907±0.001066 test-logloss:0.609981±0.002087 
## [16] train-logloss:0.587796±0.001056 test-logloss:0.609735±0.002202 
## [17] train-logloss:0.586587±0.000728 test-logloss:0.609372±0.002178 
## [18] train-logloss:0.585527±0.000909 test-logloss:0.609001±0.002236 
## [19] train-logloss:0.584461±0.000776 test-logloss:0.608722±0.002181 
## [20] train-logloss:0.583714±0.000700 test-logloss:0.608351±0.002314 
## [21] train-logloss:0.582538±0.000672 test-logloss:0.608175±0.002184 
## [22] train-logloss:0.581477±0.000671 test-logloss:0.607994±0.002319 
## [23] train-logloss:0.580561±0.000739 test-logloss:0.607937±0.002323 
## [24] train-logloss:0.579695±0.000673 test-logloss:0.607841±0.002417 
## [25] train-logloss:0.578728±0.000663 test-logloss:0.607731±0.002546 
## [26] train-logloss:0.577475±0.000491 test-logloss:0.607606±0.002536 
## [27] train-logloss:0.576717±0.000476 test-logloss:0.607508±0.002434 
## [28] train-logloss:0.575858±0.000467 test-logloss:0.607408±0.002401 
## [29] train-logloss:0.575231±0.000423 test-logloss:0.607223±0.002508 
## [30] train-logloss:0.574453±0.000423 test-logloss:0.607124±0.002603 
## [31] train-logloss:0.573686±0.000522 test-logloss:0.607003±0.002509 
## [32] train-logloss:0.573121±0.000462 test-logloss:0.606869±0.002467 
## [33] train-logloss:0.572495±0.000425 test-logloss:0.606761±0.002324 
## [34] train-logloss:0.571896±0.000402 test-logloss:0.606703±0.002231 
## [35] train-logloss:0.571235±0.000460 test-logloss:0.606654±0.002260 
## [36] train-logloss:0.570449±0.000578 test-logloss:0.606578±0.002255 
## [37] train-logloss:0.569778±0.000721 test-logloss:0.606552±0.002293 
## [38] train-logloss:0.569110±0.000706 test-logloss:0.606621±0.002399 
## [39] train-logloss:0.568515±0.000643 test-logloss:0.606585±0.002339 
## [40] train-logloss:0.568030±0.000709 test-logloss:0.606523±0.002297 
## [41] train-logloss:0.567303±0.000635 test-logloss:0.606490±0.002258 
## [42] train-logloss:0.566527±0.000734 test-logloss:0.606489±0.002267 
## [43] train-logloss:0.566102±0.000705 test-logloss:0.606468±0.002212 
## [44] train-logloss:0.565675±0.000811 test-logloss:0.606482±0.002214 
## [45] train-logloss:0.565088±0.000771 test-logloss:0.606425±0.002157 
## [46] train-logloss:0.564524±0.000812 test-logloss:0.606459±0.002231 
## [47] train-logloss:0.563859±0.000881 test-logloss:0.606403±0.002180 
## [48] train-logloss:0.563380±0.000932 test-logloss:0.606380±0.002186 
## [49] train-logloss:0.562831±0.000848 test-logloss:0.606321±0.002216 
## [50] train-logloss:0.562199±0.001024 test-logloss:0.606374±0.002338 
## [51] train-logloss:0.561784±0.001117 test-logloss:0.606382±0.002379 
## [52] train-logloss:0.561289±0.001193 test-logloss:0.606486±0.002350 
## [53] train-logloss:0.560729±0.001313 test-logloss:0.606432±0.002340 
## [54] train-logloss:0.560221±0.001220 test-logloss:0.606443±0.002288 
## [55] train-logloss:0.559788±0.001295 test-logloss:0.606489±0.002256 
## [56] train-logloss:0.559239±0.001249 test-logloss:0.606490±0.002247 
## [57] train-logloss:0.558845±0.001299 test-logloss:0.606426±0.002271 
## [58] train-logloss:0.558302±0.001266 test-logloss:0.606403±0.002270 
## Stopping. Best iteration:
## [59] train-logloss:0.557808±0.001185 test-logloss:0.606402±0.002391
## 
## [59] train-logloss:0.557808±0.001185 test-logloss:0.606402±0.002391
```

```
  preds <- data.frame(mort7_pred = unlist(outcome_mod$mort7_xgb_cv$cv_predict),
                      mort30_pred = unlist(outcome_mod$mort30_xgb_cv$cv_predict),
                      mort90_pred = unlist(outcome_mod$mort90_xgb_cv$cv_predict),
                      readmit7_pred = unlist(outcome_mod$readmit7_xgb_cv$cv_predict),
                      readmit30_pred = unlist(outcome_mod$readmit30_xgb_cv$cv_predict),
                      readmit90_pred = unlist(outcome_mod$readmit90_xgb_cv$cv_predict),
                      any7_pred = unlist(outcome_mod$any7_xgb_cv$cv_predict),
                      any30_pred = unlist(outcome_mod$any30_xgb_cv$cv_predict),
                      any90_pred = unlist(outcome_mod$any90_xgb_cv$cv_predict))
  
  
  m_data <- m_data %>%
    as_tibble() %>%
    ungroup() %>%
    bind_cols(preds)
```

# Matching table

```
meanfun <- function(data, i){
  d <- data[i]
  return(mean(d,na.rm=T))   
}

boot_cis <- function(d, s = meanfun, r=100,rd = 2,paste = T){
  bo <- boot(d, statistic=s, R=r)
  bci <- boot.ci(bo, conf=0.95,type = "perc")
  
  if(paste){
    o <- paste0(round(bci$t0,rd)," (",
              round(bci$percent[4],rd),"-",
              round(bci$percent[5],rd),")")
  } else{
    o <- list(bci$t0,
              bci$percent[4],
              bci$percent[5])
  }
  
  return(o)
}

m_strat <- m_data %>%
  group_by(disch_sabo) %>%
  summarise(n = as.character(n()),
            mean_age = boot_cis(age),
            pct_female = boot_cis(gender),
            mean_caredays = boot_cis(caredays),
            mean_n_op = boot_cis(n_op),
            mean_n_diag = boot_cis(n_diag),
            mean_days_since_prev = boot_cis(days_since_prev),
            mean_count_yr = boot_cis(count_yr),
            mean_count_yr_unplan = boot_cis(count_yr_unplanned),
            mean_hc_prev = boot_cis(n_hc), 
            mean_htj_prev = boot_cis(n_htj), 
            mean_amb_unplanned = boot_cis(count_yr_ov_unplanned),
            mean_amb_planned = boot_cis(count_yr_ov_planned),
            pct_unplanreadmit30 = boot_cis(unplanreadmit30),
            pct_mort30 = boot_cis(mort30)) %>%
  pivot_longer(cols = -c(disch_sabo)) %>%
  mutate(discharge = ifelse(disch_sabo == 1,"NH","Home"))

d_strat <- d %>%
  group_by(disch_sabo) %>%
  summarise(n = as.character(n()),
            mean_age = boot_cis(age),
            pct_female = boot_cis(gender),
            mean_caredays = boot_cis(caredays),
            mean_n_op = boot_cis(n_op),
            mean_n_diag = boot_cis(n_diag),
            mean_days_since_prev = boot_cis(days_since_prev),
            mean_count_yr = boot_cis(count_yr),
            mean_count_yr_unplan = boot_cis(count_yr_unplanned),
            mean_hc_prev = boot_cis(n_hc),
            mean_htj_prev = boot_cis(n_htj),
            mean_amb_unplanned = boot_cis(count_yr_ov_unplanned),
            mean_amb_planned = boot_cis(count_yr_ov_planned),
            pct_unplanreadmit30 = boot_cis(unplanreadmit30),
            pct_mort30 = boot_cis(mort30)) %>%
  pivot_longer(cols = -c(disch_sabo)) %>%
  mutate(discharge = ifelse(disch_sabo == 1,"NH","Home"))

smd <- m_data %>%
  as_tibble() %>%
  select(disch_sabo,
         mean_age = age,
         mean_caredays = caredays,
         mean_n_op = n_op,
         mean_n_diag = n_diag,
         mean_days_since_prev = days_since_prev,
         mean_count_yr = count_yr,
         mean_count_yr_unplan = count_yr_unplanned,
         mean_hc_prev = n_hc,
         mean_htj_prev = n_htj,
         mean_amb_unplanned = count_yr_ov_unplanned,
         mean_amb_planned = count_yr_ov_planned) %>%
  pivot_longer(-disch_sabo, names_to = "variable", values_to = "value") %>%
  group_by(variable) %>%
  summarise(effectsize::cohens_d(value ~ disch_sabo, ci = 0.95)) %>%
  mutate(pasted = paste0(round(Cohens_d,3)," (",round(CI_low,3),"-",round(CI_high,3),")"))

#Balance in original data
d_strat_wide <- d_strat %>%
  select(-disch_sabo) %>%
  pivot_wider(names_from = discharge,
              values_from = value,
              names_prefix = "raw ")

#Balance in matched data
m_strat_wide <- m_strat %>%
  select(-disch_sabo) %>%
  pivot_wider(names_from = discharge,
              values_from = value,
              names_prefix = "matched ")

match_table <- bind_cols(d_strat_wide,select(m_strat_wide,-name)) %>%
  left_join(select(smd,name = variable,smd_ci = pasted))

kable(match_table)
```

| name | raw Home | raw NH | matched Home | matched NH | smd\_ci |
| --- | --- | --- | --- | --- | --- |
| n | 180396 | 50419 | 31494 | 31494 | NA |
| mean\_age | 81.99 (81.94-82.02) | 83.75 (83.67-83.81) | 82.77 (82.66-82.86) | 82.83 (82.76-82.95) | -0.008 (-0.023-0.008) |
| pct\_female | 0.6 (0.6-0.6) | 0.59 (0.59-0.6) | 0.59 (0.58-0.59) | 0.59 (0.58-0.59) | NA |
| mean\_caredays | 10.97 (10.93-11.01) | 16 (15.85-16.13) | 14.59 (14.48-14.72) | 14.8 (14.66-14.9) | -0.019 (-0.035–0.003) |
| mean\_n\_op | 2.74 (2.73-2.76) | 3.11 (3.09-3.14) | 3.09 (3.05-3.12) | 3.06 (3.03-3.1) | 0.009 (-0.007-0.024) |
| mean\_n\_diag | 5.45 (5.44-5.46) | 5.79 (5.77-5.81) | 5.53 (5.5-5.56) | 5.62 (5.58-5.65) | -0.03 (-0.045–0.014) |
| mean\_days\_since\_prev | 122.29 (121.52-123.07) | 86.96 (85.44-88.62) | 96.6 (94.55-98.64) | 97.76 (95.73-99.76) | -0.011 (-0.042-0.02) |
| mean\_count\_yr | 0.73 (0.72-0.73) | 0.59 (0.58-0.6) | 0.46 (0.45-0.47) | 0.49 (0.47-0.5) | -0.023 (-0.039–0.008) |
| mean\_count\_yr\_unplan | 0.58 (0.58-0.59) | 0.51 (0.5-0.52) | 0.38 (0.37-0.39) | 0.4 (0.39-0.41) | -0.027 (-0.043–0.011) |
| mean\_hc\_prev | 2.07 (2.04-2.1) | 0.16 (0.14-0.17) | 0.27 (0.24-0.29) | 0.25 (0.23-0.28) | 0.009 (-0.007-0.025) |
| mean\_htj\_prev | 3.99 (3.94-4.03) | 0.26 (0.24-0.29) | 0.41 (0.38-0.44) | 0.4 (0.37-0.44) | 0 (-0.015-0.016) |
| mean\_amb\_unplanned | 1.29 (1.28-1.3) | 0.98 (0.96-1) | 0.76 (0.74-0.78) | 0.81 (0.79-0.84) | -0.032 (-0.048–0.017) |
| mean\_amb\_planned | 1.94 (1.92-1.98) | 1.08 (1.04-1.13) | 1.05 (0.99-1.1) | 1.1 (1.03-1.15) | -0.011 (-0.027-0.004) |
| pct\_unplanreadmit30 | 0.21 (0.2-0.21) | 0.14 (0.14-0.14) | 0.19 (0.18-0.19) | 0.14 (0.14-0.15) | NA |
| pct\_mort30 | 0.03 (0.03-0.03) | 0.08 (0.07-0.08) | 0.04 (0.03-0.04) | 0.07 (0.07-0.08) | NA |

# Categorical predictor distributions

## Major Diagnostic Group

```
d_eval %>%
  left_join(mdc_df) %>%
  group_by(disch_sabo,mdc_name) %>%
  summarise(n = n()) %>%
  group_by(discharge = ifelse(disch_sabo == 1,"NH","Home")) %>%
  mutate(pct = n/sum(n)) %>%
  ggplot(aes(y=pct,x= mdc_name,fill = discharge)) +
  geom_bar(stat="identity", position = position_dodge()) +
  labs(y= "% of patients with MDC  (raw)") +
  theme(axis.text.x = element_text(angle = 90,hjust = 1,vjust = 0.3))
```

```
m_data %>%
  left_join(mdc_df) %>%
  group_by(disch_sabo,mdc_name) %>%
  summarise(n = n()) %>%
  group_by(discharge = ifelse(disch_sabo == 1,"NH","Home")) %>%
  mutate(pct = n/sum(n)) %>%
  ggplot(aes(y=pct,x= mdc_name,fill = discharge)) +
  geom_bar(stat="identity", position = position_dodge()) +
  labs(y= "% of patients with MDC  (raw)") +
  theme(axis.text.x = element_text(angle = 90,hjust = 1,vjust = 0.3))
```

## Hospital Ward

```
d_eval %>%
  group_by(disch_sabo,mvo_last) %>%
  summarise(n = n()) %>%
  group_by(discharge = ifelse(disch_sabo == 1,"NH","Home")) %>%
  mutate(pct = n/sum(n)) %>%
  ggplot(aes(y=pct,x= as.factor(mvo_last),fill = discharge)) +
  geom_bar(stat="identity", position = position_dodge()) +
  labs(y= "Percent of patients with ward code (raw)") +
  theme(axis.text.x = element_text(angle = 90,hjust = 1,vjust = 0.3))
```

```
  labs(x = "Hospital ward code")
```

```
## <ggplot2::labels> List of 1
##  $ x: chr "Hospital ward code"
```

```
m_data %>%
  group_by(disch_sabo,mvo_last) %>%
  summarise(n = n()) %>%
  group_by(discharge = ifelse(disch_sabo == 1,"NH","Home")) %>%
  mutate(pct = n/sum(n)) %>%
  ggplot(aes(y=pct,x= as.factor(mvo_last),fill = discharge)) +
  geom_bar(stat="identity", position = position_dodge()) +
  labs(y= "Percent of patients with ward code (raw)") +
  theme(axis.text.x = element_text(angle = 90,hjust = 1,vjust = 0.3))
```

```
  labs(x = "Hospital ward code")
```

```
## <ggplot2::labels> List of 1
##  $ x: chr "Hospital ward code"
```

These codes are a bit difficult to translate and likely have limited
interpretability outside of Sweden anyway… Ward code lists may be found
with the National Board of Health and Welfare: https://www.socialstyrelsen.se/globalassets/sharepoint-dokument/dokument-webb/klassifikationer-och-koder/sjukhuskoder-kodlista-verksamhetsomraden-2006.pdf

## Region

```
d_eval %>%
  group_by(disch_sabo,region) %>%
  summarise(n = n()) %>%
  group_by(discharge = ifelse(disch_sabo == 1,"NH","Home")) %>%
  mutate(pct = n/sum(n)) %>%
  ggplot(aes(y=pct,x= as.factor(region),fill = discharge)) +
  geom_bar(stat="identity", position = position_dodge()) +
  labs(y= "Percent of patients in region (raw)") +
  theme(axis.text.x = element_text(angle = 90,hjust = 1,vjust = 0.3)) +
  labs(x = "Region")
```

```
m_data %>%
  group_by(disch_sabo,region) %>%
  summarise(n = n()) %>%
  group_by(discharge = ifelse(disch_sabo == 1,"NH","Home")) %>%
  mutate(pct = n/sum(n)) %>%
  ggplot(aes(y=pct,x= as.factor(region),fill = discharge)) +
  geom_bar(stat="identity", position = position_dodge()) +
  labs(y= "Percent of patients in region (raw)") +
  theme(axis.text.x = element_text(angle = 90,hjust = 1,vjust = 0.3)) +
  labs(x = "Region")
```

# Propensity score distribution in raw and matched samples

```
ggplot(d_eval, aes(x = nh_propensity, 
              fill = as.factor(disch_sabo))) +
  geom_density(alpha = 0.5, colour = "grey50") +
  #geom_point(aes(y= 0,x=nh_propensity,)) +
  geom_rug(aes(color = prop_strat)) +
  scale_color_brewer(palette = "Spectral")
```

```
d_eval %>%
  group_by(prop_strat,disch_sabo) %>%
  summarise(n = n()) %>%
  pivot_wider(names_from = disch_sabo,
              values_from = n)
```

```
ggplot(m_data, aes(x = nh_propensity, 
              fill = as.factor(disch_sabo))) +
  geom_density(alpha = 0.5, colour = "grey50") +
  #geom_point(aes(y= 0,x=nh_propensity,)) +
  geom_rug(aes(color = prop_strat)) +
  scale_color_brewer(palette = "Spectral")
```

```
m_data %>%
  group_by(prop_strat,disch_sabo) %>%
  summarise(n = n()) %>%
  pivot_wider(names_from = disch_sabo,
              values_from = n)
```

# Propensity score variable description

```
description <- c("caredays" = "Duration of hospital stay",  
                 "diagsec" = "Secondary diagnosis", 
                 "age" = "Patient age", 
                 "region" = "Region", 
                 "muni" = "Municipality", 
                 "mvo" = "Hospital ward", 
                 "date" = "Date of discharge", 
                 "prevSince" = "Days since previous hospital stay", 
                 "countYrUnplanned" = "Number of unplanned hospital stays in the last year", 
                 "countHomecareMonths" = "Number of months of home health care prior to discharge",
                 "countHomeserviceMonths" = "Number of months of home services prior to discharge",
                 "countAmbPlanned" = "Number of planned contacts with ambulatory care in the previous year",
                 "countAmbUnplanned" = "Number of unplanned contacts with ambulatory care in the previous year",
                 "countDiags" = "Number of diagnosis codes recorded during hospital stay",
                 "countInterventions" = "Number of intervention codes recorded during hospital stay",
                 "prevCaredays" = "Duration of previous hostial stay", 
                 "week" = "Week of discharge", 
                 "hosp" = "Discharging hospital", 
                 "civil" = "Patient civil status", 
                 "diagprim" = "Patient primary diagnosis", 
                 "op" = "Operation (KVÅ) code", 
                 "countYr" = "Number of hospital stays in the last year", 
                 "female" = "Sex of patient", 
                 "weekday" = "Weekday of discharge", 
                 "born" = "Patient region of birth", 
                 "prevdiag" = "Primary diagnosis during previous hospital stay")

importance_matrix <- xgb.importance(propensity_xgb) %>%
  mutate(type = str_split_i(Feature,"_",1)) %>%
  left_join(as_tibble(list(type = names(description),
                      description = description)))

t <- xgb.plot.shap.summary(data = sm, model = propensity_xgb,top_n = 100)

mean_shap <- t$data %>%
  mutate(valshap = feature_value*shap_value) %>%
  group_by(Feature = feature) %>%
  summarise(mean_shap = mean(valshap))

importance_matrix <- importance_matrix %>%
  left_join(mean_shap) %>%
  select(description,Feature,Gain,Cover,mean_shap)

vars <- colnames(m_sm)[colnames(m_sm) %in% importance_matrix$Feature]

smd_matched <- m_sm[,vars] %>%
  as.matrix() %>%
  as_tibble() %>%
  bind_cols(select(m_data,disch_sabo)) %>%
  pivot_longer(-disch_sabo, names_to = "variable", values_to = "value") %>%
  group_by(variable) %>%
  summarise(effectsize::cohens_d(value ~ disch_sabo)) %>%
  left_join(importance_matrix,by = c("variable"="Feature")) %>%
  mutate(smd_95ci = paste0(round(Cohens_d,3)," (",round(CI_low,3),":",round(CI_high,3),")")) %>%
  transmute(variable,
            description,
            smd_95ci,
            Gain = round(Gain,5),
            Cover = round(Cover,5),
            mean_shap = round(mean_shap,5),
            Cohens_d) %>%
  arrange(desc(Gain))
```

## Variable summary

```
importance_matrix %>%
  group_by(description) %>%
  summarise(n_values = n(),
            sum_gain = sum(Gain)) %>%
  arrange(desc(sum_gain)) %>%
  kable()
```

| description | n\_values | sum\_gain |
| --- | --- | --- |
| Number of months of home services prior to discharge | 1 | 0.2063592 |
| Secondary diagnosis | 605 | 0.1898181 |
| Duration of hospital stay | 1 | 0.1226844 |
| Region | 21 | 0.0535806 |
| Number of months of home health care prior to discharge | 1 | 0.0526059 |
| Municipality | 201 | 0.0499560 |
| Operation (KVÅ) code | 314 | 0.0446787 |
| Patient age | 1 | 0.0444714 |
| Hospital ward | 35 | 0.0369663 |
| Duration of previous hostial stay | 1 | 0.0281324 |
| Number of unplanned contacts with ambulatory care in the previous year | 1 | 0.0278809 |
| Date of discharge | 1 | 0.0235106 |
| Discharging hospital | 70 | 0.0224705 |
| Days since previous hospital stay | 1 | 0.0162427 |
| Patient primary diagnosis | 195 | 0.0136386 |
| Week of discharge | 1 | 0.0125138 |
| Number of intervention codes recorded during hospital stay | 1 | 0.0101896 |
| Number of planned contacts with ambulatory care in the previous year | 1 | 0.0075637 |
| Number of diagnosis codes recorded during hospital stay | 1 | 0.0073374 |
| Weekday of discharge | 7 | 0.0066980 |
| Patient civil status | 4 | 0.0066910 |
| Number of unplanned hospital stays in the last year | 1 | 0.0058417 |
| Primary diagnosis during previous hospital stay | 89 | 0.0036634 |
| Sex of patient | 1 | 0.0029272 |
| Number of hospital stays in the last year | 1 | 0.0018558 |
| Patient region of birth | 4 | 0.0017219 |

## Detailed variable table

Note: Higher SHAP values indicate a marginal, linear association with
a higher likelihood of discharge to NH. Note that many ICD codes were
masked by the providers of the registry data to maintain patient
anonymity.

```
datatable(select(smd_matched,-Cohens_d))
```

## Partial dependence plot

```
xgb.plot.shap(sm, model = propensity_xgb,top_n = 5)
```

# SMD for all predictors

```
smd_matched %>%
  ggplot(aes(x=Cohens_d,y=reorder(variable,Gain))) +
  geom_point() +
  theme(
    axis.text.y = element_blank(),
    axis.ticks.y = element_blank() )+
  
  labs(y = "Parameter in propensity score model",
       x = "Standardized Mean Difference")
```

# Main analysis

## Estimate models

```
if(file.exists("./cif.rda") & !reload){
  load("./cif.rda")
  load("./hr.rda")
}else{
  
  cif <- list()
  hr <- list()
  
  cif$mort <- tidycmprsk::cuminc(Surv(ts_mort_days_itt, 
                                      as.factor(ts_mort_event_itt)) ~ disch_sabo, 
                     cluster = lopnr, 
                     d_eval)
  
  cif$mort_prop <- tidycmprsk::cuminc(Surv(ts_mort_days_itt, 
                                           as.factor(ts_mort_event_itt)) ~ disch_sabo,
                     cluster = subclass, 
                     m_data)
  
  hr$mort7 <- coxph(Surv(ts_mort_days_itt_7,
                         ts_mort_event_itt_7) ~ disch_sabo,
                    cluster = lopnr,
                    data = d_eval)
  
  hr$mort30 <- coxph(Surv(ts_mort_days_itt_30,
                         ts_mort_event_itt_30) ~ disch_sabo,
                    cluster = lopnr,
                    data = d_eval)
  
  hr$mort90 <- coxph(Surv(ts_mort_days_itt,
                         ts_mort_event_itt) ~ disch_sabo,
                    cluster = lopnr,
                    data = d_eval)
  
  hr$mort7_prop <- coxph(Surv(ts_mort_days_itt_7,
                         ts_mort_event_itt_7) ~ disch_sabo,
                    cluster = subclass,
                    data = m_data)
  
  hr$mort30_prop <- coxph(Surv(ts_mort_days_itt_30,
                         ts_mort_event_itt_30) ~ disch_sabo,
                    cluster = subclass,
                    data = m_data)
  hr$mort90_prop <- coxph(Surv(ts_mort_days_itt,
                         ts_mort_event_itt) ~ disch_sabo,
                    cluster = subclass,
                    data = m_data)
  
  hr$mort7_dr <- coxph(Surv(ts_mort_days_itt_7,
                         ts_mort_event_itt_7) ~ disch_sabo + 
                         mort7_pred,
                    cluster = subclass,
                    data = m_data)
  
  hr$mort30_dr <- coxph(Surv(ts_mort_days_itt_30,
                         ts_mort_event_itt_30) ~ disch_sabo + mort30_pred,
                    cluster = subclass,
                    data = m_data)
  hr$mort90_dr <- coxph(Surv(ts_mort_days_itt,
                         ts_mort_event_itt) ~ disch_sabo + mort90_pred,
                    cluster = subclass,
                    data = m_data)
  
  ## Readmission
  
  
  cif$readmit <- tidycmprsk::cuminc(Surv(ts_readmit_days_itt, 
                                                     ts_readmit_event_itt) ~ disch_sabo,
                     cluster = lopnr, 
                     d_eval)
  
  cif$readmit_prop <- tidycmprsk::cuminc(Surv(ts_readmit_days_itt, 
                                              ts_readmit_event_itt) ~ disch_sabo,
                     cluster = subclass, 
                     m_data)
  
  # cif$readmit_prop_dr <- tidycmprsk::cuminc(Surv(ts_readmit_days_itt, 
  #                                                         as.numeric(ts_readmit_event_itt)) ~ disch_sabo + readmit30_pred,
  #                    cluster = subclass, 
  #                    m_data)
  
  library(fastcmprsk)
  
  hr$readmit7 <- fastCrr(Crisk(ts_readmit_days_itt_7,ts_readmit_event_itt_7,
                         failcode = "Readmission",
                         cencode = "Censored") ~ disch_sabo,
                    data = d_eval)
  hr$readmit30 <- fastCrr(Crisk(ts_readmit_days_itt_30,ts_readmit_event_itt_30,
                         failcode = "Readmission",
                         cencode = "Censored") ~ disch_sabo,
                    data = d_eval)
  hr$readmit90 <- fastCrr(Crisk(ts_readmit_days_itt,ts_readmit_event_itt,
                         failcode = "Readmission",
                         cencode = "Censored") ~ disch_sabo,
                    data = d_eval)
  
  hr$readmit7_prop <- fastCrr(Crisk(ts_readmit_days_itt_7,ts_readmit_event_itt_7,
                         failcode = "Readmission",
                         cencode = "Censored") ~ disch_sabo,
                    data = m_data)
  hr$readmit30_prop <- fastCrr(Crisk(ts_readmit_days_itt_30,ts_readmit_event_itt_30,
                         failcode = "Readmission",
                         cencode = "Censored") ~ disch_sabo,
                    data = m_data)
  hr$readmit90_prop <- fastCrr(Crisk(ts_readmit_days_itt,ts_readmit_event_itt,
                         failcode = "Readmission",
                         cencode = "Censored") ~ disch_sabo,
                    data = m_data)
  
  hr$readmit7_dr <- fastCrr(Crisk(ts_readmit_days_itt_7,ts_readmit_event_itt_7,
                         failcode = "Readmission",
                         cencode = "Censored") ~ disch_sabo + readmit7_pred,
                    data = m_data)
  hr$readmit30_dr <- fastCrr(Crisk(ts_readmit_days_itt_30,ts_readmit_event_itt_30,
                         failcode = "Readmission",
                         cencode = "Censored") ~ disch_sabo + readmit30_pred,
                    data = m_data)
  hr$readmit90_dr <- fastCrr(Crisk(ts_readmit_days_itt,ts_readmit_event_itt,
                         failcode = "Readmission",
                         cencode = "Censored") ~ disch_sabo + readmit90_pred,
                    data = m_data)
  
  
  # Composite outcome
  
    cif$any <- tidycmprsk::cuminc(Surv(ts_any_days_itt, 
                                      as.factor(ts_any_event_itt)) ~ disch_sabo, 
                     cluster = lopnr, 
                     d_eval)
  
  cif$any_prop <- tidycmprsk::cuminc(Surv(ts_any_days_itt, 
                                           as.factor(ts_any_event_itt)) ~ disch_sabo,
                     cluster = subclass, 
                     m_data)
  
  hr$any7 <- coxph(Surv(ts_any_days_itt_7,
                         ts_any_event_itt_7) ~ disch_sabo,
                    cluster = lopnr,
                    data = d_eval)
  
  hr$any30 <- coxph(Surv(ts_any_days_itt_30,
                         ts_any_event_itt_30) ~ disch_sabo,
                    cluster = lopnr,
                    data = d_eval)
  
  hr$any90 <- coxph(Surv(ts_any_days_itt,
                         ts_any_event_itt) ~ disch_sabo,
                    cluster = lopnr,
                    data = d_eval)
  
  hr$any7_prop <- coxph(Surv(ts_any_days_itt_7,
                         ts_any_event_itt_7) ~ disch_sabo,
                    cluster = subclass,
                    data = m_data)
  
  hr$any30_prop <- coxph(Surv(ts_any_days_itt_30,
                         ts_any_event_itt_30) ~ disch_sabo,
                    cluster = subclass,
                    data = m_data)
  hr$any90_prop <- coxph(Surv(ts_any_days_itt,
                         ts_any_event_itt) ~ disch_sabo,
                    cluster = subclass,
                    data = m_data)
  
  hr$any7_dr <- coxph(Surv(ts_any_days_itt_7,
                         ts_any_event_itt_7) ~ disch_sabo + 
                         any7_pred,
                    cluster = subclass,
                    data = m_data)
  
  hr$any30_dr <- coxph(Surv(ts_any_days_itt_30,
                         ts_any_event_itt_30) ~ disch_sabo + any30_pred,
                    cluster = subclass,
                    data = m_data)
  hr$any90_dr <- coxph(Surv(ts_any_days_itt,
                         ts_any_event_itt) ~ disch_sabo + any90_pred,
                    cluster = subclass,
                    data = m_data)
  
  save(cif,file = "./cif.rda")
  save(hr,file = "./hr.rda")
}
```

## Plot cumulative incidence curves

```
cif$mort %>%
  ggcuminc(outcome = "TRUE") +
  add_confidence_interval() +
  labs(y = "Mortality cumulative incidence (raw)",
       x = "Days since discharge") + 
  scale_color_manual(values = c("red", "blue")) +
  scale_fill_manual(values = c("red", "blue")) +
  geom_vline(xintercept = c(7,30,90))
```

```
cif$mort_prop %>%
  ggcuminc(outcome = "TRUE") +
  add_confidence_interval() +
  labs(y = "Mortality cumulative incidence (matched)",
       x = "Days since discharge") + 
  scale_color_manual(values = c("red", "blue")) +
  scale_fill_manual(values = c("red", "blue")) +
  geom_vline(xintercept = c(7,30,90))
```

```
cif$readmit %>%
  ggcuminc(outcome = "Readmission") +
  add_confidence_interval() +
  labs(y = "Readmission cumulative incidence (raw)",
       x = "Days since discharge") + 
  scale_color_manual(values = c("red", "blue")) +
  scale_fill_manual(values = c("red", "blue")) +
  geom_vline(xintercept = c(7,30,90))
```

```
cif$readmit_prop %>%
  ggcuminc(outcome = "Readmission") +
  add_confidence_interval() +
  labs(y = "Readmission cumulative incidence (matched)",
       x = "Days since discharge") + 
  scale_color_manual(values = c("red", "blue")) +
  scale_fill_manual(values = c("red", "blue")) +
  geom_vline(xintercept = c(7,30,90))
```

```
cif$any %>%
  ggcuminc(outcome = "TRUE") +
  add_confidence_interval() +
  labs(y = "Composite cumulative incidence (raw)",
       x = "Days since discharge") + 
  scale_color_manual(values = c("red", "blue")) +
  scale_fill_manual(values = c("red", "blue")) +
  geom_vline(xintercept = c(7,30,90))
```

```
cif$any_prop %>%
  ggcuminc(outcome = "TRUE") +
  add_confidence_interval() +
  labs(y = "Composite cumulative incidence (matched)",
       x = "Days since discharge") + 
  scale_color_manual(values = c("red", "blue")) +
  scale_fill_manual(values = c("red", "blue")) +
  geom_vline(xintercept = c(7,30,90))
```

## Hazard ratio table

```
paste_cox_ci <- function(mod,r=3){
  paste0(round(exp(coef(mod)["disch_sabo"]),r), " (",
        round(exp(confint(mod)["disch_sabo",1]),r),"-",
        round(exp(confint(mod)["disch_sabo",2]),r),")")
}

paste_crr_ci <- function(mod,r=3){
  
  ci <- confint(mod)
  if(first(class(ci)) == "matrix"){
    ci <- ci[1,]
  }

  paste0(round(exp(coef(mod)[1]),r), " (",
        round(exp(ci[1]),r),"-",
        round(exp(ci[2]),r),")")
}

if(file.exists("./outcome_table.rda") & !reload){
  load("./outcome_table.rda")
}else{

raw <- data.frame("raw" = c(paste_cox_ci(hr$mort7),
                            paste_cox_ci(hr$mort30),
                            paste_cox_ci(hr$mort90),
                            paste_crr_ci(hr$readmit7),
                            paste_crr_ci(hr$readmit30),
                            paste_crr_ci(hr$readmit90),
                            paste_crr_ci(hr$any7),
                            paste_crr_ci(hr$any30),
                            paste_crr_ci(hr$any90)))

matched <- data.frame("matched" = c(paste_cox_ci(hr$mort7_prop),
                            paste_cox_ci(hr$mort30_prop),
                            paste_cox_ci(hr$mort90_prop),
                            paste_crr_ci(hr$readmit7_prop),
                            paste_crr_ci(hr$readmit30_prop),
                            paste_crr_ci(hr$readmit90_prop),
                            paste_crr_ci(hr$any7_prop),
                            paste_crr_ci(hr$any30_prop),
                            paste_crr_ci(hr$any90_prop)))

doublerobust <- data.frame("doublerobust" = c(paste_cox_ci(hr$mort7_dr),
                            paste_cox_ci(hr$mort30_dr),
                            paste_cox_ci(hr$mort90_dr),
                            paste_crr_ci(hr$readmit7_dr),
                            paste_crr_ci(hr$readmit30_dr),
                            paste_crr_ci(hr$readmit90_dr),
                            paste_crr_ci(hr$any7_dr),
                            paste_crr_ci(hr$any30_dr),
                            paste_crr_ci(hr$any90_dr)))

outcome_table <- data.frame("outcome" = c(rep("Mortality",3),
                                          rep("Readmission",3),
                                          rep("Composite",3)),
                            "Time" = c(rep(c("7","30","90"),3))) %>%
  bind_cols(raw) %>%
  bind_cols(matched) %>%
  bind_cols(doublerobust)

  save(outcome_table,file = "./outcome_table.rda")

}

kable(outcome_table)
```

| outcome | Time | raw | matched | doublerobust |
| --- | --- | --- | --- | --- |
| Mortality | 7 | 2.311 (2.092-2.553) | 1.84 (1.549-2.185) | 1.786 (1.502-2.124) |
| Mortality | 30 | 2.384 (2.29-2.483) | 2.007 (1.871-2.153) | 1.979 (1.842-2.126) |
| Mortality | 90 | 2.043 (1.992-2.094) | 1.899 (1.819-1.982) | 1.936 (1.851-2.023) |
| Readmission | 7 | 0.574 (0.548-0.601) | 0.671 (0.631-0.714) | 0.672 (0.632-0.716) |
| Readmission | 30 | 0.653 (0.635-0.672) | 0.742 (0.717-0.768) | 0.732 (0.707-0.757) |
| Readmission | 90 | 0.708 (0.694-0.722) | 0.81 (0.786-0.835) | 0.794 (0.77-0.818) |
| Composite | 7 | 0.68 (0.653-0.707) | 0.77 (0.725-0.818) | 0.772 (0.727-0.82) |
| Composite | 30 | 0.869 (0.85-0.888) | 0.944 (0.912-0.977) | 0.928 (0.897-0.961) |
| Composite | 90 | 0.976 (0.961-0.991) | 1.069 (1.042-1.096) | 1.051 (1.024-1.079) |
